# Ionizable Lipids with Optimized Linkers Enable Lung-Specific, Lipid Nanoparticle-Mediated mRNA Delivery for Treatment of Metastatic Lung Tumors

**DOI:** 10.1021/acsnano.4c18636

**Published:** 2025-02-06

**Authors:** Gonna Somu Naidu, Riccardo Rampado, Preeti Sharma, Assaf Ezra, Govinda Reddy Kundoor, Dor Breier, Dan Peer

**Affiliations:** † Laboratory of Precision Nanomedicine, Shmunis School of Biomedicine and Cancer Research, Tel Aviv University, Tel Aviv-Yafo 69978, Israel; ‡ Department of Materials Sciences and Engineering, Tel Aviv University, Tel Aviv-Yafo 69978, Israel; § Center for Nanoscience and Nanotechnology, Tel Aviv University, Tel Aviv-Yafo 69978, Israel; ∥ Cancer Biology Research Center, Tel Aviv University, Tel Aviv-Yafo 69978, Israel; ⊥ Department of Pharmaceutical Sciences, University of Padova, Padova 35131, Italy

**Keywords:** ionizable lipids, lipid nanoparticle, mRNA delivery, biodegradable linkers, lung delivery, genetic medicines

## Abstract

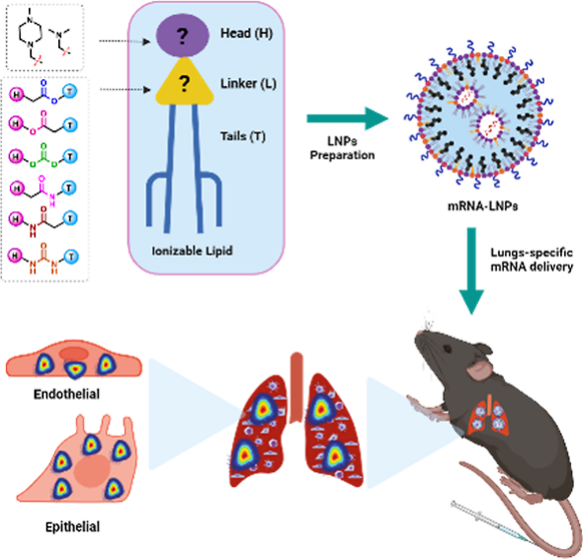

Lipid nanoparticles (LNPs) have emerged as a groundbreaking delivery system for vaccines and therapeutic mRNAs. Ionizable lipids are the most pivotal component of LNPs due to their ability to electrostatically interact with mRNA, allowing its encapsulation while concurrently enabling its endosomal escape following cellular internalization. Thus, extensive research has been performed to optimize the ionizable lipid structure and to develop formulations that are well tolerated and allow efficient targeting of different organs that result in a high and sustained mRNA expression. However, one facet of the ionizable lipids’ structure has been mostly overlooked: the linker segment between the ionizable headgroup and their tails. Here, we screened a rationally designed library of ionizable lipids with different biodegradable linkers. We extensively characterized LNPs formulated using these ionizable lipids and elucidated how these minor structural changes in the ionizable lipids structure radically influenced the LNPs’ biodistribution in vivo. We showed how the use of amide and urea linkers can modulate the LNPs’ p*K*_a_, resulting in an improved specificity for lung transfection. Finally, we demonstrated how one of these lipids (lipid 35) that form LNPs entrapping a bacterial toxin [pseudomonas exotoxin A (mmPE)] in the form of an mRNA reduced tumor burden and significantly increased the survival of mice with lung metastasis.

## Introduction

Messenger RNA (mRNA) delivery has emerged as an innovative approach to prevent and treat a variety of pathological conditions such as cancers, infections, and hereditary genetic diseases.^1−5^ The US Food and Drug Administration (FDA)’s recent approval of three mRNA vaccines (Spikevax, Comirnaty, and mRESVIA) for COVID-19 and respiratory syncytial virus prevention significantly enhanced research efforts in this field.^6,7^ Furthermore, several other mRNA-based therapeutics have been developed and are currently under clinical evaluation for preventing and treating various diseases.^8,9^

**Figure 1:**
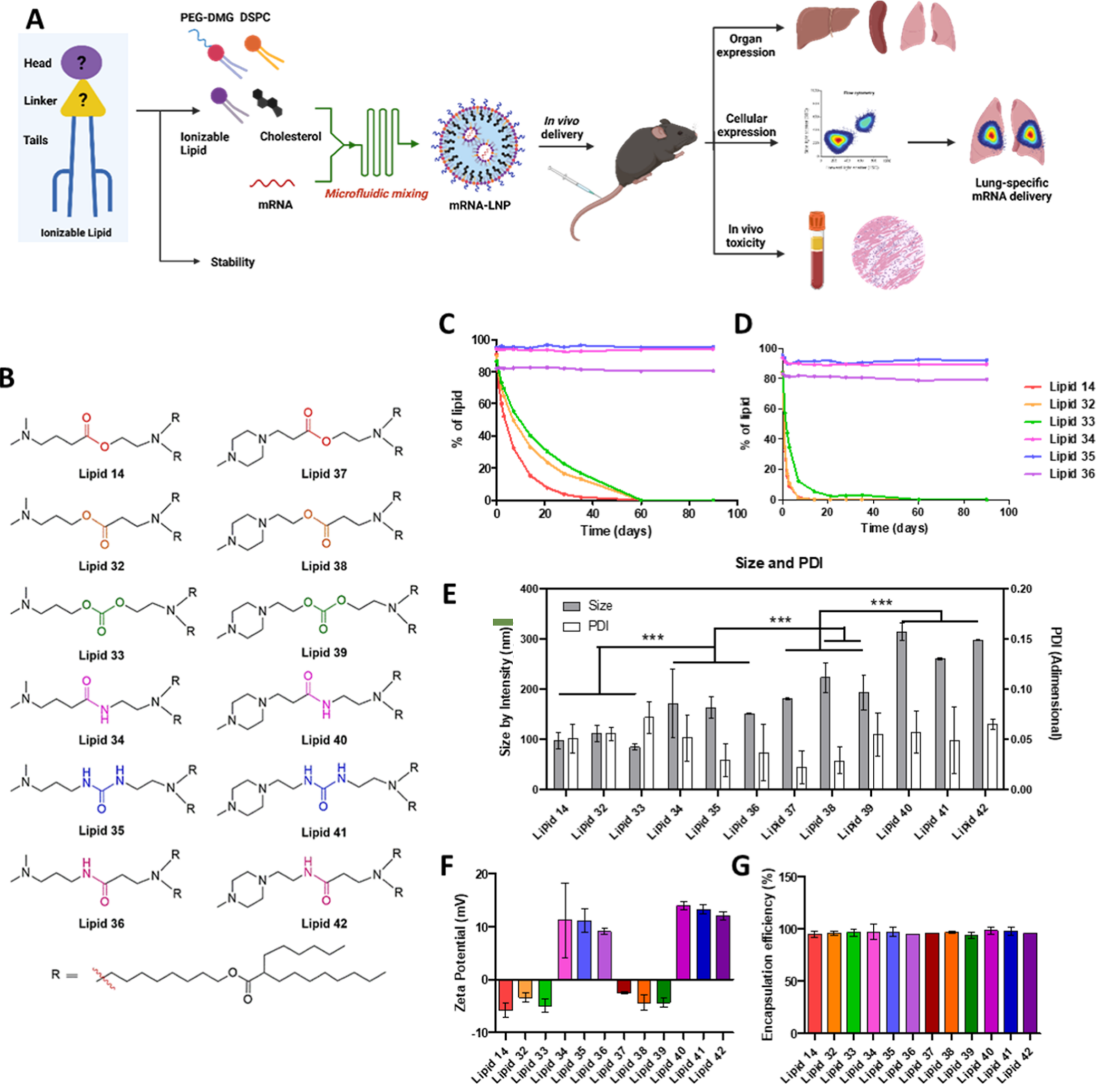
(A) Schematic representation of the workflow of the present study (created with Biorender). (B) Chemical structure of ionizable lipids. Chemical stability of lipids with different linkers dissolved in absolute ethanol (C) and in water: ethanol 1:3 mixture (D) measured by HPLC with a CAD detector. (E) Size and PDI of the LNPs formulated using the different lipids. (F) ζ of the formulated LNP. (G) mRNA encapsulation efficiency measured by a Ribogreen assay for every LNP formulation (*n* = 3 for every group, ***: *p* < 0.001).

However, the clinical success of these transformative therapeutics relies heavily on developing safe, effective, and highly selective delivery systems for targeted mRNA delivery to specific tissues and cell types.^10−12^ LNPs are the most advanced and clinically approved platforms developed to this end.^8,13−16^ Typical LNPs are formulated using ionizable lipids, phospholipids, cholesterol, and polyethylene glycol (PEG)-conjugated lipids.^8,16^ Among these components, ionizable lipids play a critical role in encapsulating, protecting, and transfecting the mRNA cargo into cells. Ionizable lipids are characterized by their environment-responsive behavior. Indeed, they assume a positive charge at acidic pH, a feature that promotes their interactions with negatively charged mRNAs during LNP formation and that can destabilize cellular membranes, facilitating endosomal escape after LNPs undergo cellular internalization. On the other hand, at physiological pH, ionizable lipids retain a neutral charge that is useful to minimize the particle’s interactions with anionic cell membranes, limiting LNP toxicity and immunogenicity, as well as improving their pharmacokinetic properties.^17^ The structure of ionizable lipids typically consists of a hydrophilic headgroup, two hydrophobic tails, and a linker connecting these components (Figure 1A).^14^ In general, the amino/hydroxyl groups have been used as hydrophilic head groups, linoleyl, and branched aliphatic carbon chains have been used as hydrophobic tails, and ester bonds have been often selected as linkers to design ionizable lipids for efficient mRNA delivery.^14^

Despite rapid progress in optimizing LNP formulations, delivering mRNA beyond the liver and to specific cells is still considered a significant challenge as the majority of mRNA-LNPs still accumulate in the liver upon systemic administration. Several efforts have focused on developing mRNA-LNPs using surface-modifying active targeting moieties such as peptides, antibodies (or their fragments), and natural ligands.^18−23^ However, modifying the surface of LNPs by adding targeting moieties can require extensive effort in recombinant protein expression or chemical conjugation and subsequent purification, resulting in a very cumbersome process that is not easily scalable, and could also lead to LNP instability and immunogenicity issues.^15,24^

Another important breakthrough includes the development of organ-selective LNPs for tissue-specific mRNA delivery obtained by modifying the LNPs’ zeta potential (ζ) with charged lipid molecules.^25−27^ This strategy is conceptually simpler but is based on radical changes in the LNPs’ composition that might require additional characterization.

A different approach is based on pretreating the patient with “decoy” nanoparticles to saturate the liver’s clearing capacity; however, this requires the administration of a high amount of decoy particles, leading to safety concerns.^28,29^

Considering the unique accessibility granted by lung anatomy, the use of topical delivery routes such as nebulization or intratracheal instillation has also been employed for direct delivery to the lungs.^30^ However, these approaches require the careful modulation of the LNP composition that would enable them to reach into the deep segments of the respiratory tract and adhere to the alveoli, and the bioavailability of the currently used formulations is still limited.^31^

Other than these strategies, a few reports have shown that the structure of ionizable lipids could lead to relevant changes in the resulting LNP organ and cellular tropism.^32−37^ For instance, piperazine-containing lipids have demonstrated preferential delivery to a wide range of immune cell populations in both the liver and spleen, as well as various cell types in the placenta.^32,33^ Furthermore, we recently reported cell-type-specific mRNA delivery to CD11b^hi^ macrophages using specific ionizable lipids, without any additional targeting moiety.^34^ Another interesting study showed the change of organ selectivity from the liver to the lung by using ester and amide-containing tails.^38^ Recently, multicomponent synthetic approaches have been shown to enable spleen-selective mRNA delivery.^35,37^ These detailed studies highlight how the structure of ionizable lipids plays a significant role in governing the delivery of mRNA to specific organs and cell types. However, predicting the specificity of new ionizable lipids based solely on their chemical structure is still a challenge and requires extensive study, and the impact of different lipid linkers on LNP stability, biodistribution, and mRNA delivery remains poorly understood.^39,40^

To address this knowledge gap, we designed and synthesized a library of 11 ionizable lipids using different biodegradable linkers, including ester, carbonate, amide, and urea moieties (Figure 1B). By extensively screening the LNPs produced with these new lipids, we demonstrated how amide and urea linker-containing lipids resulted in enhanced chemical stability under storage, making them suitable for large-scale production and formulation. Furthermore, we gained insights into the structure–activity relationship of ionizable lipids, showing how the presence of amide and urea groups in the lipid can fine-tune the LNPs’ p*K*_a_, resulting in improved LNP lung tropism. Finally, we showed how LNPs formulated with lung-targeting lipids improve the therapeutic efficacy of mRNA encoding for the pseudomonas exotoxin A (mPE-A) in reducing lung tumor metastasis progression compared to particles containing the commercially available SM-102 lipid, resulting in improved survival.

This work provides innovative insights that are useful for the future design of organ-specific LNP formulations to achieve selective mRNA delivery.

## Results and Discussion

### Ionizable Lipid Design, Characterization, and Stability

Our primary objective was to design and synthesize new biocompatible ionizable lipids for extrahepatic delivery of mRNA. Over the past few years, we have developed several ionizable lipids for various RNA-based therapeutic and vaccine applications.^34,41−48^ Lipid 14 stood out as one of the top-performing lipids in our library, with its ester-branched aliphatic tails enabling the development of COVID-19 and bacterial vaccines.^46−48^ However, lipid 14 could be susceptible to hydrolytic degradation in ethanol and aqueous environments due to its ester linker, which hinders its utility following long-term storage. Thus, the use of different linkers could provide more stability to the structure of the ionizable lipid. Therefore, keeping lipid 14 with its backbone, we designed a combinatorial library of ionizable lipids using different biodegradable linkers (Figure 1B). In particular, for lipid 14 and lipid 34, an ester linker was used, whereas a reverse-ester linker was employed for lipid 32 and lipid 38. In the case of lipid 33 and lipid 39, a carbonate linker was selected, while an amide linker was used for lipid 34 and lipid 40. For lipid 35 and lipid 41, a urea linker was employed, whereas a reverse-amide linker was used for lipid 36 and lipid 42. All lipids were synthesized using standard organic synthesis procedures and underwent characterization by NMR and mass spectroscopic techniques (see the Supporting Information).

Upon synthesizing the lipids, we investigated their stability in different solvents. To this end, we dissolved the lipids in either ethanol or a water/ethanol mixture (1:3) and monitored their stability for 3 months using a high-performance liquid chromatography (HPLC) device equipped with a charged aerosol detector (CAD). We observed that while the amide (lipid 34), urea (lipid 35), and reverse-amide (lipid 36) linkers were stable and did not undergo hydrolysis in both solvent systems, the ester (lipid 14), reverse-ester (lipid 32), and carbonate (lipid 33) linkers rapidly degraded in the water–ethanol mixture as compared ethanol (Figure 1C,D, respectively).

### LNP Preparation and Characterization

LNPs’ composition was optimized by performing a small design of experiment (DoE)-based screening. Briefly, different lipid critical process parameters (CPPs) and critical quality attributes (CQAs) were used to generate a custom design (Figure S1A) containing 20 formulations. After formulating and characterizing these LNPs, statistical analysis elucidated how the CPPs influencing LNPs’ features were the different lipid proportions as well as the ionizable lipids’ identity (Figure S1B). We used these CPPs to interpolate a mathematical model correlating them to CQAs and simulated 10,000 theoretical formulations using a Monte Carlo simulation. Among these LNPs, a series of thresholds were applied to select the formulations with the highest theoretical performance (top 10%). As shown in Figure S1C, the best-performing theoretical formulations presented a very similar composition, with the average composition being ionizable lipid, cholesterol, DSPC, and PEG-DMG in molar ratios of 40:48.5:10:1.5, respectively. Thus, LNPs were formulated with these molar ratios using the synthesized lipids. As model cargo, firefly luciferase mRNA (mLuc) was encapsulated using the NanoAssemblr microfluidic mixing device, as described in the Experimental Section.

The p*K*_a_ of LNPs formulated with different lipids was also estimated by using a 6-(*p*-toluidino)-2-naphthalenesulfonic acid (TNS)-based fluorometric assay. Most of the new lipids had a p*K*_a_ between 5.9 and 6.5, which is considered suitable for most ionizable lipid-based LNP formulations (Figure S2).^49^ Interestingly, LNPs formulated with lipids containing either urea or reverse-amide linkers (lipids 35, 36, 41, and 42) had a p*K*_a_ close to or higher than 7, suggesting that the number and orientation of the amide linkers can influence the lipid environmental response.

Noticeable variations were observed in the hydrodynamic diameter and zeta potential (ζ) of the produced LNPs (Figure 1E,F). The *N*-methylpiperazine head-containing lipids (lipid 37–lipid 42) yielded higher-sized LNPs as compared to dimethylamine head lipids (lipid 14 and lipid 32–lipid 36). Also, LNPs formulated with ester, reverse-ester, and carbonate linker-containing lipids (lipid 14, lipid 32, lipid 33, and lipid 37–39) had a negative ζ, whereas LNPs consisting of amide, urea, and reverse-amide-containing lipids had a positive ζ (lipid 34–lipid 36 and lipid 40–lipid 42). The polydispersity index (PDI) was less than 0.1 for all LNPs, making the formulations monodisperse. The encapsulation efficiency was >95% for all the LNP formulations (Figure 1G), and mRNA encapsulation as well as retention of its integrity was further confirmed by agarose gel electrophoresis (Figure S3).

### In Vitro LNP Transfection Efficiency

**Figure 2:**
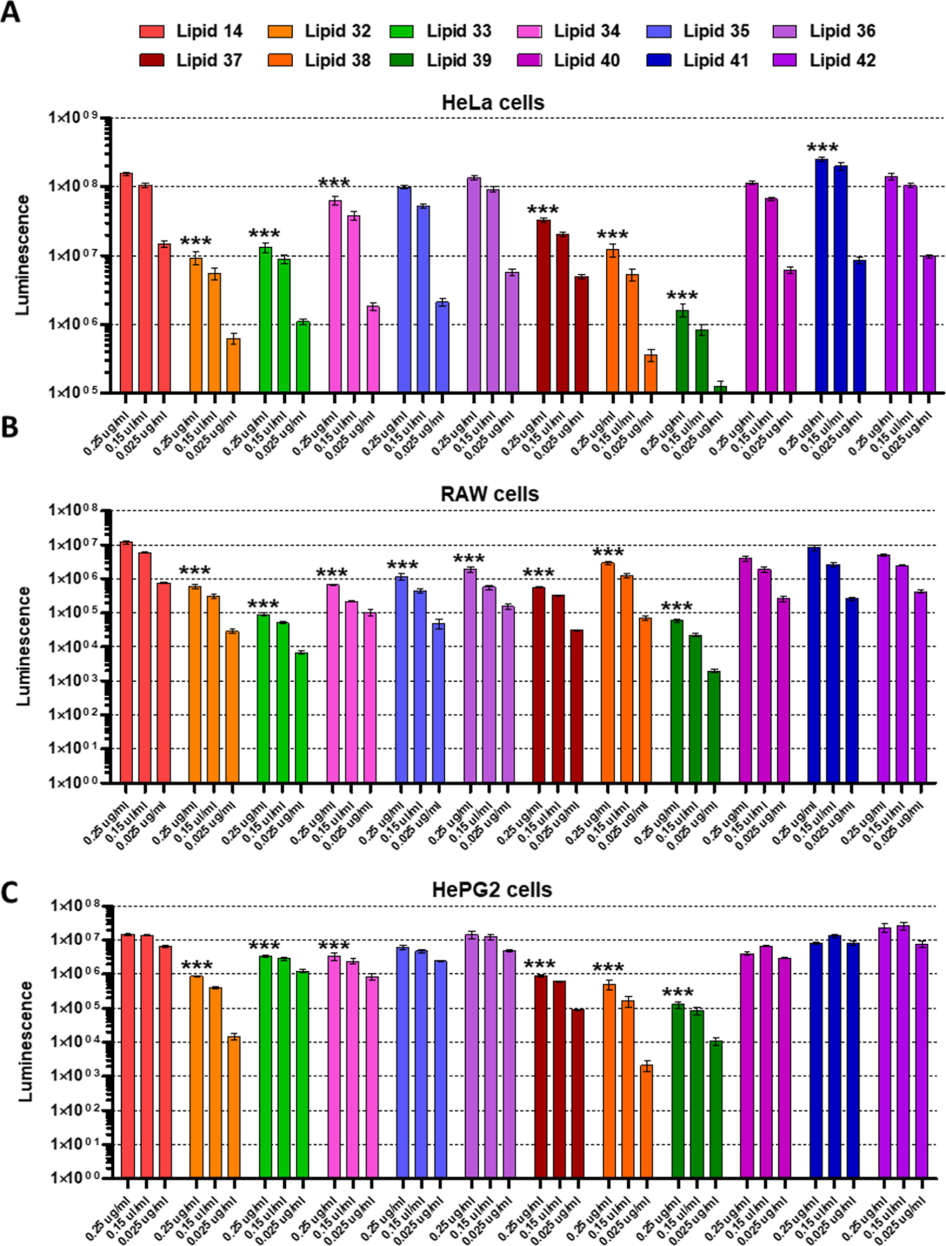
In vitro assessment of Luc expression in HeLa (A), Raw264.7 (B), and HepG2 (C) cells. Every measurement was performed in triplicate (*n* = 3 for every group, ***: *p* < 0.001 compared to the 0.25 μg/mL treatment with lipid 14).

Upon confirming the LNPs’ physio-chemical properties, we screened the formulations’ transfection efficiency in HeLa, RAW 264.7, and HepG2 cell lines by encapsulating mLuc as model cargo (Figure 2). HepG2 and RAW 264.7 cells were selected to represent the liver and the mononuclear phagocytic system as compartments that are often targeted by nanoparticles after systemic administration. In addition, HeLa cells were selected as a more general, fibroblast-like cell line.

Looking at individual lipids, in HeLa cells (Figure 2A), all of the LNPs formulated with amide or urea linkers were the best-performing ones. Conversely, in RAW 264.7 cells (Figure 2B), the best-performing LNPs were the ones formulated with lipid 36, lipid 38, and lipids 40–42, while HepG2 cells (Figure 2C) were transfected best by lipids 33–36 and lipids 40–42.

However, to elucidate the structure–activity relationship of the new ionizable lipids, it is important to account for how these molecules can influence the LNP features, which in turn can change the particles’ biological behavior, constituting noise factors. Thus, accounting for the effect of ionizable lipids on particles’ properties such as size, apparent p*K*_a_, and ζ would help us to discern the direct effect of these molecules on the LNP activity.

Since it is technically challenging to control for these variables in an experimental setting, we assessed the possible correlations between LNP size, ζ, and Luc signal from the tested cell lines, demonstrating how there was no correlation between the LNP size and their Luc signal in vitro (Pearson’s correlation coefficient (PCC) < 0.4 for all cell lines, Figure S4A).

However, the data demonstrated a positive correlation between the ζ and the Luc signal in HeLa and HepG2 cells (PCC = 0.69 and 0.65; *p* = 0.0132 and 0.0220, respectively) with positively charged LNPs (lipids 34, 35, 36, 40, 41, 42) showing slightly higher transfection efficacy (Figure S4B). These observations are reinforced by a similar positive correlation between these two cell lines and the LNPs’ p*K*_a_ (PCC = 0.74 and 0.72 and *p* = 0.0064 and 0.0079, respectively, Figure S4C). This can be explained by the higher electrostatic interaction of positive LNPs with the cellular surface, leading to higher uptake.^51^ Interestingly, all the lipids with *N*-methyl-piperazine groups yielded particles with slightly higher transfection efficiency, although the difference was not significant compared to the dimethylamine head lipids (Figure S4D–F).

Overall, these in vitro studies allow us to elucidate some fundamental interactions between LNPs and different cell lines, providing an inkling for further in vivo studies.

### In Vivo Screening of LNPs for mRNA Delivery

**Figure 3:**
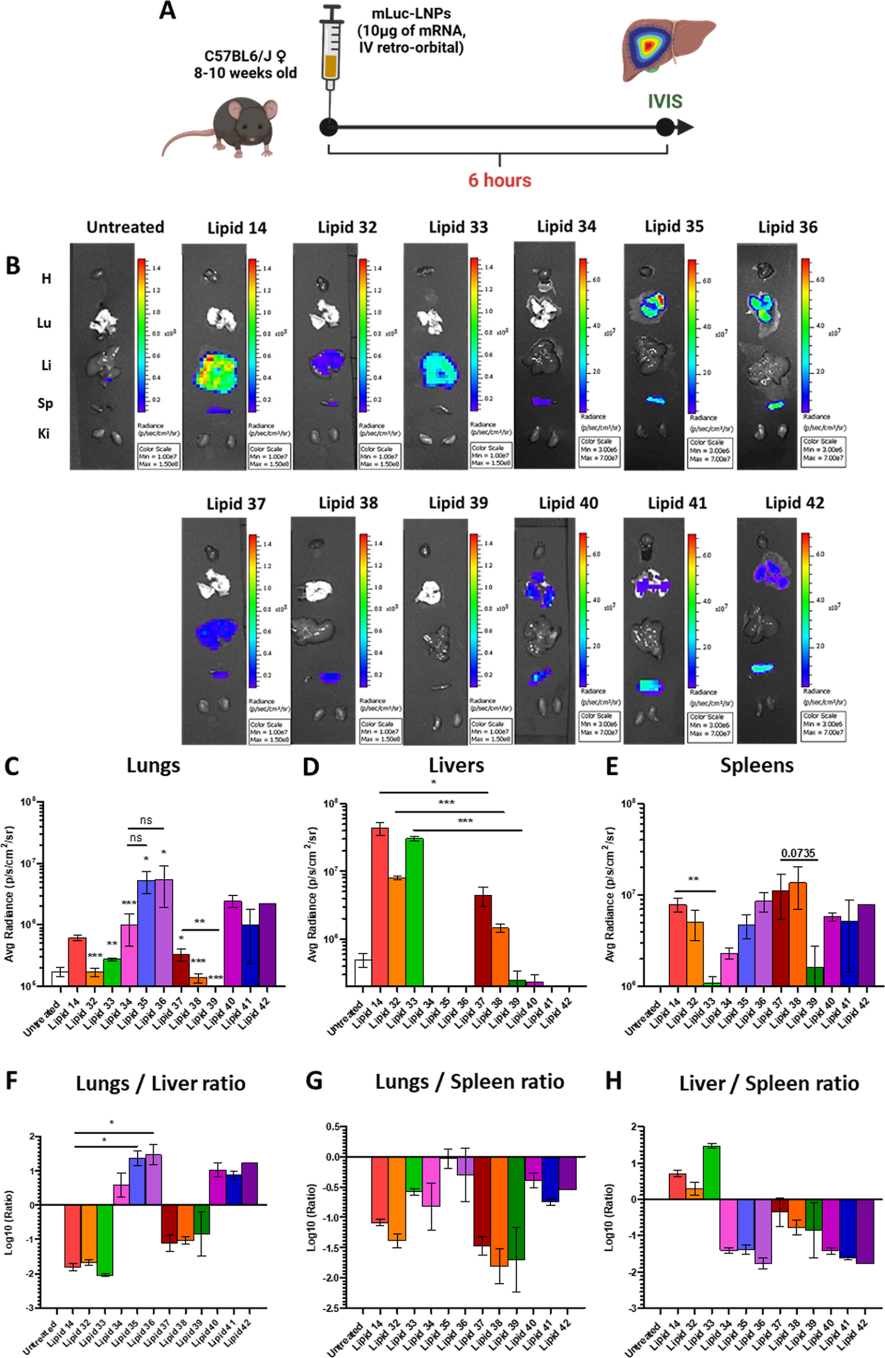
(A) Summary of the administration route and timing used for the Luc expression study (created with Biorender). (B) Representative images of Luciferase signal detected in mice hearts (H). Lungs (Lu). Livers (Li). Spleens (Sp). Kidneys (*K*_i_). Quantification of the average radiance measured in the lungs (C), livers (D), and spleens (E) and the relative ratios between Luc signals among different organs (F–H). Four mice were included in every experimental group (*: *p* > 0.05, **: *p* < 0.01, ***: *p* < 0.001).

To test these ionizable lipids in vivo, C57BL/6J mice were systemically injected with a volume of particles corresponding to 10 μg of mLuc per animal via retro-orbital injection. The luminescence signal in major organs was assessed after 6 h (Figure 3A). Once again, to account for the presence of possible correlations between LNP features and organ biodistribution, we performed the same correlation analysis discussed in the previous section.

Notably, the size of LNPs was reversely correlated with the liver Luc signal, underlining how smaller particles tended to accumulate more in this tissue (PCC = −0.6741, *p* = 0.0162, Figure S5A). Conversely, LNPs with positive ζ (lipids 34–36 and lipids 40–42) showed higher luminescence in the lung (PCC = 0.6345, *p* = 0.0267) and lower luminescence in the liver (PCC = −0.5815, *p* = 0.0474) and kidneys (PCC = −0.7378, *p* = 0.0062, Figure S5B). Furthermore, the apparent TNS-measured LNPs’ p*K*_a_ values had a strong positive correlation with the lungs’ expression (PCC = 0.8417, *p* = 0.0006) and a negative correlation with the kidneys’ signal (PCC = −0.6978, *p* = 0.0116, Figure S5C).

When looking at the possible correlations between in vitro and in vivo results, it is interesting to note how the Luc signal in RAW 264.7 cells and the one detected in the animal spleens were positively correlated with each other (PCC = 0.7746, *p* = 0.0031, Figure S5D). From a biological standpoint, this observation could be attributed to the presence of phagocytic cells in the spleen that have somewhat similar behavior to the RAW 264.7 macrophage-like cells. It is also important to note how both the behaviors of HeLa and HepG2 hepatocyte cells were positively correlated with the lung signal (PCC = 0.8843 and 0.8428 and *p* = 0.0006 and *p* = 0.0001, respectively, Figure S5E,F). Ultimately, these correlations can be easily explained by the LNP positive ζ that increases both cellular expression and lung expression.

Despite these correlations, the positive ζ LNPs formulated using ionizable lipids with amide and urea linkers behaved heterogeneously for both head groups. Lipids 40–42 were not well tolerated by the animals, and some of them displayed obvious behavioral signs of discomfort and had to be sacrificed soon after injection for ethical reasons. This acute adverse reaction could be attributed to the combination of larger LNP size (above 200 nm) derived from the *N*-methylpiperazine headgroup and positive ζ, which could have resulted in LNP aggregation after injection, causing embolism in the mice. Thus, lipids 40–42 were excluded from further studies. The other positively charged ionizable lipids (lipids 34–36) were well tolerated by animals and showed high lung specificity, with lipids 35 and 36 showing both a slightly higher lung signal (Figure 3C) and high lung/liver and lung/spleen ratios when compared with ester, reverse-ester, and carbonate linkers (Figure 3F,G). Since the particles formulated with these three lipids had analogous size and surface charge, the difference between lipid 34 and lipids 35 and 36 can be strictly attributed to the lipid structures themselves. Indeed, the presence of amide groups in the lipid linker resulted in increased LNPs ζ and, therefore, lung transfection. However, lung tropism is further improved by either positioning this nitrogen closer to the dimethyl-amine head (lipids 36) or having nitrogen on both sides of the linker (lipid 35), suggesting the nuanced effect of even minor structural changes on LNP tropism. Thus, lipids 35 and 36 were selected as lead lipids for delivery of mRNA to the lungs.

Lipid 33, despite showing a similar Luc signal in the liver (Figure 3D) to lipid 14, displayed much lower signals in other organs, especially the lungs and spleen, resulting in a higher specificity in the liver, as highlighted by the low lungs/liver and the high liver/spleen ratios (Figure 3F,H). Comparing the behavior of lipid 33 to lipids 14, 32, and 37–39, for each of these lipids with dimethylamine heads, their *N*-methyl-piperazine equivalent always resulted in a lower liver signal (Figure 3D). This corroborates the effect of LNP size on liver biodistribution since all the piperazine lipids yielded bigger particles than their counterparts. Similarly, lipid 38 displayed higher spleen specificity in the form of a slightly better spleen luminescence (Figure 3E) and Lung/spleen ratio (Figure 3G). Thus, lipids 33 and 38 were selected, respectively, as lead lipids for the liver and spleen.

It is especially notable to observe how the combination of a carbonate linker and an *N*-methylpiperazine head in lipid 39 seems to abolish lipid activity in all the tested cell lines and organs, making it the worst-performing lipid. The mechanism behind this could warrant further investigation and the study of additional organs.

Finally, the heart and kidney signal measured was barely detectable compared to the background radiance, and no clear trend was visible among ionizable lipids (Figure S6A,B, respectively).

As presented in Figure S7, in which the different lipids are arranged in a ternary plot based on their different proportions of organ radiance, also allows us to evidence some clear trends: all the lipids with ester, reverse-ester, or carbonate linkers are mostly clustered between the liver and spleen, with the dimethylamine lipids among these being the ones oriented toward the liver while the *N*-methyl-piperazine clustered toward the spleen. All the lipids having amide, reverse-amide, or urea linkers were distributed between the lungs and spleen, with the *N*-methyl-piperazine lipid accumulation proportionally higher in the spleen compared to the dimethylamine lipids. Furthermore, this graphical summary helps us observe how lipid 33, lipids 35–36, and lipid 38 have the highest proportional signal for the liver, lungs, and spleen, respectively, since they are the ones closer to the vertex of each organ, representing higher luminescence signal. These data make them the most selective lipids in our library for these tissues, confirming them as lead compounds to deliver mRNA toward them for further investigation.

Taken together, these results show how small differences in the linker structure of ionizable lipids can have radical effects on the resulting particles’ transfection and tropism in different organs.

### LNPs’ Cellular Transfection Profile and Biodistribution In Vivo

**Figure 4:**
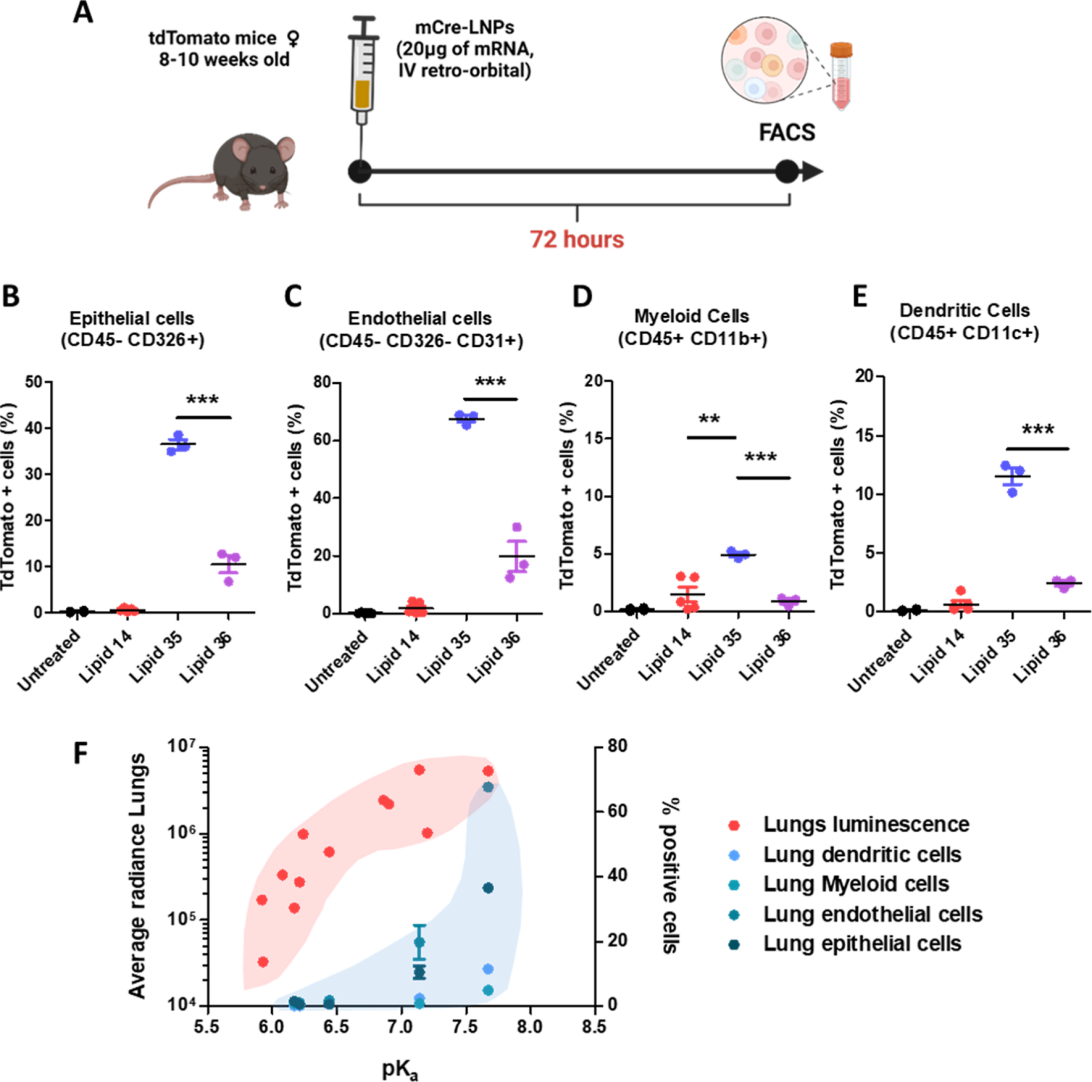
(A) Summary of the experimental setup used to assess cellular transfection in vivo (created with Biorender). Flow cytometric quantification of different lung cell populations in TdTomato mice for epithelial cells (B), endothelial cells (C), myeloid cells (D), and dendritic cells (E) (*n* = 3 mice/group). (F) Assessment of the general trends across the correlations between the p*K*_a_ values of LNPs and the lung luminescence signal and the percentage of transfected cells observed in the lungs of TdTomato mice (3 mice were included in each group, **: *p* < 0.01, ***: *p* < 0.001).

We next investigated the cellular specificity of LNPs’ transfection formulated with the selected leads lipid 35 and lipid 36. To elucidate this, we formulated LNPs with Cre recombinase mRNA (mCre) and injected them in Ai9-tdTomato mice as previously described (Figure S8).^5,43^ As can be seen in Figure S9A,B, the mCre-LNPs displayed analogous size distribution and mRNA encapsulation compared to their mLuc-loaded equivalents. We quantified the functional mRNA delivery as a percentage of tdTomato^+^ cells in different organs 72 h post-systemic administration (Figure 4A). Using flow cytometry, we quantified successful mRNA transfection and expression in different cell types, including myeloid immune cells (CD45^+^ CD326^–^ CD11b^+^), dendritic cells (CD45^+^ CD326^–^ CD11c^+^), endothelial cells (CD45^–^ CD11b^–^ CD326^–^ CD31^+^), and epithelial cells (CD45^–^ CD326^+^).

In concurrence with the in vivo mLuc expression data, the lungs of mice treated with lipids 35 and 36 resulted in significantly more tdTomato^+^ cells than the lungs of mice injected with lipids 14, 33, and 38. Comparing lipids 35 and 36 for their transfection efficiency in different lung cells, we observed that lipid 35 efficiently reached nonimmune cells, with >60% of endothelial cells and ∼40% of epithelial cells expressing tdTomato (Figure 4B,C). Albeit to a lesser extent, this lipid also showed higher transfection levels in myeloid cells and dendritic cells (Figure 4D,E). It should be noted that despite lipids 35 and 36 having similar physicochemical properties and mLuc expression profiles, lipid 35 displayed a significantly higher transfection efficiency in all the analyzed lung cell populations. The lung specificity of lipids 35 and 36 is further confirmed by the complete absence of tdTomato^+^ cells in the spleen and the liver (Figure S10E–M) of mice injected with these LNPs. Therefore, we decided to focus our efforts on further assessment of lipids 35 and 36 as lead lipids for mRNA delivery to the lungs. However, lipids 33 and 38 did not confirm their liver and spleen selectively in this model (Figure S10).

When comparing the mLuc results with the Cre-tdTomato murine models, lipids 35 and 36 strongly validated the previous mLuc results by showing efficient transfection in all of the lung cell populations analyzed, with lipid 35 showing a remarkably higher transfection compared to all other formulations and across all of the lung cells.

When focusing on the lead formulations, LNPs with higher p*K*_a_ resulted in LNPs with higher ζ, which in turn appeared to have higher lung tropism. Indeed, the lung transfection observed in vivo mLuc expression starts to significantly increase only for LNPs with p*K*_a_ values above 6.5 and seems to reach a plateau for mLuc transfection of the lungs at a p*K*_a_ of 7.2 (lipid 36, Figure 4E). However, when comparing the mLuc results with tdTomato expression, the percentage of positive cells steeply increased among cell populations for LNPs containing lipid 35 (p*K*_a_ = 7.6), compared to ones formulated with lipid 36. Thus, there seems to be a threshold value for the p*K*_a_ above which the transfection efficiency in lung cells significantly improved as reported previously.^52^

We also analyzed the biodistribution of LNPs loaded with fluorescently labeled RNA, assessing the possible correlations between the transfection efficiency observed in the tdTomato model and simple particle accumulation. In this instance, we investigated lipid 14 as a benchmark and lipids 35 and 36 as our main leads for lung targeting. As summarized in Figure S11A–D, lipids 35 and 36 accumulated more than lipid 14 in all the lung cell populations analyzed but with a percentage of positive cells that was significantly lower compared to the tdTomato model. Furthermore, unlike the lung data in Figure 4, there was no clear difference between lipids 35 and 36. Thus, lipid 35 and lipid 36 particles had similar lung targeting capacity. However, lipid 35 displayed higher transfection efficiency in tdTomato animals, suggesting a possible alternative mechanism behind lipid 35 specificity that is not strictly dependent on particle accumulation. When considering instead the LNP biodistribution in the liver (Figure S11E–H) and spleen (Figure S11I–M), we observed an overall similar accumulation for all three lipids, with lipids 35 and 36 accumulating slightly more in the liver endothelial cells, and at the same time significantly less in the spleen B-cells. Despite these small differences, the different ionizable lipids yielded LNPs with overall similar biodistribution, in contrast with the data in Figure S10. This reinforces the possible contribution of a different, perhaps intracellular, mechanism behind the observed transfection selectivity. Furthermore, the comparable liver deposition allows us to exclude first-pass lung accumulation as the mechanism behind transfection in these organs.

Taken together, these results underline how minor changes in the structure of ionizable lipids can radically affect the resulting LNP organ transfection. In particular, the addition of nitrogen atoms to the lipid linker enables the fine-tuning of the LNPs p*K*_a_, which can be leveraged to achieve not only high levels of protein expressions selectively in the lungs without using additional targeting moieties but also a more widespread transfection across all the major lung cell populations. Based on these results, we selected lipid 35 as the definitive lead for lung targeting.

### Safety Profile of Lipid 35

**Figure 5:**
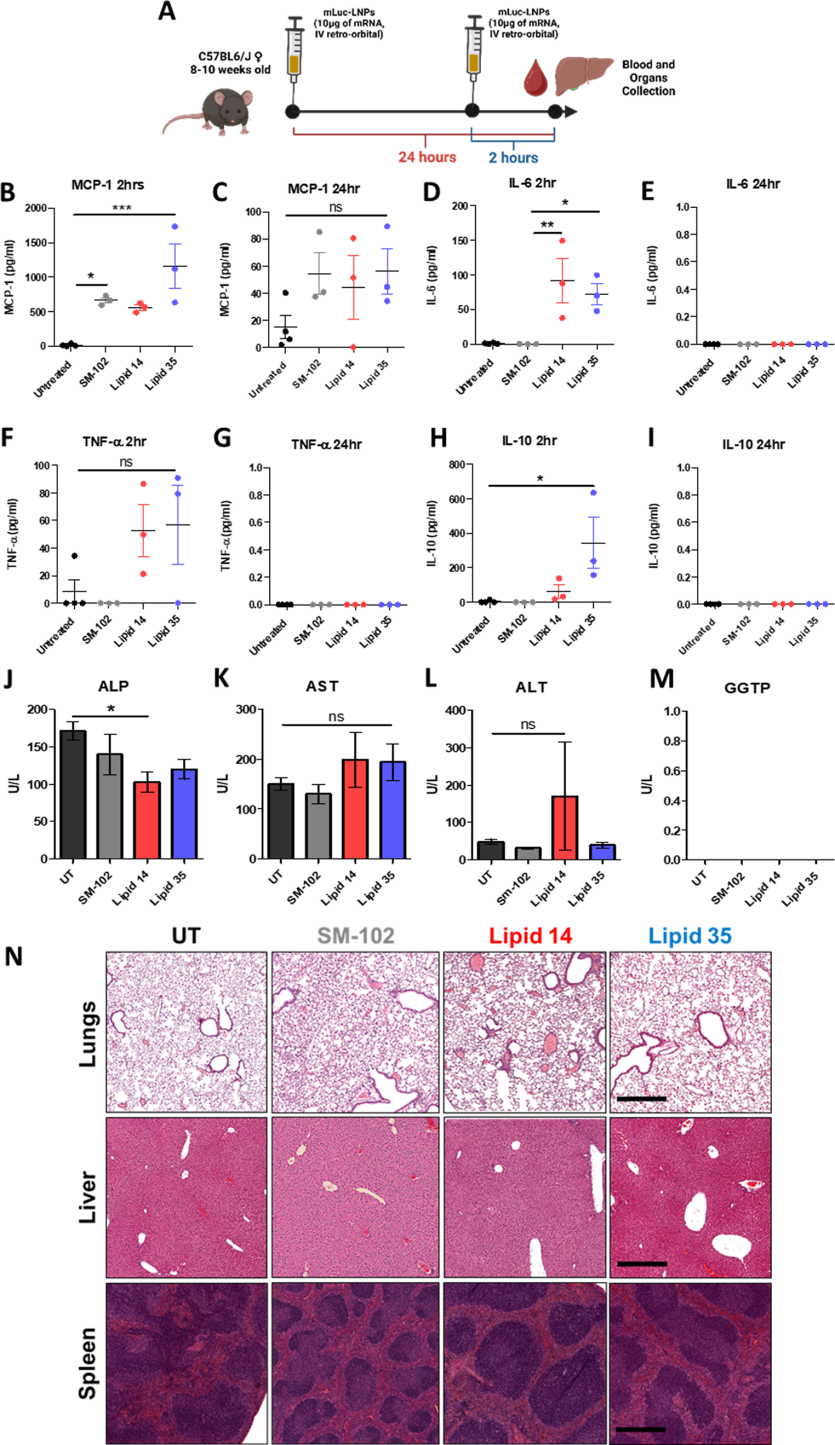
(A) Summarized experimental design used for the assessment of LNPs’ biocompatibility (created with Biorender). ELISA-based assessment of the plasmatic levels of MCP-1 (B,C), IL-6 (D,E), TNF-α (F,G), and IL-10 (H,I). Quantification of the plasma levels of ALP (J), AST (K), ALT (L), and GGTP (M) after 24 h from a single LNP IV injection. (N) Representative hematoxylin/eosin-stained sections of paraffin-included tissues harvested 24 h from LNP injection (5× magnification, scale bar was set at 500 μm). Three mice were included in each experimental group (*: *p* < 0.05).

Focusing on the best-performing lipid 35, we extensively tested its biocompatibility after a single bolus intravenous injection. To this end, a volume of LNPs equivalent to 10 μg of mRNA was injected through the retro-orbital vein. To assess a possible acute immune response to LNP injection, mice blood was collected at 2 and 24 h after injection and the concentrations of Monocyte Chemoattractant Protein-1 (MCP-1), interleukin 6 (IL-6), tumor necrosis factor α (TNF-α), and interleukin 10 (IL-10) in mice plasma were measured by ELISA (Figure 5A). As a control, we selected the clinically approved SM-102 ionizable lipid.

At 2 h postinjection, all cytokines were slightly elevated in the serum. Pro-inflammatory MCP-1, IL-6, and TNF-α were similarly elevated compared to the control SM-102 (Figure 5B,D,F). The elevation was comparable between lipid 14 and lipid 35 and resulted in significantly higher levels of IL-6 compared to SM-102 but not for any other cytokine. Conversely, the immunomodulatory cytokine IL-10 was only slightly induced by lipid 35 (Figure 5H). Nevertheless, the increase in serum cytokines was transient as all cytokines dropped to the level of untreated animals within 24 h postinjection, with only a very small amount of MCP-1 detectable (Figure 5C,E,G,I).

After 24 h of injection, we also measured the concentration of key serum markers, including alkaline phosphatase (ALP) and liver transaminases: serum aspartate aminotransferase (AST) and alanine transaminase (ALT). All markers were comparable between the untreated mice, SM-102, and lipid 35-treated animals. AST and ALT were slightly not significantly increased by lipid 14 (Figure 5J–M).

Finally, we investigated whether any tissue damage is caused by the different lipids in the lungs, the livers, and the spleens of injected animals. To this end, we harvested these organs 24 h post-systemic administration and performed histological assessment using hematoxylin/eosin-stained sections. As shown in Figure 5N, no visible sign of tissue inflammation, infiltration of leukocytes, necrosis, or fibrosis was detectable in any treatment group.

Taken together, these results highlight that lipid 35 after bolus injection is well tolerated with no liver or other major organ damage detected, as confirmed by blood markers and histological analysis.

### Assessment of Lipid 35-LNPs as an mRNA Carrier in a Murine Model of Metastatic Lung Cancer

**Figure 6:**
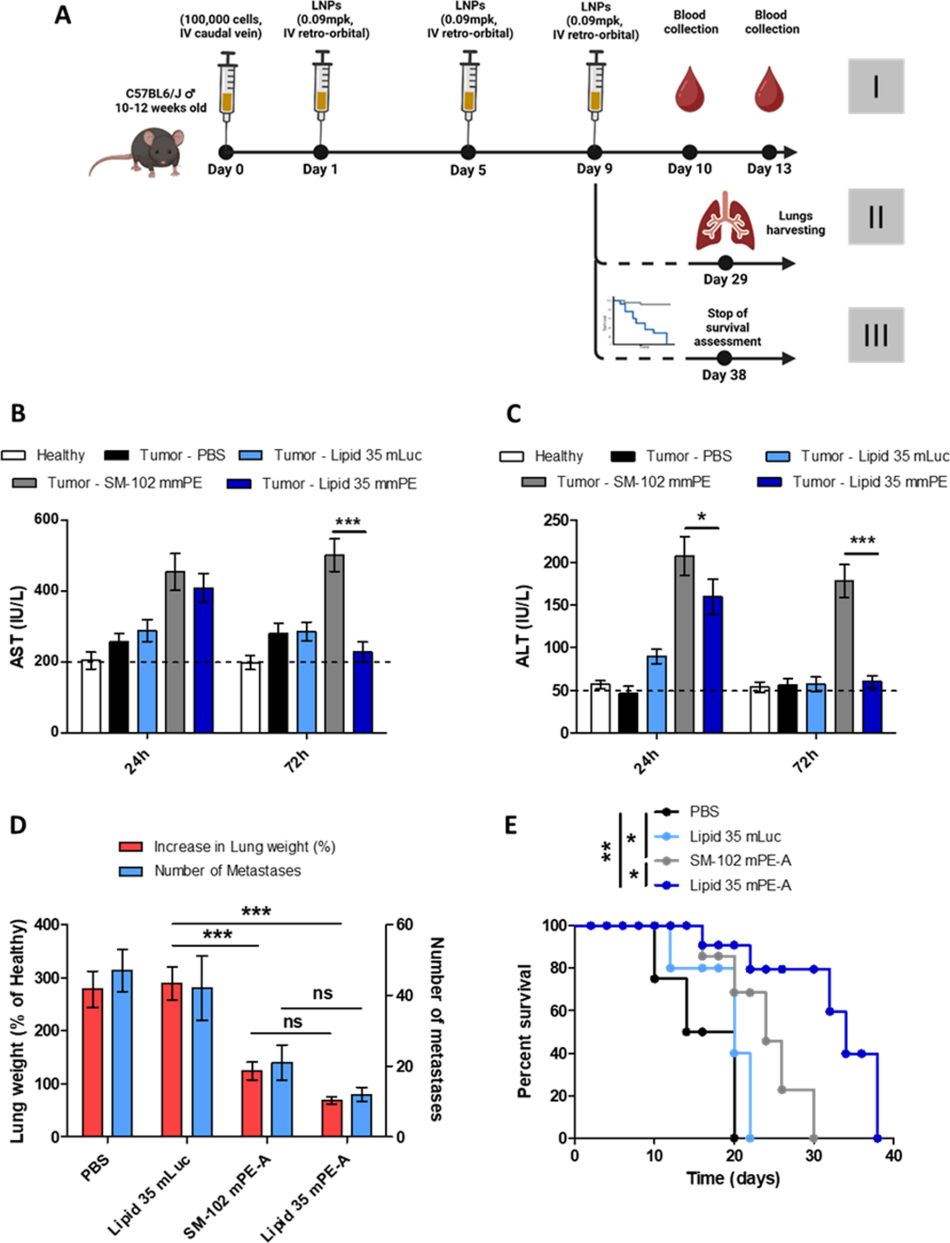
(A) Summary of the experimental design used to assess the LNPs’ biocompatibility (I), reduction of lung metastases (II), and survival in tumor-bearing mice (III) (created with Biorender). Measurement of the plasma levels of AST (B) and ALT (C) in tumor-bearing mice after treatment with different LNPs. (D) Assessment of the increase in lung weight and number of metastases after treatment. (E) Survival percentage of metastasis-bearing mice after LNP treatment (*n* = 7 mice/group; *: *p* < 0.05, **: *p* < 0.01, ***: *p* < 0.001).

The therapeutic potential of LNPs formulated with lipid 35 was tested in a murine model of metastatic lung cancer. To this end, C57BL/6J mice were intravenously injected with B16F10.9 melanoma cells (as detailed in the Experimental Section in Figure 6A). LNPs loaded with mRNA encoding for domain III of the pseudomonas exotoxin A domain (mmPE), a toxic subunit produced by the bacteria *Pseudomonas aeruginosa* were systemically administrated (as detailed in the Experimental section).^53^ As a control group for these experiments, we used lipid SM-102 as a commercial benchmark ionizable lipid formulated with mmPE.

We first assessed the mmPE-A-loaded LNPs’ (mPE-A LNP) tolerability after systemic administration. Interestingly, the treatment with SM-102 mPE-A LNPs caused an increase in the serum levels of AST, ALT, and ALP 24 h of injection (Figures 6B,C and S12A) that remained elevated at 72h, possibly because of accumulation of particles in the liver. However, enzyme levels in lipid 35 mPE-A LNP-treated individuals, despite being elevated in the first 24 h, went down to baseline levels after 72 h from treatment, and ALP levels were not affected by lipid 35. Similarly, blood chemistry 72 h after injection (Figure S12B–E) displayed a slight increase of total protein and urea levels only for SM-102 mPE-A LNP. This could be attributed to the increased lung specificity of lipid 35, which reduces its off-target accumulation compared to the benchmark and results in improved tolerance of the treatment.

Next, the therapeutic efficacy of the LNPs was assessed. As shown in Figure 5C, after sacrifice, tumor-bearing animals treated with lipid 35 LNPs loaded with the mock mLuc mRNA did not show any decrease in lung weight or number of metastases. However, treatment with mmPE-loaded LNPs did result in a decrease of lung weight for both SM-102 (124 ± 17%) and lipid 35, which was slightly, albeit not significantly, more effective than the benchmark (69 ± 7%). Similarly, the number of metastases was decreased further by lipid 35 (12 ± 2) than SM-102 (21 ± 5) compared to both untreated (47 ± 6) and mLuc LNP-treated mice (42 ± 9), albeit the difference was not statistically significant.

When animal survival was assessed after treatment (Figure 6D), both SM-102 and lipid 35 mmPE LNPs displayed a protective effect. However, animals injected with lipid 35 mm PE LNPs had a remarkably higher median survival of 34 days compared to the 24 days of those injected with SM-102 mmPE-LNPs.

Taken together, these results show how the use of lung-targeting lipid 35 can be translated to a significant improvement in the therapeutic profile of LNPs.

### LNP Stability under Storage

**Figure 7:**
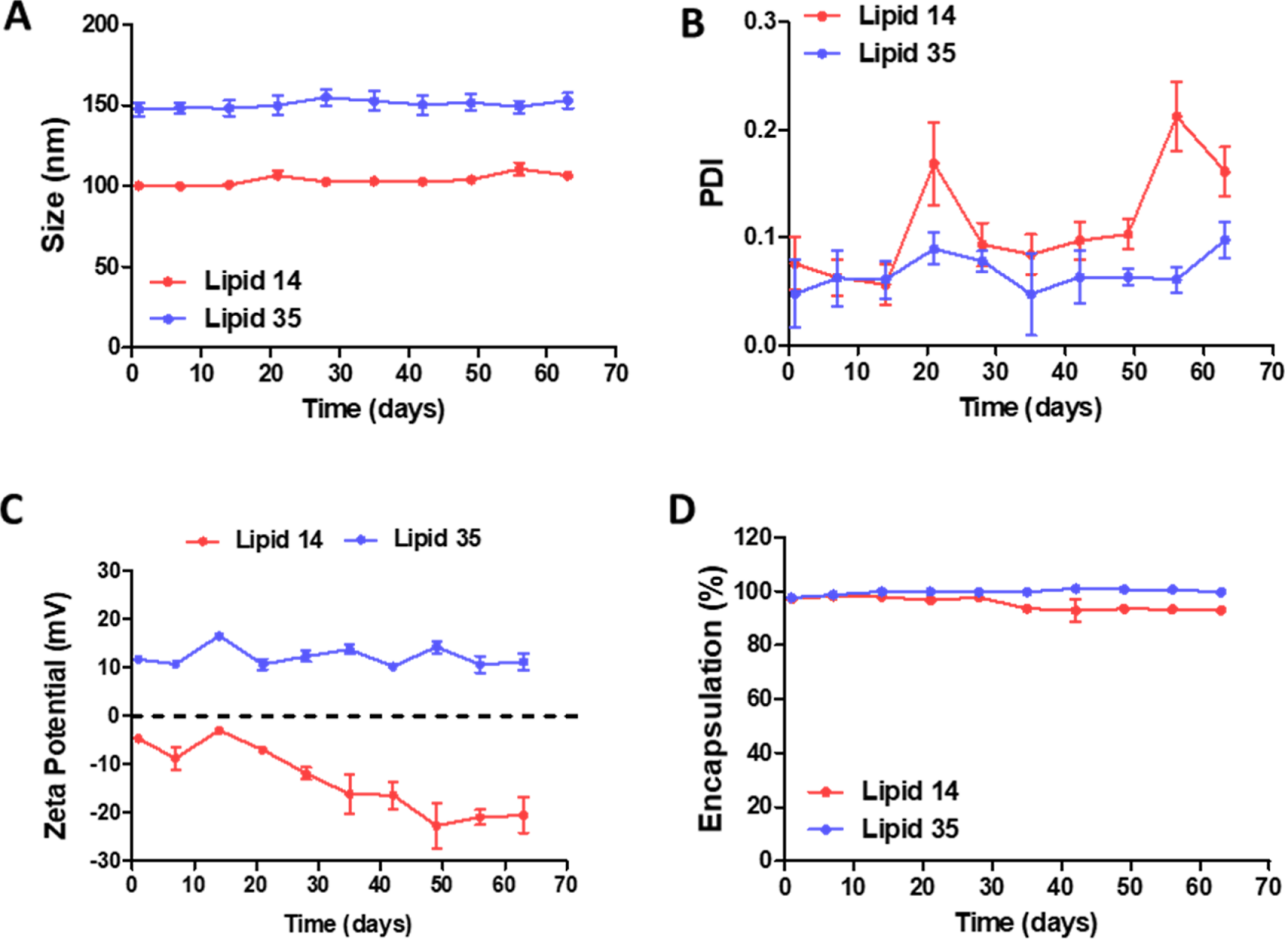
Assessment of the LNP size (A), PDI (B), zeta potential (C), and encapsulation efficiency (D) under storage at 4 °C in 1× PBS (*n* = 3).

To determine whether lipid 35 provided LNPs with improved colloidal stability and reliable transfection efficiency, we tested LNPs when stored in mild conditions and dispersed in 1× PBS at 4 °C. As summarized in Figure 7A, all of the tested LNPs retained their average size distribution over 2 months of storage. The PDI of lipid 14 gradually increased over time, while lipid 35 showed only a small increase in PDI (around 0.1) on day 63 (Figure 7B). Thus, positively charged lipid 35 provided a high ζ, preventing LNP aggregation by electrostatic repulsion. The ζ of the LNPs formulated with lipid 14 decreased over time, going from an average of −6 mV to almost −20 mV at day 63 (Figure 7C). This phenomenon could be attributed to the aggregation of LNP. Of note, there were no changes in the mRNA encapsulation efficiency in both tested formulations (Figure 7D).

In summary, these results indicate that using lipid 35 provides the resulting LNPs with colloidal stability. Combined with the longer chemical stability discussed above, this lipid appears very suitable to produce a stable LNP.

## Conclusions

In the present work, we performed a combinatorial screening on ionizable lipids with different biodegradable linkers with the aim of formulating LNPs for organ-specific mRNA delivery. To the best of our knowledge, most studies on ionizable lipid design so far have focused on the comparison of different head groups or on using hydrophobic lipid tails with different structures, overlooking the linkers as a potential space for ionizable lipid optimization.

After ionizable lipids with different biodegradable linkers were synthesized, their chemical stability was assessed in ethanol or a water and ethanol mixture, showing how amide, reverse-amide, and urea linkers provided these molecules with increased stability.

Subsequently, LNPs were formulated using the new lipids and were extensively characterized, highlighting how the structure of ionizable lipids could have a stark effect on LNP features. Specifically, when introducing *N*-methyl-piperazine head groups, the particles displayed larger size compared to their relative formulations with dimethyl-amine heads. Furthermore, LNPs formulated with lipids presenting amide, reverse-amide, or urea linkers were positively charged at neutral pH. Nevertheless, all LNPs were homogeneous in size and had optimal mRNA encapsulation efficiency. The in vitro results yielded some evidence in favor of the use of positively charged lipids, most likely due to improved electrostatic interaction with cell membranes.

These trends partially translated the in vivo results, in which mLuc transfection was especially efficient in the lungs for the amide- and urea linker-containing lipids, especially for lipids 35 and 36. Conversely, the carbonate lipid 33 resulted in higher liver-specific mLuc expression, and the *N*-methyl-piperazine head and reverse ester structure of lipid 38 resulted in selective spleen transfection.

Notably, despite being very structurally similar to lipid 36, lipid 35 displayed a much higher transfection efficiency in the lungs of Cre-TdTomato mice, possibly due to the pivotal effect of the LNPs p*K*_a_ on their in vivo biodistribution.

Leveraging this lung tropism, we demonstrated how lipid 35 mmPE-LNPs induced a reduction of tumor burden and remarkably increased the survival of lung metastasis-bearing mice.

Despite the promising results discussed, further studies would be required to elucidate the mechanism behind the observed trends and the potential of lipid 35. In particular, the observed relevance of p*K*_a_ in radically increasing the LNP lung transfection efficiency across different cell populations suggests a common, underlying mechanism driving this phenomenon that is not both independent from LNP accumulation and specific cell lineage and therefore likely receptor-independent. Thus, we hypothesized that a higher p*K*_a_ could lead to improved transfection by improving intracellular trafficking and endosomal escape. On the other hand, the presence of the amide end urea linkers makes the ionizable lipids more resistant to hydrolysis but also slows down their biodegradation. Thus, further studies could address the long-term biocompatibility of lipid 35, with particular attention to repeated administrations that could lead to the accumulation of this lipid in the organism.

This study lays the groundwork for further investigations into the structure of ionizable lipids to achieve more specific organ transfection efficiency with particular attention to the molecular and cellular mechanisms involved in these processes.

## Experimental Section

### Chemicals and Cell Culture

1,2-Distearoyl-*sn*-glycero-3-phosphocholine (DSPC, cat. no. 850365P-1g), cholesterol (cat. no. 700000P-5g), and 1,2-dimyristoyl-rac-*glycero*-3-methoxypolyethylene glycol-2000 (PEG-DMG 2000, cat. no. 880151P-5g) were purchased from Avanti Polar lipids. Ethanol absolute (cat. no. 000525052100) and 2-propanol (cat. no. 001626052100) were provided by Bio-Lab. DPBS 1× was purchased from Gibco (Cat# 14190–169), PBS 10X from Hylabs (Cat# BP507/500D), DEPC-treated water from BioPrep (Cat# DPH20-500 ML), and 0.5 M citrate buffer solution from Thermo Scientific (pH = 4.5, Cat# J60024.AK).

Fetal bovine serum was purchased from Biowest (FBS, heat-inactivated, EU origin, Cat# S140H-500). l-Glutamine 200 mM (100×, cat. no. 25030-024), 0.25% trypsin–EDTA (1×, cat. no. 25200-114), penicillin–streptomycin solution (10,000 U/mL of penicillin +10,000 μg/mL of streptomycin, cat. no. 15140-122), DMEM (1×, cat. no. 41965-039), and RPMI Medium 1640 (1×, cat. no. 21875-034) were provided by Gibco.

RAW264.7 cells (cat. no. TIB-71), HeLa cells (cat. no. CCL-2), and HepG2 cells (cat. no. HB-8065) were provided by ATCC. Cell cultures were maintained in an incubator at 37 °C in a controlled atmosphere (5% CO_2_, 95% humidity) using the culture media required by the producer using T25 and T75 flasks.

### Ionizable Lipid Synthesis

See the Supporting Information.

### mRNAs

Chemically modified mRNAs encoding firefly luciferase (62 kDa) and Cre recombinase (39 kDa) were provided by BioNTech or purchased from TriLink Biotechnologies. Custom mRNA encoding mmPE-A was acquired from Trilink with the following open reading frame:

5′-ATGgccgaagaagctttcctcggcgacggcggcgacgtcagcttcagcacccgcggcacgcagaactggacggtgg agcggctgctccaggcgcaccgccaactggaggagcgcggctatgtgttcgtcggctaccacggcaccttcctcgaagc ggcgcaaagcatcgtcttcggcggggtgcgcgcgcgcagccaggacctcgacgcgatctggcgcggtttctatatcgcc ggcgatccggcgctggcctacggctacgcccaggaccaggaacccgacgcacgcggccggatccgcaacggtgccct gctgcgggtctatgtgccgcgctcgagcctgccgggcttctaccgcaccagcctgaccctggccgcgccggaggcggc gggcgaggtcgaacggctgatcggccatccgctgccgctgcgcctggacgccatcaccggccccgaggaggaaggcg ggcgcctggagaccattctcggctggccgctggccgagcgcaccgtggtgattccctcggcgatccccaccgacccgcg caacgtcggcggcgacctcgacccgtccagcatccccgacaaggaacaggcgatcagcgccctgccggactacgcca gccagcccggcaaaccgccgcgcgaggacctgaagTAA-3′

### Lipid Stability

The stability of lipids was assessed by HPLC (Agilent, 1260 Infinity II) equipped with Thermo Fisher’s CAD using a Poroshell 120 EC-C18 (2.7 μm, 3 × 150 mm) column following the reverse phase method (Solvent-A: 0.1% TFA in 55% MeOH, 15% IPA and 30% water; solvent-B: 0.1% TFA in 70% MeOH and 30% IPA). Briefly, 3 mg of lipid was dissolved in 1 mL of ethanol and a water–ethanol mixture (1:3) solvent and then filtered into Agilent scintillation vials. Three μL of the sample was injected into the HPLC-CAD instrument each time. The study was carried out over a period of 90 days at specific intervals (days 0, 1, 2, 3, 7, 14, 21, 28, 35, 60, and 90). The samples were stored at room temperature throughout the study. The stability study graph was plotted by taking time (days) on the *x*-axis and the percentage of lipid purity on the *y*-axis.

### Design of Experiment-Based Screening

The screening was designed by using JMP Pro. The CPP ranges selected were: 30 to 45% ionizable lipid molar proportion; 20% to 45% cholesterol molar proportion, 5% to 45% helper lipid molar proportion; PEG-DMG/PEG/OME ratio from 0 to 1; total lipid concentrations between 1 mM and 7 mM; formulation temperature between 20 and 60 °C; as possible previously published ionizable lipids; as helper lipids DSPC or DOPE, and an N/P ratio between 5 and 12 (Figure S1A). These ranges were used to create a custom design with 20 experimental runs, including center points and repeated measures. The selected CQAs were the particle size, PDI, and zeta potential measured by DLS, mRNA encapsulation measured by RiboGreen, as well as in vitro and in vivo Luc expression. After formulating and characterizing these formulations, JMP Pro was used to understand which CPPs were statistically significant in determining the CQAs. Then, the critical CPPs were selected to simulate in silico 10^4^ possible formulations and their relative projected features and transfection efficiency using Monte Carlo Simulation. Finally, ad hoc quality thresholds were applied (Figure S1C) to select a small range of possible optimal formulations.

### LNP Formulation

LNPs were formulated using a NanoAssemblr Benchtop device (catalog no. NIT0055) equipped with a heating block (catalog no. NIT0026) and relative cartridges (catalog no. NIS0009, Precision Nanosystems). The lipids were dissolved in absolute ethanol and kept at 55 °C. mRNA was diluted in a 25 mM citrate buffer (pH 4.5, Thermo Scientific, Cat# J60024.AK). To prepare the LNP organic phase, lipids were mixed in the following molar ratios: 40% ionizable lipid, 48.5% cholesterol, 1.5% PEG-DMG, and 10% DSPC to a final concentration of 6 mM. The N/P ratio of ionizable lipids to mRNA was 9. The lipid and aqueous phases were loaded in the NanoAssemblr in 1 and 3 mL syringes, respectively. The particles were assembled at 45 °C, using a flow rate ratio (FRR) of 3:1 (aqueous: ethanol), a total flow rate (TFR) of 12 mL/min, using a pre- and postwaste of 50 μL. Particles were subsequently dialyzed using MAXI GeBaFlex-tubes, 14 kDa MWCO (Gene Bio-Application LTD, cat. no. D050-100), against 1× PBS (Hylabs, cat. no. BP507/500D), which was replaced after 4 h. Particles were recovered after dialysis the next day.

### Size and Zeta-Potential Analysis of LNPs

The nanosize and ζ-potential of prepared mRNA-LNPs were analyzed by dynamic light scattering (DLS) using a Malvern Zetasizer (Malvern Instruments). Briefly, mRNA-LNPs were diluted in double-distilled water (1:50, volume ratio) and PBS (1:50, volume ratio) for ζ-potential and size measurements, respectively.

### RNA Encapsulation and Quantification

The Quant-iT RiboGreen RNA assay kit (Life Technologies) was used to measure the mRNA encapsulation in LNP. In summary, 2 μL of LNPs was diluted in a final volume of 350 μL of TE buffer (20 mM EDTA, 10 μM Tris–HCL) with or without Triton X-100 (0.5%, Sigma-Aldrich). The samples with Triton were incubated at 55 °C for 5 min. Following this, 100 μL of each sample was added in triplicate to a black 96-well plate (Costar, Corning), and 100 μL of TE buffer (0.5% v/v, RiboGreen reagent) was added to each well. The fluorescence was detected using a microplate reader (Biotek Industries) following the manufacturer’s protocol. To estimate the percentage of encapsulated RNA, eq 1 was used
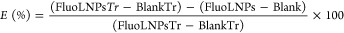
1where *E* is the encapsulation percentage of mRNA; FluoLNPs and FluoLNPTr are the fluorescence signals without and with Triton, respectively; Blank and BlankTr are the fluorescence signals of blanks without and with Triton, respectively.

### p*K*_a_ Measurement

As previously described, the p*K*_a_ values of LNPs were measured using the 2-(*p*-toluidino)-6-naphthalenesulfonic acid (TNS) assay.^33^ Briefly, the master buffer was prepared using 10 mM 4-(2-hydroxyethyl)-1-piperazineethanesulfonic acid (HEPES), 10 mM 4-morpholineethanesulfonic acid (MES), 10 mM ammonium acetate, and 130 mM sodium chloride (NaCl). Sixteen buffers with pH ranging from 2.5 to 10 were prepared using 1.0 M sodium hydroxide and 1.0 M hydrochloric acid based on the master buffer. The TNS reagent was prepared as a 0.1 mM stock solution in Milli-Q water. 90 μL of each buffer was added in triplicate to a black 96-well plate, and then 6 μL of 0.1 mM total lipid LNPs were added to each well. Then, 5 μL of the TNS stock solution was added to each well and kept on the shaker to mix properly for 10 min by covering the plate with aluminum foil. Fluorescence intensity was measured using excitation and emission wavelengths of 322 and 431 nm, respectively. p*K*_a_ curves were prepared by plotting the pH values on the *x*-axis and the normalized fluorescence values on the *y*-axis. Estimation of the p*K*_a_ values was performed using GraphPad Prism to perform nonlinear regression with variable slope.

### LNP In Vitro Testing

HeLa, HepG2, and RAW 264.7 cells were cultured according to the producer’s instructions, periodically checked to ensure the absence of Mycoplasma infections, and kept below passage 10. To test LNP activity in vitro, these different cell lines were seeded at a density of 10,000 cells per well in a 96-well plate. The day after seeding, cells were treated with LNPs at an mLuc mRNA concentration of 0.5 μg/mL, 0.25 μg/mL, and 0.125 μg/mL diluted in a complete cell culture medium. Every treatment was performed in quadruplicate. 24 h after treatment, cells were lysed using 50 μL of passive lysis buffer 1× (Promega) per well. Afterward, 30 μL of the cell lysates was pipetted in a white 96-well plate (Costar, White flat bottom, nontreated no lid, cat. no. 3912) and their luminescence was read using a GloMax plate reader equipped with dual injectors. 50 μL of the Luciferase Assay System (Promega) substrate was injected per well and the exposure time was set to 10 s.

### Animal Studies

All animal studies were performed in accordance with ethical guidelines and were approved by the Tel Aviv University Ethics Committee (Protocol # TAU-LS-IL—2201-108-3).

### LNP In Vivo Organ Biodistribution

Healthy, 8–10 weeks old, female C57BL/6J mice were injected retro-orbitally with a volume of LNPs loaded with luciferase-encoding mRNA (mLuc) corresponding to 10 μg of mRNA. 6 h after the injection, mice were injected intraperitoneally with 200 μL of 15 mg/mL of IVISbrite D-luciferin diluted in 1× PBS (PerkinElmer Cat#122799). After 5 min, mice were anesthetized with isoflurane and sacrificed by cervical dislocation. Their hearts, lungs, livers, spleens, and kidneys were harvested, and the Luc signal was measured using an IVIS Lumina device with automatic settings. For analysis, the pictures were processed using the Living Image Software (version 4.1) to measure the average radiance (measured in photons/sec/cm^2^/sr) within ad hoc defined ROIs. Every experimental group included 4 mice. Mice that displayed obvious signs of discomfort after LNP injections were sacrificed for ethical reasons and removed from the experiment.

### LNPs’ In Vivo Cellular Transfection

For this application, LNPs were loaded with mRNA encoding the Cre recombinase enzyme (GeneScript). Female, 8–10 weeks old B6g.Cg-Gt(ROSA)26Sortm9(CAG-tdTomato)/Hze/j mice (referred to as Cre-tdTomato mice) were injected retro-orbitally with a volume of particles corresponding to 20 μg of Cre mRNA. After 72 h, mice were anesthetized and sacrificed by cervical dislocation, and lungs, livers, and spleens were harvested.

Spleens were ruptured through 70 μm cutoff strainers, and the obtained single-cell suspension was centrifuged at 300*g* for 5 min at 4 °C. After pipetting the supernatant, cells were resuspended in red blood cell lysis buffer (Sigma-Aldrich) for 1 min quenched by adding 10 mL of 1× PBS and centrifuged at 300*g* for 5 min at 4 °C. After decanting the supernatant again, cells were resuspended in 0.5 mL of FACS buffer for cell counting and antibody staining.

Livers and lungs were processed using the Miltenyi mouse liver dissociation kit (Cat# 130-105-807) or the Miltenyi mouse lungs dissociation kit (Cat# 130-095-927), respectively, following the manufacturer’s instructions.

Cells were then incubated with mouse FcR blocker reagent (Cat# 130-092-575 Miltenyi) according to manufacturer’s instructions and then stained with different antibody panels as reported in Tables 1 and 2.

**Table 1:** Antibody Panel Used to Stain Cells Population in Mice Livers and Lungs to Assess tdTomato-Positive Cells

antigen	fluorophore	producer	catalog #
CD45	APC-fire 750	Biolegend	147714
CD31	AlexaFluor488	Biolegend	102414
CD326	APC	Biolegend	118213
CD11b	PE-Cy7	Biolegend	101215
CD11c	PerCP	Biolegend	117325

**Table 2:** Antibody Panel Used to Stain Cell Population in Mice Spleens to Assess tdTomato-Positive Cells

antigen	fluorophore	producer	catalog #
CD3e	PerCP	Biolegend	100325
CD19	FITC	Biolegend	152404
CD11b	APC-Cy7	Biolegend	101226
CD11c	APC	Biolegend	117309
F4/80	PE-Cy7	Biolegend	123113

After 30 min of incubation at 4 °C, cells were centrifuged at 500*g* for 5 min and finally resuspended in 100 μL of FACS buffer supplemented with DAPI 5 μg/mL before analyzing them on a Cytoflex Flow cytometer (Beckman) using the gating strategy summarized in Figures S13 and 14.

### LNPs’ In Vivo Cellular Transfection

To assess the LNPs’ biodistribution, LNPs were loaded with noncoding siRNA labeled with Cy5 and nonmodified Luc mRNA in a 50:50 (w/w) ratio. The particles were then injected IV in 10 week old female C57BL6/j mice at a dose of 10 μg of total mRNA per mouse. 18 h after administration, mice were sacrificed and their lungs, livers, and spleens were harvested and processed for flow cytometry as previously described. The extracted cells were then stained using the antibody panels summarized in Tables 3 and 4 and analyzed using the gating strategies analogous to the ones presented in Figures S13 and 14. LNP accumulation was measured as a percentage of Cy5-positive cells.

**Table 3:** Antibody Panel Used to Stain Cells Population in Mice Livers and Lungs to Assess Cy5-LNP Deposition

antigen	fluorophore	producer	catalog #
CD45	PE	Biolegend	103106
CD31	AlexaFluor488	Biolegend	102414
CD326	APC-AlexaFluor 700	Biolegend	118240
CD11b	PE-Cy7	Biolegend	101215
CD11c	PerCP	Biolegend	117325

**Table 4:** Antibody Panel Used to Stain Cells Population in Mice Spleens to Assess Cy5-LNP Deposition

antigen	fluorophore	producer	catalog #
CD3e	PerCP	Biolegend	100325
CD19	FITC	Biolegend	152404
CD11b	APC-Cy7	Biolegend	101226
CD11c	PE	Biolegend	117308
F4/80	PE-Cy7	Biolegend	123113

### Biocompatibility Studies

Female 8–10 weeks old, C57BL/6J mice were injected retro-orbitally with a volume corresponding to 10 μg of mRNA of LNPs loaded with luciferase-encoding mRNA (mLuc). 2 and 24 h after injection, approximately 300 μL of blood per mouse was recovered via cheek bleed in a Microtainer SST Blood collection tube (ref# 365,968, BD). To isolate the plasma from whole blood, the collection tubes were centrifuged at 3500 rpm for 10 min. The supernatant plasma was recovered in 1.5 mL Eppendorf tubes and stored at −80 °C for future analysis.

The concentrations of TNF-α, IL-6, MCP-1, and IL-6 in the plasma were measured using ad hoc ELISA kits from R&D Systems: Mouse CCL2/JE/MCP-1 DuoSet ELISA (ref # DY 479), Mouse IL-6 DuoSet ELISA (ref # DY 406), Mouse IL-10 DuoSet ELISA (ref # DT 417), Mouse TNF-alpha DuoSet ELISA (ref # DY410).

The plasma concentrations of alkaline phosphatase (ALP), serum glutamic oxaloacetic transaminase (SGOT), and serum glutamate pyruvate transaminase (SGPT) were analyzed by AML Lab Services.

24 h after injection, the livers, spleens, and lungs were also harvested and fixed in a paraffin solution (Sigma-Aldrich). The tissue processing, embedding in paraffin, sectioning, and hematoxylin/eosin staining were performed by Histospek.

### Assessment of LNPs’ Efficacy in a Lung Metastatic Model

The protocols were adapted from ref (54). B16F10.9 cells (5 × 10^5^ diluted in 100 μL of PBS) were administered intravenously into 10- to 12 week old male C57BL/6 mice. Treatment groups (*n* = 7 mice/group) included (1) mock treatment (1× PBS), (2) mLuc-loaded lipid 35 LNP, (3) mmPE-loaded SM-102 LNPs, and (4) mmPE-loaded lipid 35 LNP. An additional group of untreated tumor-free mice served as control (*n* = 7). The dose in each formulation was 0.09 mg/kg mRNA encoding PE.^52^ Treatments were administered on days 1, 5, and 9 from tumor inoculation. Administration was by intravenous injection of 100 μL of the selected formulation to the lateral tail vein using 26-gauge needles.

Two independent experiments were run: one to evaluate lung metastatic burden and the other to evaluate survival.

For mmPE-LNP, the general toxicity was evaluated using liver enzyme levels (AST and ALT), as well as ALP, urea, bilirubin, total protein, and creatinine measured in the blood. Mice bearing B16F10.9 tumors (B16F10.9) received three doses of either PBS, mLuc-loaded lipid 35 LNP, mmPE-loaded SM-102 LNPs, or mmPE-loaded lipid 35 LNPs at a dose of 0.09 mg/kg mRNA (*n* = 4 mice/group). AST and ALT levels were assessed 24 and 72 h from the end of treatment while the other markers were measured 72 h post-treatment.

For evaluation of lung metastatic burden, the experiment was terminated 20 days post-tumor injection. The lungs of all animals in the experiment were removed, weighed, and fixed in Blouin’s solution. The increase in lung weight was calculated using eq 2(53)
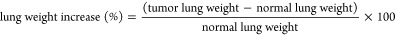
2

Surface metastases were counted, using a dissecting microscope, by a pathologist blinded to the experimental groups involved. For evaluation of survival, 7 animals were included in each experimental group. After the end of treatment, they were monitored every other day and the experiment was terminated on day 38. Statistical significance for the difference in survival was assessed using the Log-rank (Mantel–Cox) Test.

### LNP Stability under Storage

After assembly, LNPs formulated using the lead lipids (lipids 14, 35, and 36) were stored as suspension in 1× PBS in sealed 2 mL clear glass vials (Merck, catalog no. 27265) and kept at 4 °C. A small nanoparticle aliquot was withdrawn, and DLS, Ribogreen, and in vitro Luciferase assay were performed as previously presented at day 1 and then every 7 days until 2 months.

### Statistical Analysis

Data are presented as the data mean ± the standard error. Before analysis, normality tests were performed to assess the data Gaussian distributions. Statistical comparisons of two different groups were performed using a two-tailed paired *t*-test. For experiments involving multiple groups, one-way ANOVA with multiple comparison post-hoc tests was employed instead. All analyses were performed on Graph Pad Prism version 5.00 (GraphPad Software, San Diego, California, USA, www.graphpad.com). To elucidate possible correlations between the LNP sizes, zeta potentials, p*K*_a_, and in vitro and in vivo signals, we performed correlation analysis using GraphPad Prism. The correlations were assessed by plotting variables in pairs and assessing the function in GraphPad Prism. *P* values below 0.05 were considered statistically significant. Significance intervals for p were designed as follows: * for *p* < 0.05, ** for *p* < 0.01, *** for *p* < 0.001.

## Supporting Information Available

DoE-based LNP optimization; p*K*_a_ measurements; agarose gel electrophoresis of LNP formulations; correlation analysis between LNP features and their in vitro or in vivo transfection; radiance measure in the mice hearts and kidneys; proportional signal of LNPs formulated with different lipids among mice lungs, livers, and spleens; schematic representation of tdTomato expression; characterization of mCre-loaded LNPs; cellular biodistribution of LNPs in tdTomato mice; cellular biodistribution of Cy-5-labeled LNPs in vivo; safety profile of mmPE-A-loaded LNPs; gating strategies used for flow cytometry; and synthesis and characterization of the ionizable lipids (PDF)

## Abbreviations

ALP: alkaline phosphatase
ALT: alanine aminotransferase
AST: aspartate aminotransferase
CAD: charged aerosol detector
CPP: critical process parameter
CQA: critical quality attribute
HPLC: high-performance liquid chromatography
IL-6: interleukin-6
IL-10: interleukin 10
LNP: lipid nanoparticles
MCP-1: monocyte chemoattractant protein-1
mCre: Cre-encoding mRNA
mLuc: firefly luciferase-encoding mRNA
mPE-A: pseudomonas exotoxin A-encoding mRNA
PCC: Pearson’s correlation coefficient
PEG: polyethylene glycol
TNF-α: tumor necrosis factor-α
ζ: zeta potential.
