# Supplementary material for: Ionizable Lipids with Optimized Linkers Enable Lung-Specific, Lipid Nanoparticle-Mediated mRNA Delivery for Treatment of Metastatic Lung Tumors
Source: ACS Nano. 2025 Feb 6;19(6):6571–87. doi: 10.1021/acsnano.4c18636 (PMC11841047; doi:10.1021/acsnano.4c18636)
Supplement: Supplementary file 1 — nn4c18636_si_001.pdf [file nn4c18636_si_001.pdf]

Supporting Information For:

Ionizable Lipids with Optimized Linkers Enable Lung-Specific, Lipid Nanoparticles-Mediated mRNA Delivery for Treatment of Metastatic Lung Tumors

Gonna Somu Naidu[1-4‡], Riccardo Rampado[1-5‡], Preeti Sharma[1-4], Assaf Ezra[1-4], Govinda Reddy Kundoor[1-4], Dor Breier[1-4], Dan Peer [1-4*]

1. Laboratory of Precision Nanomedicine, Shmunis School of Biomedicine and Cancer Research, Tel Aviv University, Tel Aviv-Yafo 69978, Israel.

2. Department of Materials Sciences and Engineering, Tel Aviv University Tel Aviv-Yafo 69978, Israel.

3. Center for Nanoscience and Nanotechnology, Tel Aviv University Tel Aviv-Yafo 69978, Israel.

4. Cancer Biology Research Center, Tel Aviv University Tel Aviv-Yafo 69978, Israel.

5. Department of Pharmaceutical Sciences, University of Padova, Padova 35131, Italy.

‡These authors contributed equally.

*** Corresponding author, Dan Peer, peer@tauex.tau.ac.il**

Key words: Ionizable lipids; Lipid nanoparticle; mRNA delivery; Biodegradable linkers; lung delivery; Genetic medicines

TABLE OF CONTENTS

S. No.	Contents	Page No.
1	Supplementary Figure 1	3
2	Supplementary Figure 2	4
3	Supplementary Figure 3	5
4	Supplementary Figure 4	6
5	Supplementary Figure 5	7
6	Supplementary Figure 6	8
7	Supplementary Figure 7	9
8	Supplementary Figure 8	10
9	Supplementary Figure 9	10
10	Supplementary Figure 10	11
11	Supplementary Figure 11	12
12	Supplementary Figure 12	13
13	Supplementary Figure 13	14
14	Supplementary Figure 14	15
15	Synthesis of Ionizable Lipids	16-41
16	Synthesis of Lipid 32	20, 21
17	Synthesis of Lipid 33	22, 23
18	Synthesis of Lipid 34	24, 25
19	Synthesis of Lipid 35	26, 27
20	Synthesis of Lipid 36	28, 29
21	Synthesis of Lipid 37	30, 31
22	Synthesis of Lipid 38	32, 33
23	Synthesis of Lipid 39	34, 35
24	Synthesis of Lipid 40	36, 37
25	Synthesis of Lipid 41	38, 39
26	Synthesis of Lipid 42	40, 41

Supplementary Figures:

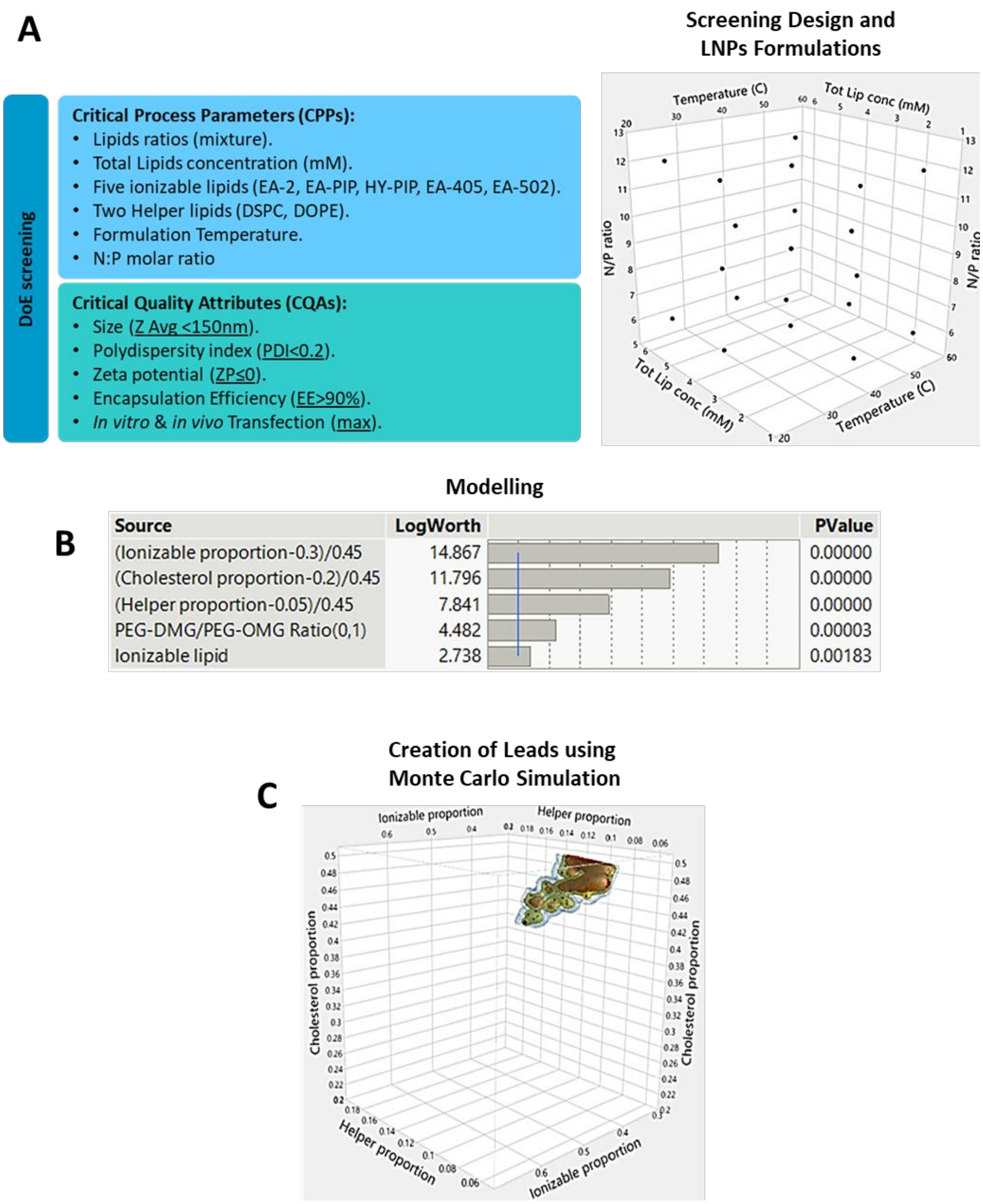

Supplementary Figure 1: Summary of the Design of Experiment-based screening for particle composition. The screening design consisted in twenty experimental runs, including replicate runs

and center points (A). The final modeling revealed that the only statistically significant CPPs for were the mixture factors of the different lipid components, the amount of biodegradable PEGylated lipid, and the ionizable lipid identity (B). After performing data interpolation, we performed Monte Carlo Simulation of 100,000 hypothetical formulations, revealing that particles with desirable CQAs constraints all fell in a very limited range of the experimental space (C). This allowed us to select a small range of theoretical candidate LNPs from which we calculated the average to get a single lead formulation.

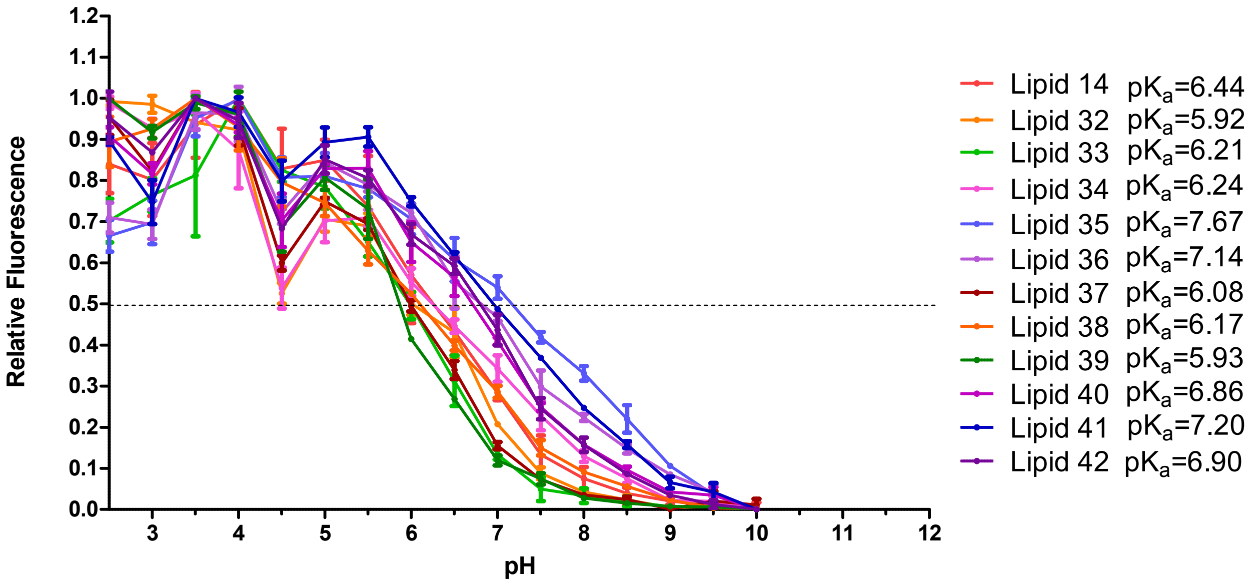

Supplementary Figure 2: Graph plotting pK_a of the LNP. pK_a values of the mRNA-encapsulated LNP were evaluated by TNS assay. pK_a values were calculated based on the 3-independent experiments.

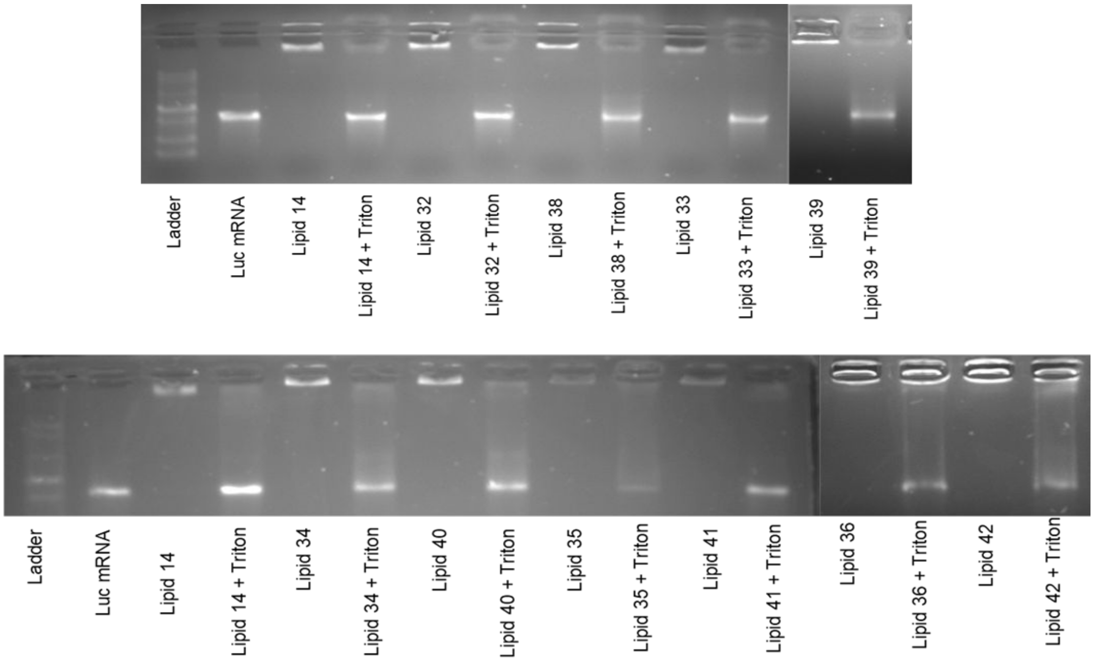

Supplementary Figure 3: Representative image of agarose gel electrophoresis of LNP formulated using different ionizable lipids.

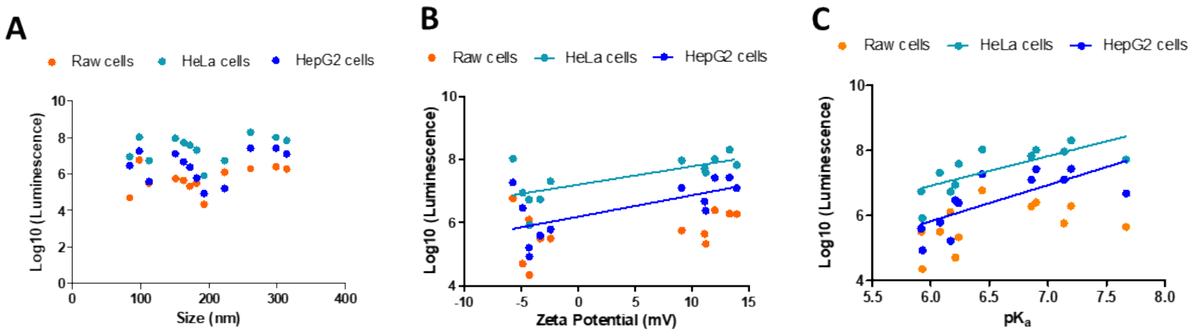

Parameter	HeLa cells	HepG2 cells
Pearson r	0.6893	0.6504
P value (two-tailed)	0.0132	0.022
P value summary	*	*
R square	0.4751	0.423

Parameter	HeLa cells	HepG2 cells
Pearson r	0.7358	0.7226
P value (two-tailed)	0.0064	0.0079
P value summary	**	**
R square	0.5414	0.5221

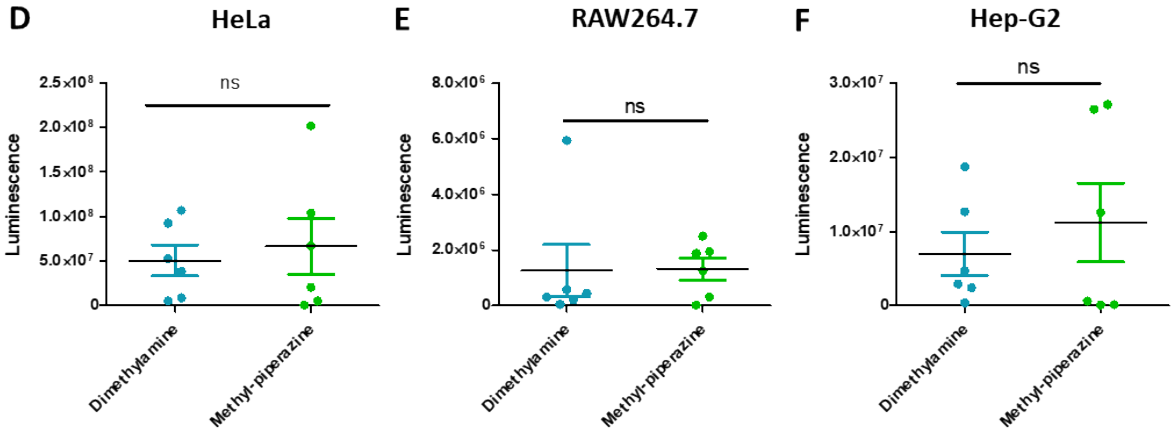

Supplementary Figure 4: Graphs plotting the possible correlations between LNP Size (A), pK_a (B), and Zeta potential (C) and the Luminescence measured across different cell lines. For each significant correlation, the best fitting line and statistics are reported in the relative table.

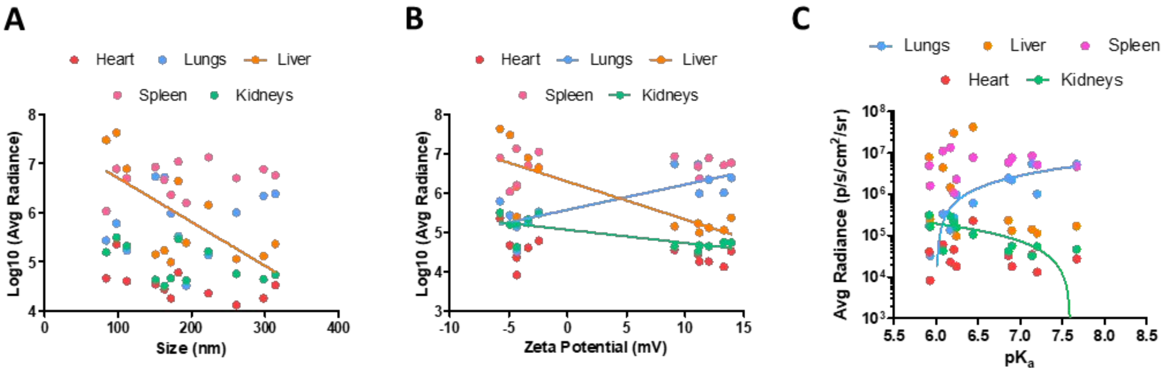

Parameter	Liver
Pearson r	-0.6741
P value (two-tailed)	0.0162
P value summary	*
R square	0.4544

Parameter	Lungs	Liver	Kidneys
Pearson r	0.7984	-0.8193	-0.7676
P value (two-tailed)	0.0019	0.0011	0.0036
P value summary	**	**	**
R square	0.6374	0.6712	0.5892

Parameter	Lungs	Kidneys
Pearson r	0.8417	-0.6978
P value (two-tailed)	0.0006	0.0116
P value summary	***	*
R square	0.7084	0.4869

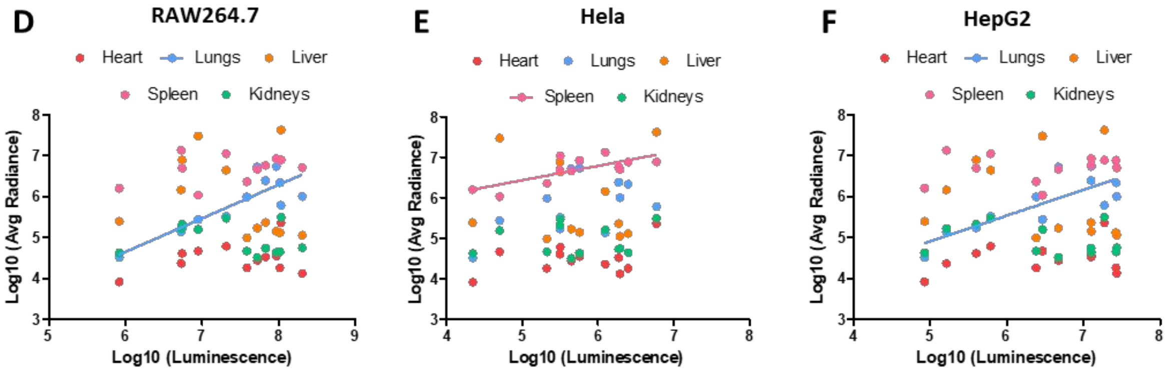

Parameter	Spleen
Pearson r	0.7746
P value (two-tailed)	0.0031
P value summary	**
R square	0.6

Parameter	Lungs
Pearson r	0.8843
P value (two-tailed)	0.0001
P value summary	***
R square	0.7821

Parameter	Lungs
Pearson r	0.8428
P value (two-tailed)	0.0006
P value summary	***
R square	0.7103

Supplementary Figure 5: Graphs plotting the possible correlations between LNP Size (A), Zeta potential (B), pK_a (C), RAW264.7 (D), HeLa (E) and HepG2 (F) cells luminescence, and the radiance measured in all the different analyzed organs in vivo. For each significant correlation, the best fitting line and statistics are reported in the relative table.

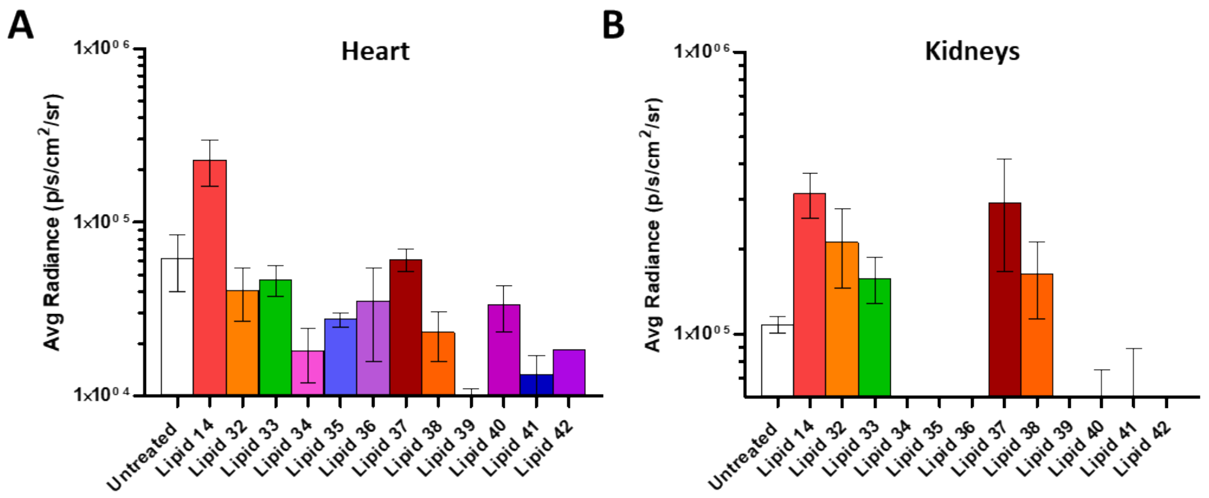

Supplementary Figure 6: Average Luc radiance measured murine hearts (A) and kidneys 6 hours after mLuc-LNP injection.

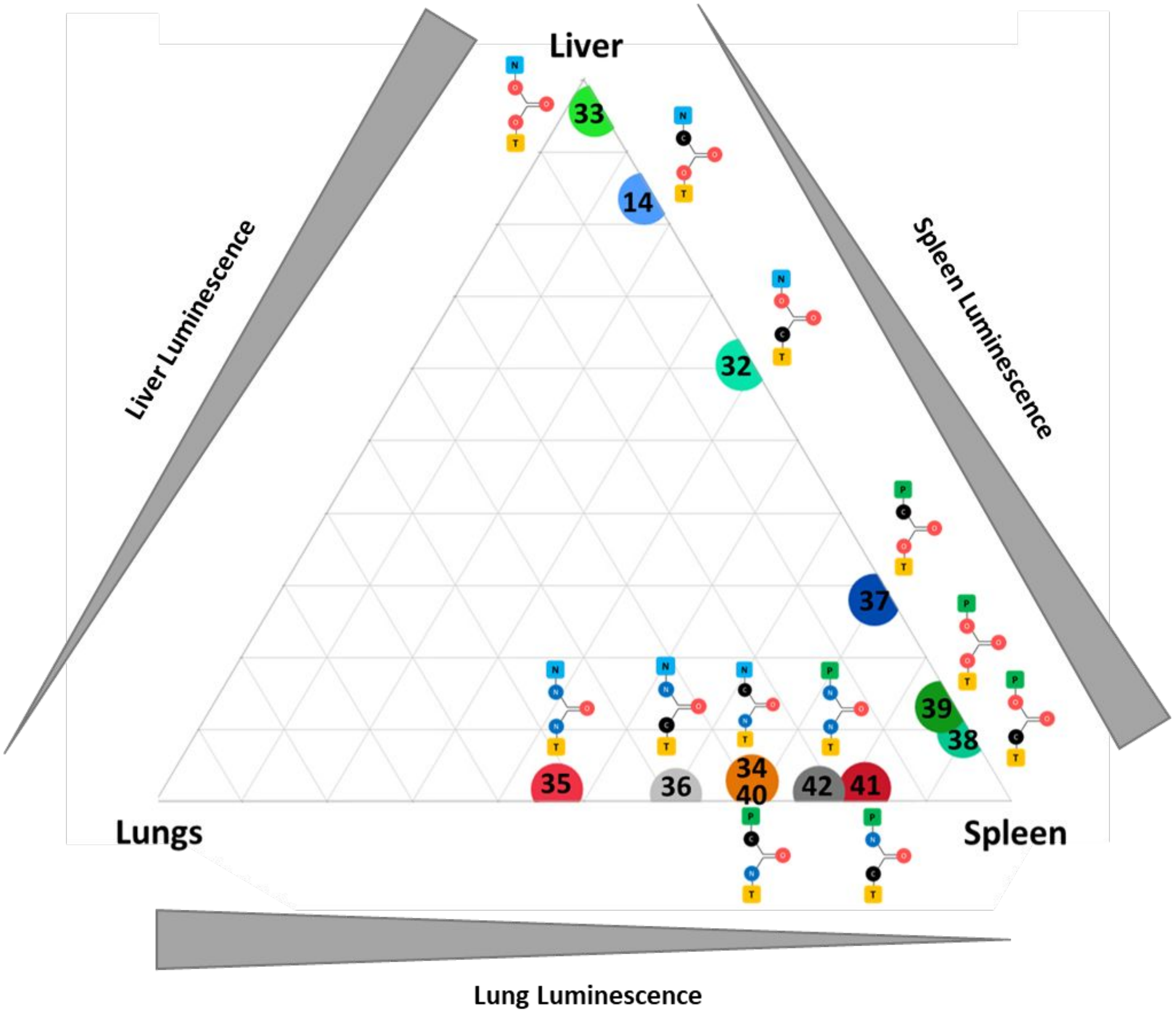

Supplementary Figure 7: The different tested ionizable lipids were plotted by their proportional average radiance measured in the lungs, livers, and spleen. For every lipid, the chemical structure of the ionizable lipids linkers is summarized (N= dimethylamine head group; P= N-methyl-piperazine head group; T= hydrophobic tails).

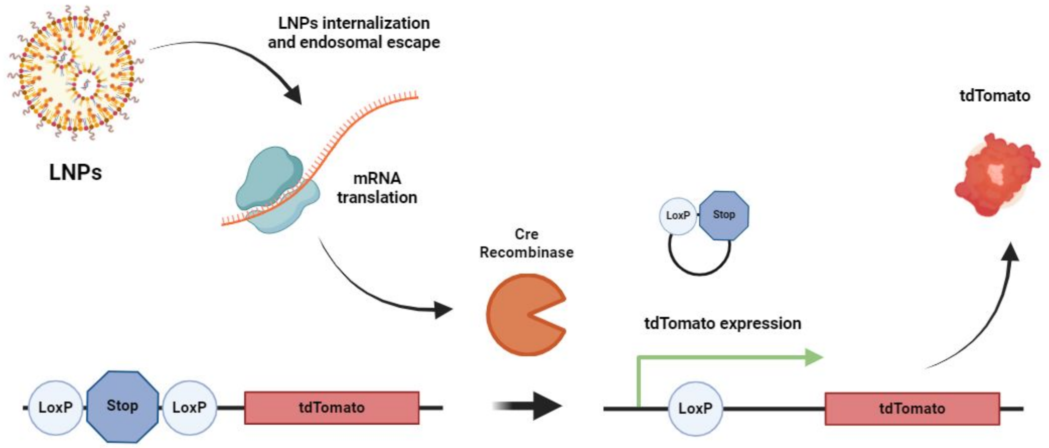

Supplementary Figure 8: Schematic representation of the mechanism behind Cre-induced tdTomato expression (Created with Biorender).

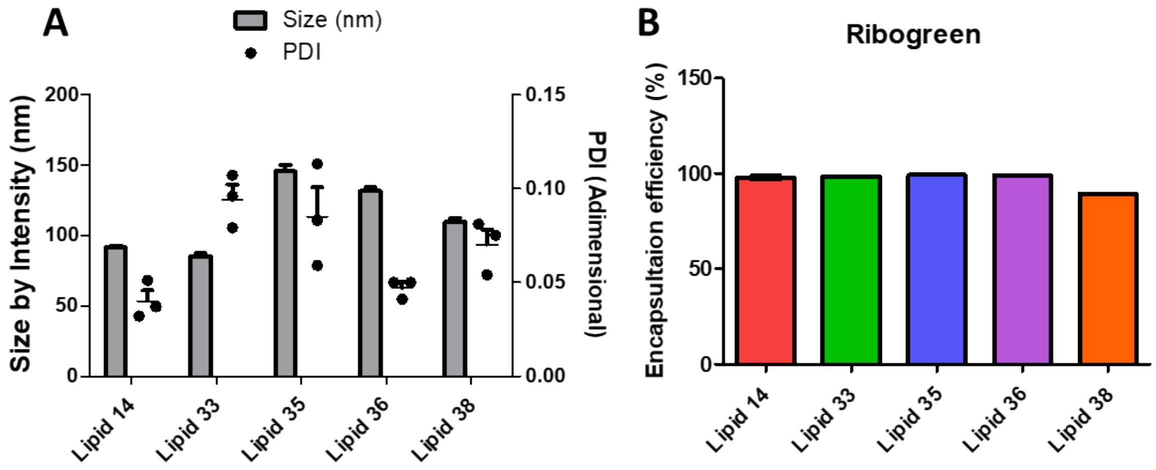

Supplementary Figure 9: Assessment of mCre-loaded LNP size and PDI (A) and encapsulation efficiency as measured by Ribogreen.

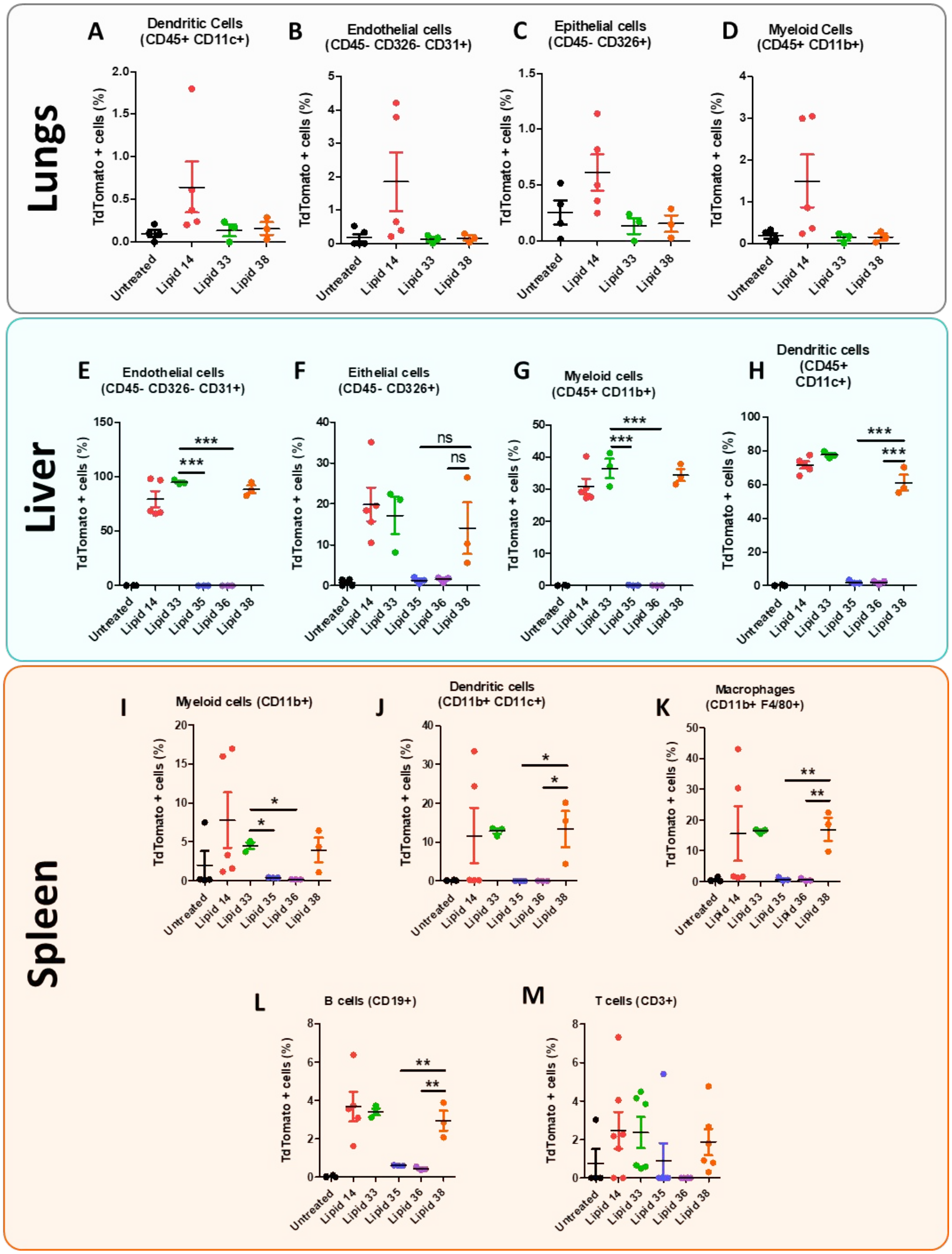
Lungs
A Dendritic Cells (CD45+ CD11c+)
B Endothelial cells (CD45- CD326- CD31+)
C Epithelial cells (CD45- CD326+)
D Myeloid Cells (CD45+ CD11b+)
Liver
E Endothelial cells (CD45- CD326- CD31+)
F Eithelial cells (CD45- CD326+)
G Myeloid cells (CD45+ CD11b+)
H Dendritic cells (CD45+ CD11c+)
Spleen
I Myeloid cells (CD11b+)
J Dendritic cells (CD11b+ CD11c+)
K Macrophages (CD11b+ F4/80+)
L B cells (CD19+)
M T cells (CD3+)
TdTomato + cells (%)
Untreated
Lipid 14
Lipid 33
Lipid 35
Lipid 36
Lipid 38
ns

Supplementary Figure 10: Flow cytometric analysis of the different cell lines dissociated from the Cre-tdTomato mice lungs (A-D), livers (E-H) and Spleens (I-M). (3-5 mice were included in each group. *: $p<0.05$; **:$p<0.01$; ***:$p<0.001$).

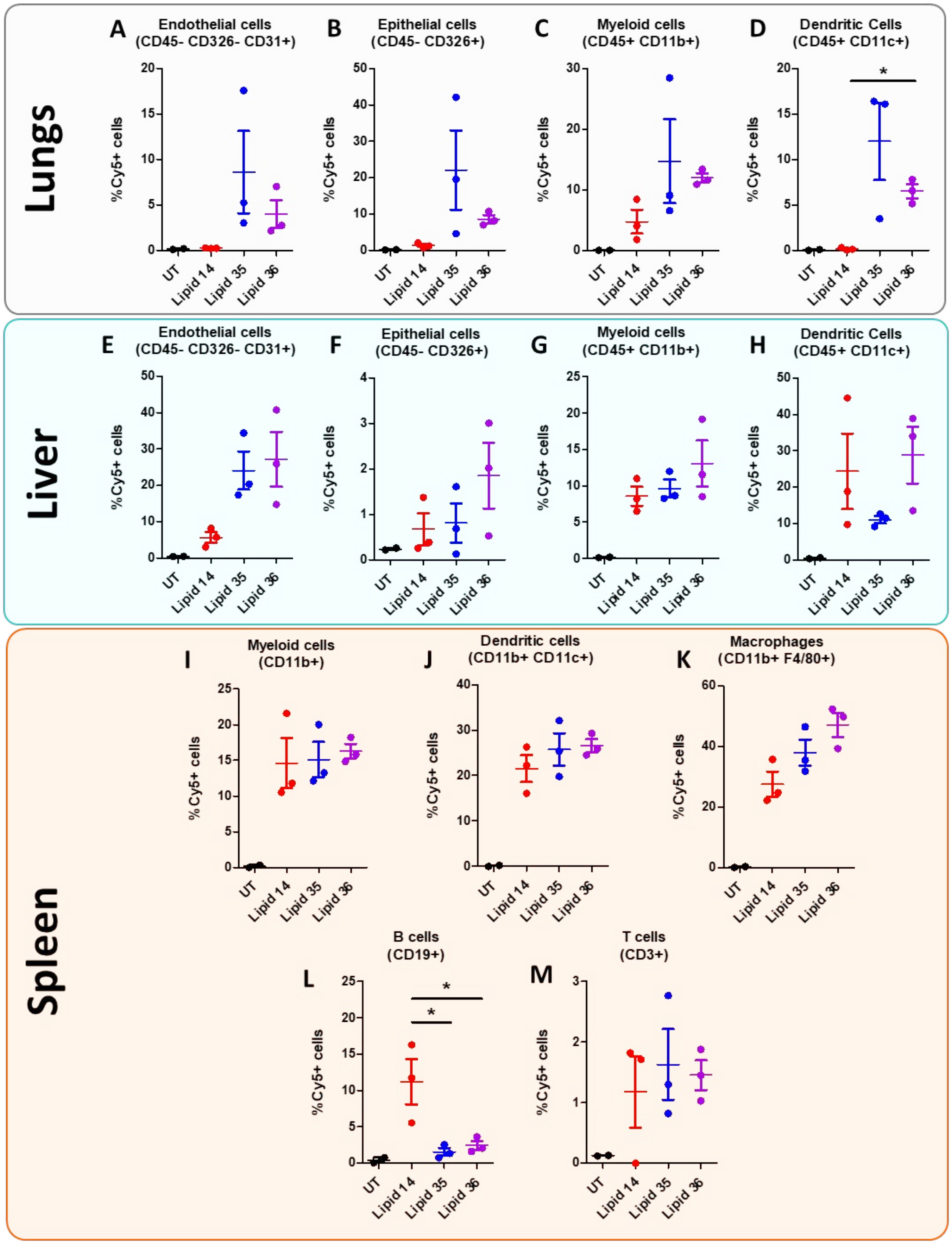

Supplementary Figure 11: Flow cytometric analysis of the different cell lines dissociated from the mice lungs (A-D), livers (E-H) and Spleens (I-M) after treatment with Cy5-labelled LNP. (3-5 mice were included in each group. *: p<0.05; **:p<0.01; ***:p<0.001).

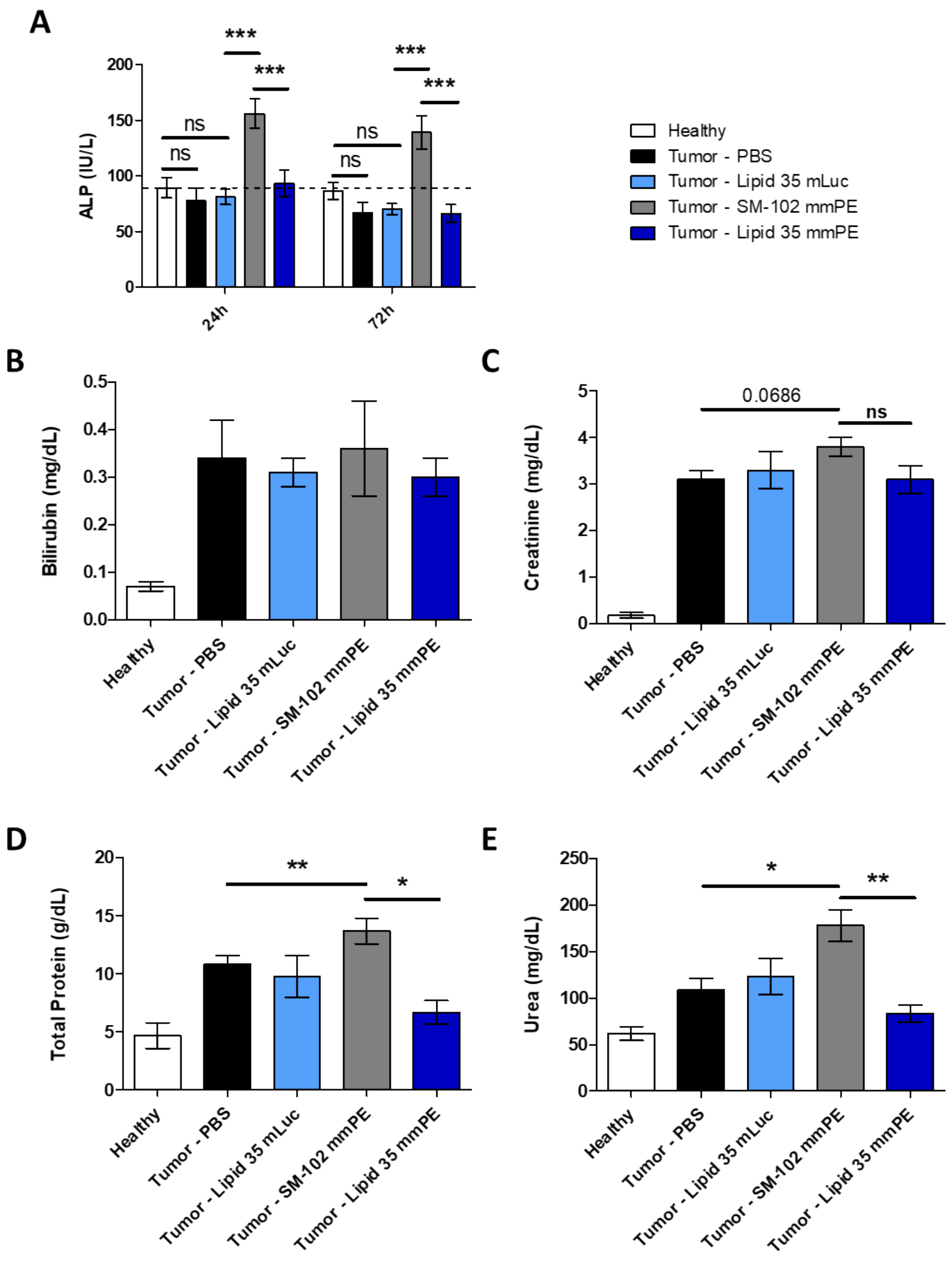

Supplementary Figure 12: Measurement of the plasma levels of ALP (A), Bilirubin (B), Creatinine (C), Total protein (D), Urea (E, 6 mice were included in each group).

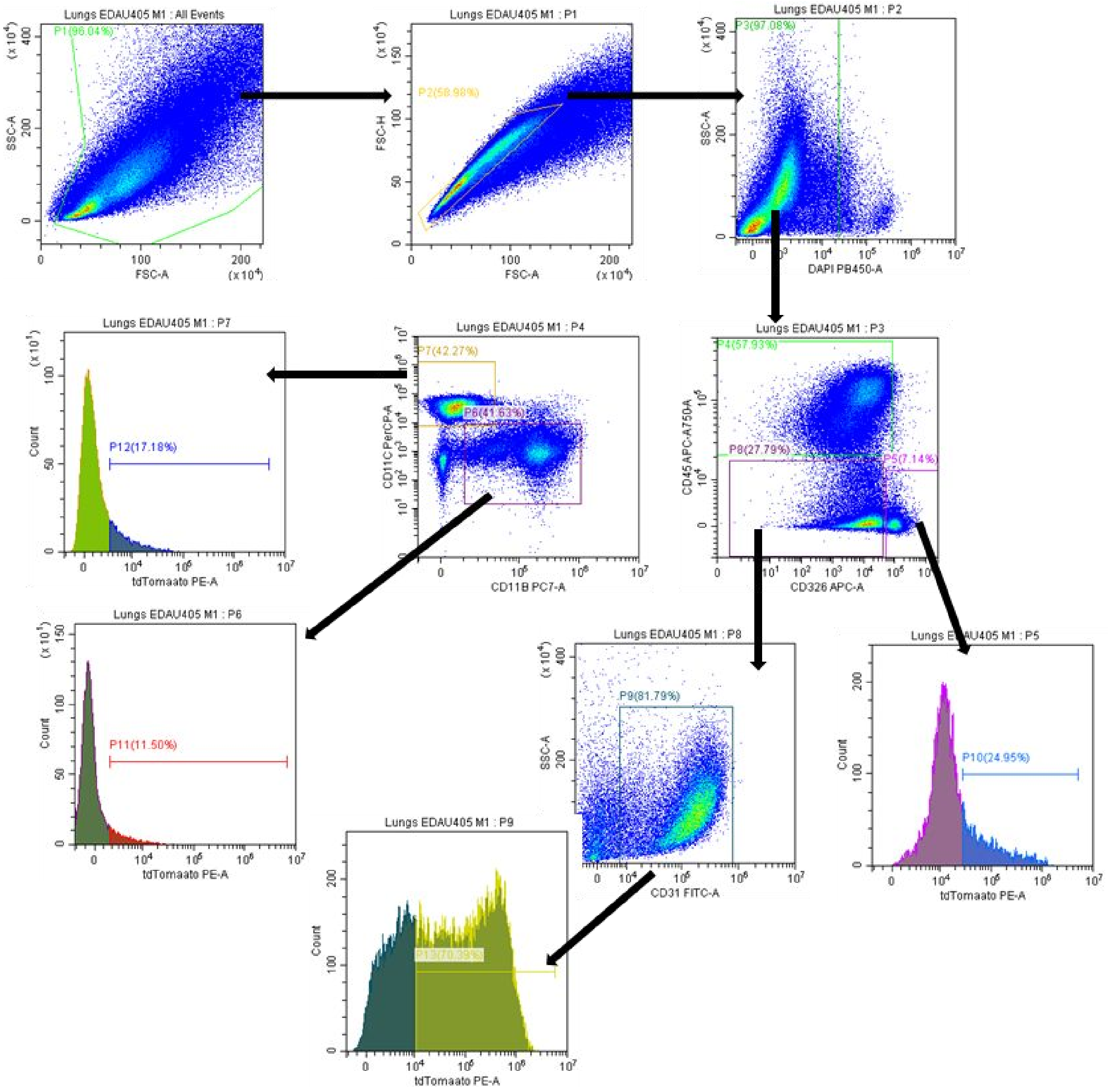

Supplementary Figure 13: Representative image of the gating strategy used to analyze the lungs and livers cell suspensions dissociated from Cre-tdTomato mice.

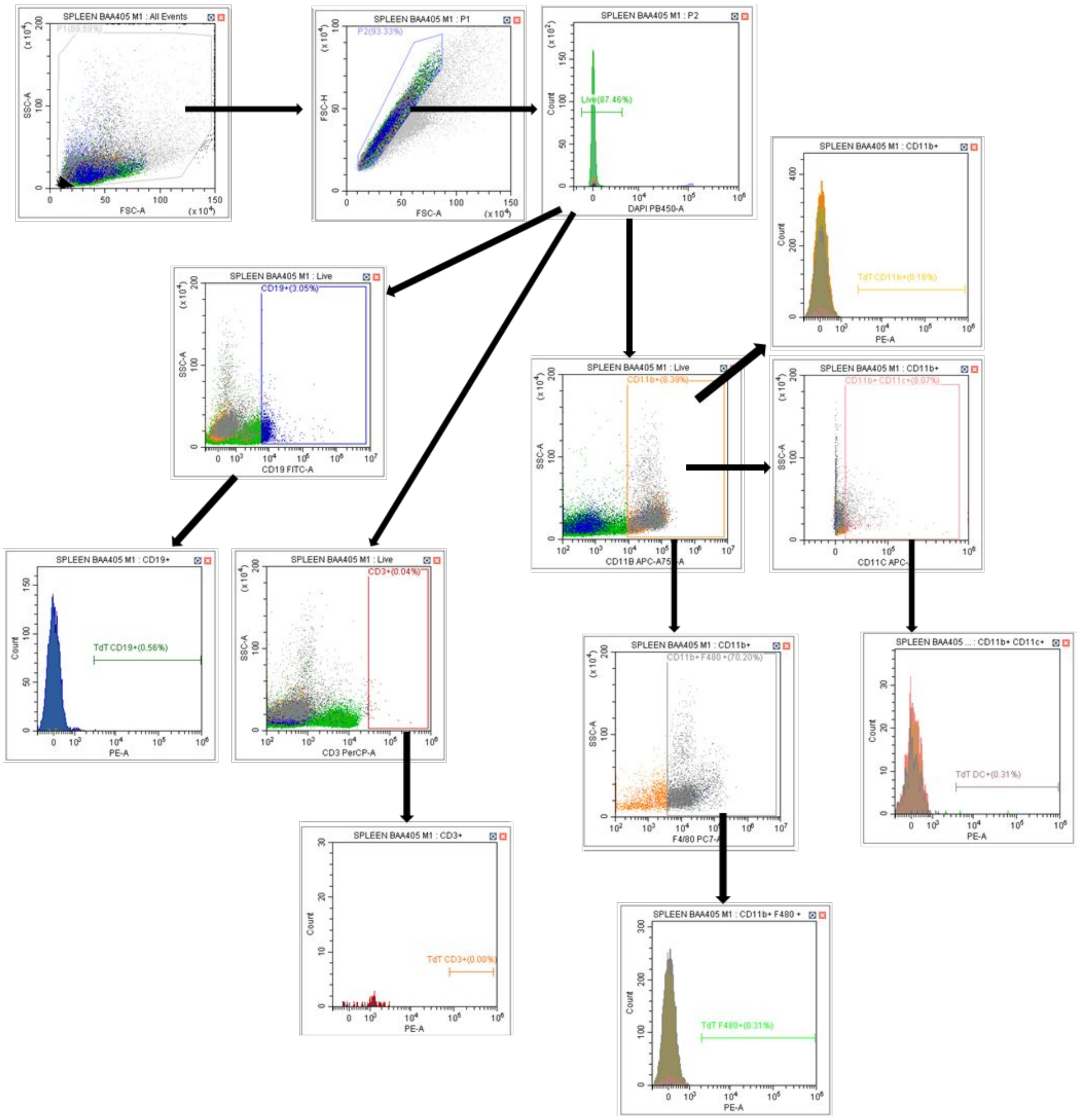

Supplementary Figure 14: Representative image of the gating strategy used to analyze the spleens cell suspensions dissociated from Cre-tdTomato mice.

Synthesis of Ionizable Lipids:

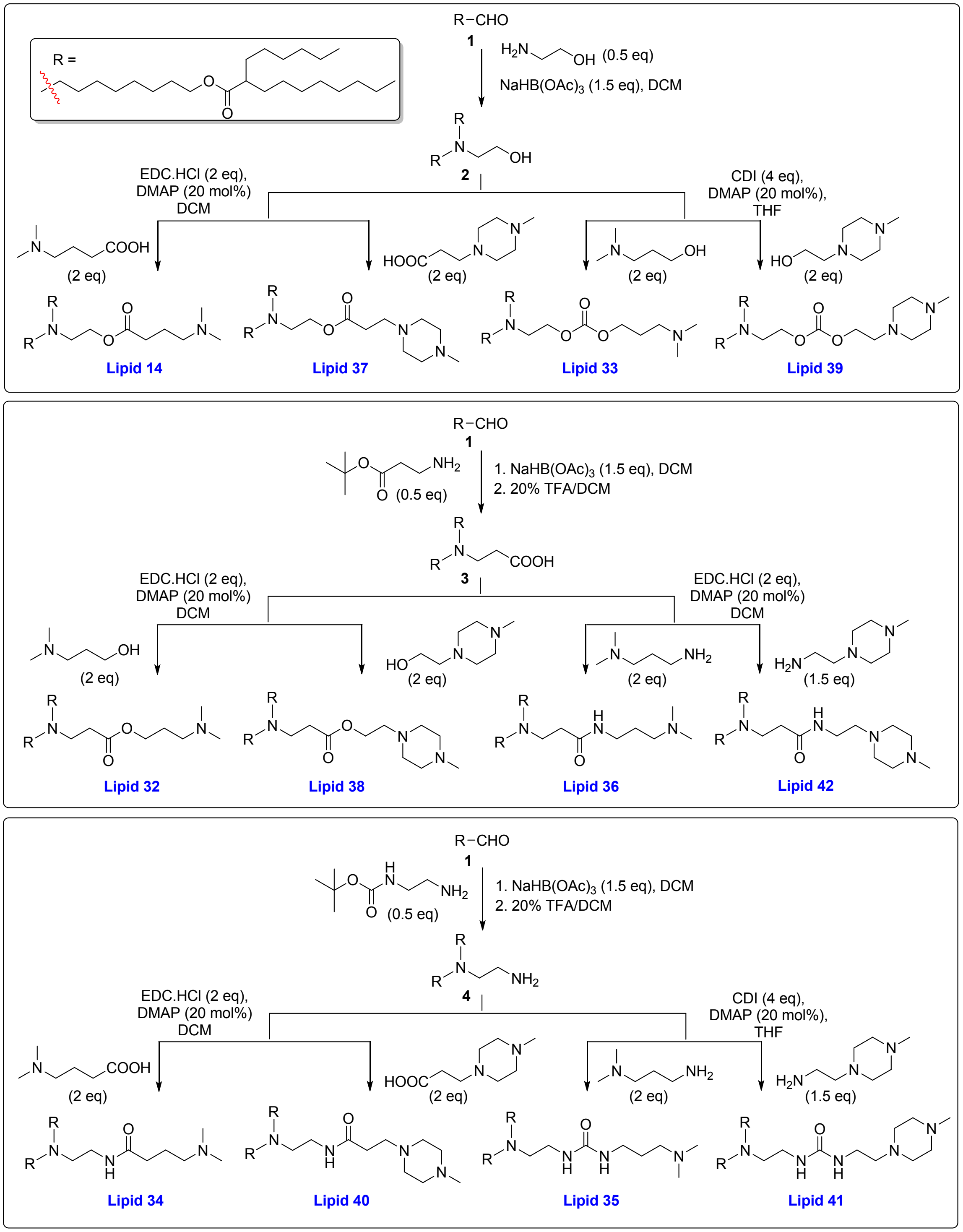

Supplementary Figure 15: Summary of synthetic routes for the synthesis of ionizable lipids.

General Information: All reactions were performed in oven-dried (120 °C) glass apparatus. All the chemicals were purchased from Sigma Aldrich unless mentioned. Anhydrous dichloromethane (CH_2Cl_2) and tetrahydrofuran (THF) were purchased from Aldrich. Thin Layer Chromatography (TLC) was carried out using Merck silica gel 60 F_{254} plates. Column chromatography was performed on high purity grade silica gel 60Å (230-400 mesh). ^{1}H NMR spectra were recorded on a 400 MHz spectrometer using $CDCl_3$ as the solvent and the spectra were referenced to residual chloroform (δ 7.26 ppm). Mass spectra were recorded on an ESI-TOF mass spectrometer.

The aldehyde **1** and **Lipid 14** were synthesized according to our previously reported procedures.[41]

((2-Hydroxyethyl)azanediyl)bis(octane-8,1-diyl) bis(2-hexyldecanoate)(2)

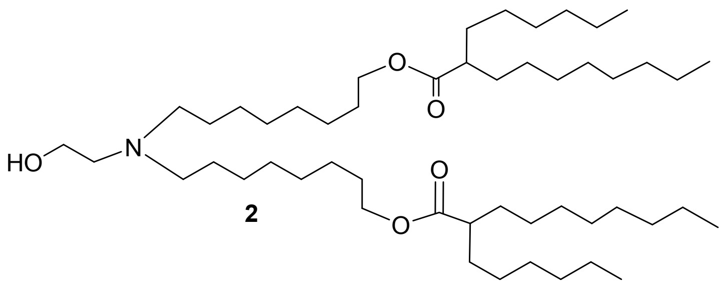

2

To a solution of 8-oxooctyl 2-hexyldecanoate **1** (760 mg, 2.0 mmol, 1 equiv.) in dry CH_2Cl_2 (20 mL), Ethanolamine (60 µL, 1.0 mmol, 0.5 equiv.) was added under argon atmosphere and stirred for 2 hr. at room temperature. After that, sodium triacetoxyborohydride (630 mg, 3.0 mmol, 1.5 equiv.) was added portion wise and left for the overnight stirring at the same temperature. Then, the reaction was quenched with sat. $NaHCO_3$ and followed by extract with CH_2Cl_2 (3 times). The organic portion was washed with brine solution and dried over anhydrous Na_2SO_4. The solvent was evaporated and the crude was purified by column chromatography using 0-6% isopropanol in $CHCl_3$ to get the desired alcohol **2** (620 mg, 80%) as colorless liquid.

^{1}H NMR (400 MHz, $CDCl_3$): δ 4.05 (4 H, t, J = 6.8 Hz), 3.54 (2 H, t, J = 5.6 Hz), 2.59 (2 H, t, J = 5.6 Hz), 2.46 (4 H, t, J = 7.6 Hz), 2.34-2.24 (2 H, m), 1.68-1.50 (8 H, m), 1.48-1.36 (8 H, m), 1.36-1.16 (56 H, m), 0.86 (12 H, t, J = 6.8 Hz).

ESI-MS: m/z 795.1 $[M+1]^+$

3-(Bis(8-((2-hexyldecanoyl)oxy)octyl)amino)propanoic acid (3)

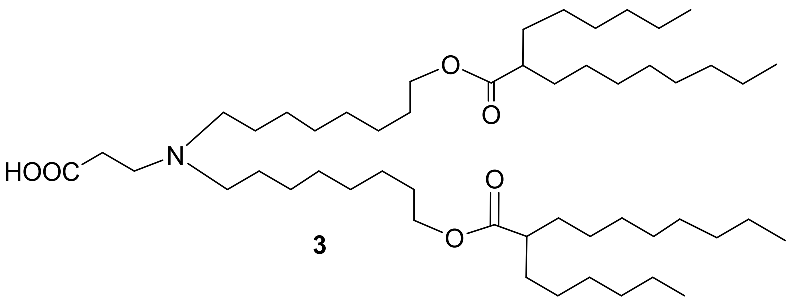

3

To a suspension of *β*-Alanine *tert*-butyl ester hydrochloride (428 mg, 2.36 mmol, 0.5 equiv.) in dry CH_2Cl_2 (5 mL), trimethylamine (0.33 mL, 2.36 mmol, 0.5 equiv.) was added under argon atmosphere and stirred for 5 min at room temperature. Then, a solution of 8-oxooctyl 2-hexyldecanoate (1.8 g, 4.71 mmol, 1.0 equiv.) in dry CH_2Cl_2 (15 mL) was added drop wisely and stirred for 2 hr. After that, the reaction mixture was diluted with another 10 mL of dry CH_2Cl_2, and sodium triacetoxyborohydride (1.5 g, 7.07 mmol, 1.5 equiv.) was added portion wise over a period of 10 min, and left for the overnight stirring at room temperature. Later, the reaction was quenched with sat.$NaHCO_3$ solution and extracted with CH_2Cl_2. The solvent was evaporated on rotary evaporator and the crude product was dissolved in 20% TFA/CH_2Cl_2 (20 mL) and stirred for 6 hr. at room temperature. Then, reaction was quenched with sat.$NaHCO_3$ solution and extracted with Ethyl acetate. After that, the organic portion was washed with water (2 times) and brine solution, respectively. The solvent was removed on the rotary evaporator, and the residue was purified by column chromatography using 0-15% isopropanol in chloroform to obtain acid **3** (1.62 g, 84%) as a pale yellowish liquid.

^{1}H NMR (400 MHz, $CDCl_3$): δ 4.05 (4 H, t, J = 6.8 Hz), 2.91 (2 H, t, J = 6.4 Hz), 2.70 (4 H, t, J = 8.0 Hz), 2.53 (2 H, t, J = 6.4 Hz), 2.36-2.24 (2 H, m), 1.68-1.50 (12 H, m), 1.48-1.36 (6 H, m), 1.36-1.16 (54 H, m), 0.87 (12 H, t, J = 6.8 Hz).

ESI-MS: m/z 823.2 $[M+1]^+$; 845.2 $[M+Na]^+$.

((2-Aminoethyl)azanediyl)bis(octane-8,1-diyl) bis(2-hexyldecanoate)(4)

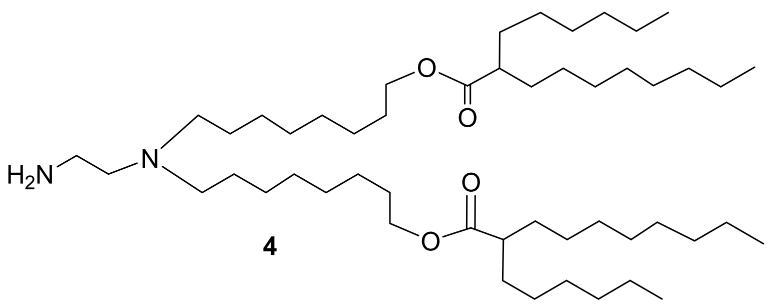

4

To a solution of 8-oxooctyl 2-hexyldecanoate (1.77 g, 4.63 mmol, 1.0 equiv.) in dry CH_2Cl_2 (20 mL), N-Boc-ethylenediamine (366 µL, 2.32 mmol, 0.5 equiv.) was added drop wisely and stirred for 2 hr. After that, the reaction mixture was diluted with another 20 mL of dry CH_2Cl_2, and sodium triacetoxyborohydride (1.47 g, 6.95 mmol, 1.5 equiv.) was added portion wise over a period of 15 min, and left for the overnight stirring at room temperature. Later, the reaction was quenched with sat.$NaHCO_3$ solution and extracted with CH_2Cl_2. The solvent was evaporated on rotary evaporator and the crude product was dissolved in 20% TFA/CH_2Cl_2 (40 mL) and stirred for 6 hr at room temperature. Then, reaction was quenched with sat.$NaHCO_3$ solution and extracted with Ethyl acetate. After that, the organic portion was washed with water (2 times) and brine solution respectively. The solvent was removed on the rotary evaporator, and the residue was purified by column chromatography using 0-15% isopropanol in chloroform to obtain amine **4** (1.60 g, 88%) as a pale yellowish liquid.

^{1}H NMR (400 MHz, $CDCl_3$): δ 4.06 (2 H, t, J = 6.8 Hz), 4.05 (2 H, t, J = 6.8 Hz), 2.93 (2 H, t, J = 6.0 Hz), 2.85-2.62 (2 H, br), 2.69 (2 H, t, J = 6.0 Hz), 2.57 (2 H, t, J = 7.6 Hz), 2.36-2.22 (2 H, m), 1.70-1.53 (10 H, m), 1.53-1.38 (6 H, m), 1.38-1.14 (56 H, m), 0.87 (12 H, t, J = 6.8 Hz).

ESI-MS: m/z 794.2 $[M+1]^+$.

((3-(3-(Dimethylamino)propoxy)-3-oxopropyl)azanediyl)bis(octane-8,1-diyl) bis(2-hexyldecanoate)(Lipid 32)

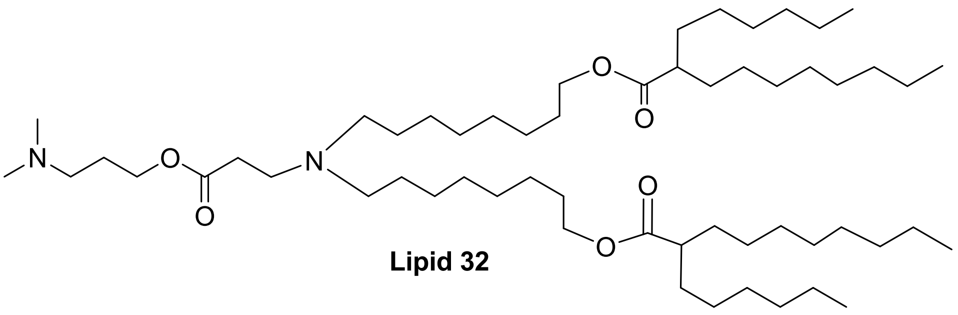

Lipid 32

The acid **3** (250 mg, 0.3 mmol, 1 equiv.), 3-Dimethylamino-1-propanol (72 µL, 0.61 mmol, 2 equiv.) and DMAP (7 mg, 0.06 mmol, 20 mol%) were dissolved in anhydrous Dichloromethane (15 mL) under argon atmosphere and stirred for 2 min. Then, EDC (116 mg, 0.61 mmol, 2 equiv.) was added it and stirred for 6 hr. at room temperature under argon atmosphere. After that, the reaction was quenched with sat. $NaHCO_3$ solution and extracted with ethyl acetate. Then the organic portion was washed water, brine solution, and dried over anhydrous Na_2SO_4 respectively. The solvent was evaporated on rotary evaporator, and the residue was purified by column chromatography using 0-10% isopropanol in $CHCl_3$ to obtain **Lipid 32** (175 mg, 63%) as pale yellowish liquid.

^{1}H NMR (400 MHz, $CDCl_3$): δ 4.12 (2 H, t, J = 6.4 Hz), 4.04 (4 H, t, J = 6.8 Hz), 2.89-2.74 (2 H, br), 2.57-2.35 (8 H, br), 2.35-2.18 (8 H, m), 1.92-1.75 (2 H, m), 1.68-1.51 (8 H, m), 1.51-1.38 (8 H, m), 1.36-1.16 (56 H, m), 0.87 (12 H, t, J = 6.8 Hz).

ESI-MS: m/z 908.2 $[M+1]^+$; 455.1 $[M/2+1]^+$

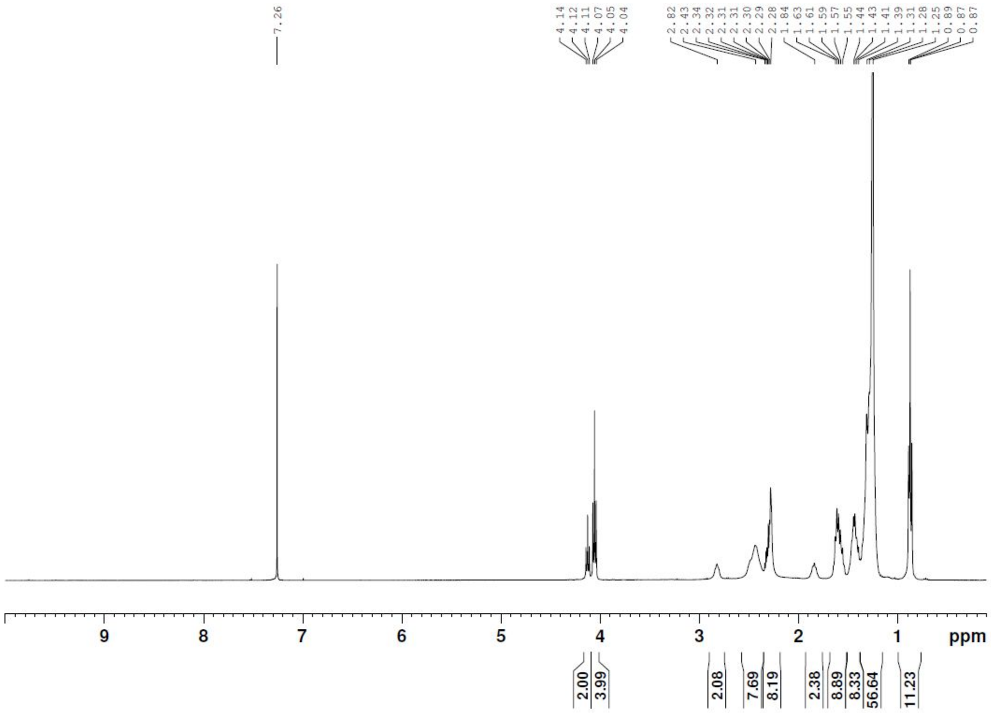

Supplementary Figure 16: ^{1}HNMR Spectrum of Lipid 32.

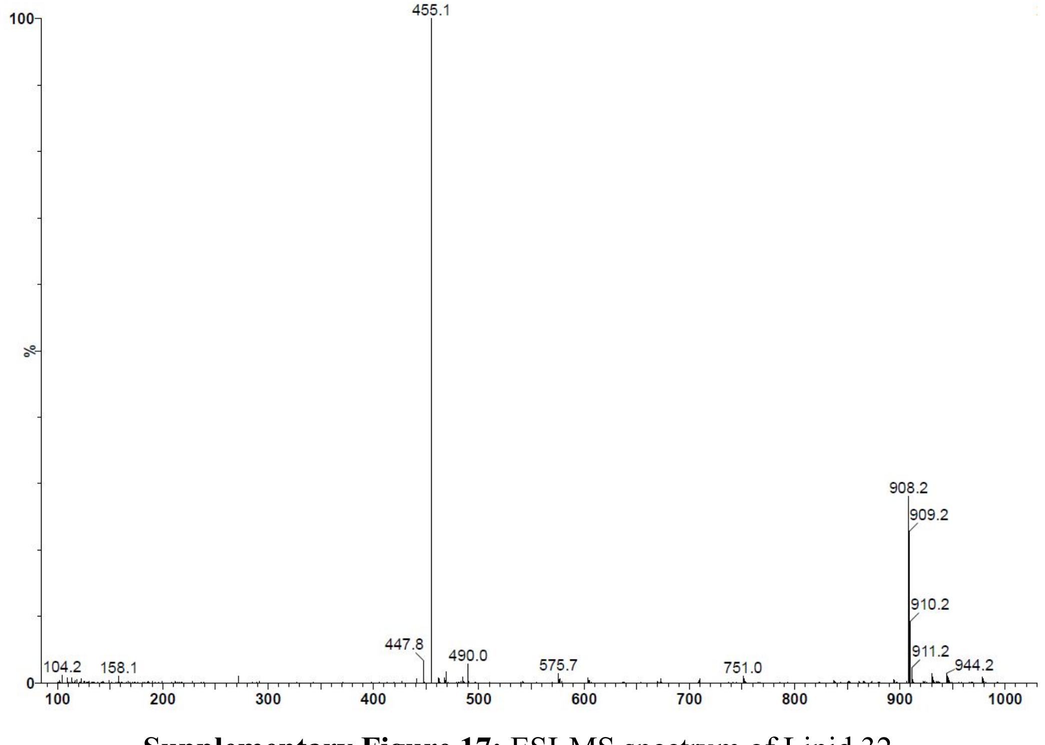

Supplementary Figure 17: ESI-MS spectrum of Lipid 32.

11-(8-((2-hexyldecanoyl)oxy)octyl)-2-methyl-7-oxo-6,8-dioxa-2,11-diazanonadecan-19-yl 2-hexyldecanoate (Lipid 33)

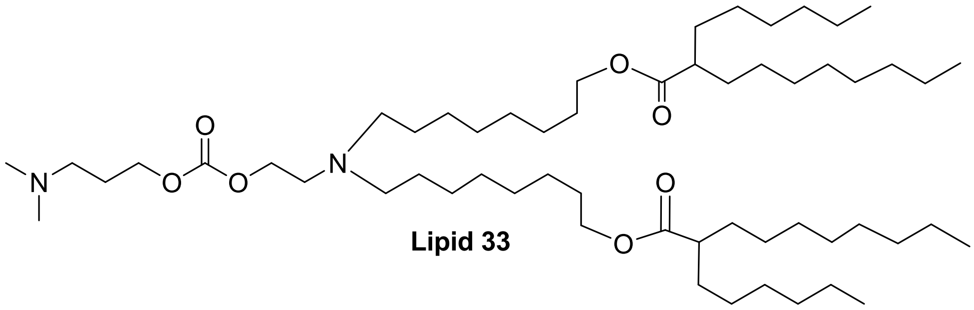

Lipid 33

To a solution of alcohol **2** (104 mg, 0.13 mmol, 1 equiv.) in anhydrous tetrahydrofuran (10 mL), CDI (85 mg, 0.52 mmol, 4 equiv.) and DMAP (3 mg, 0.03 mmol, 20 mol%) were added under argon atmosphere and stirred for 6 hr. at room temperature. Then, 3-Dimethylamino-1-propanol (31 mg, 0.26 mmol, 2 equiv.) was added and left for the overnight stirring at 60 °C under argon atmosphere. After that, THF was removed rotary evaporator and poured the reaction mixture in ethyl acetate (100 ml) and washed with sat. $NaHCO_3$, water and brine solution respectively. The solvent was dried over anhydrous Na_2SO_4 and evaporated rotary evaporator, and the residue was purified by column chromatography using 0-10% isopropanol in $CHCl_3$ to obtain **Lipid 33** (87 mg, 72%) as pale yellowish liquid.

^{1}H NMR (400 MHz, $CDCl_3$): δ 4.19 (2 H, t, J = 6.4 Hz), 4.17 (2 H, t, J = 5.4 Hz), 4.05 (4 H, t, J = 6.8 Hz), 2.73 (2 H, t, J = 6.0 Hz), 2.46 (6 H, t, J = 7.2 Hz), 2.38-2.19 (8 H, m), 1.97-1.83 (2 H, br), 1.70-1.50 (8 H, m), 1.49-1.37 (8 H, m), 1.37-1.12 (56 H, m), 0.87 (12 H, t, J = 6.8 Hz).

ESI-MS: m/z 924.2 $[M+1]^+$; 463.0 $[M/2+1]^+$

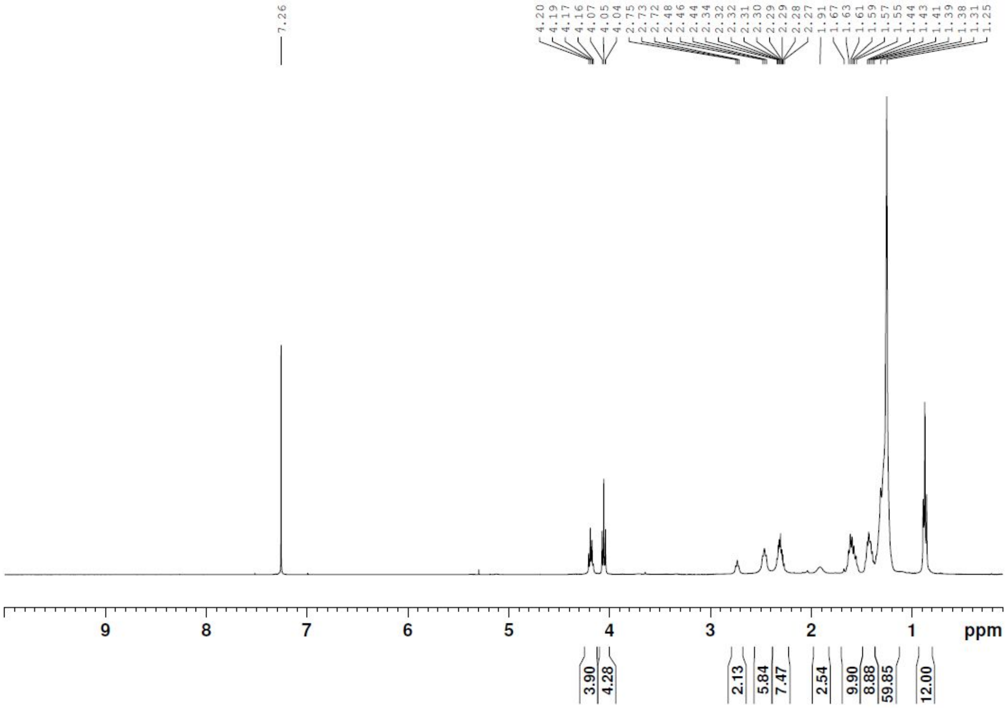

Supplementary Figure 18: ^{1}HNMR Spectrum of Lipid 33.

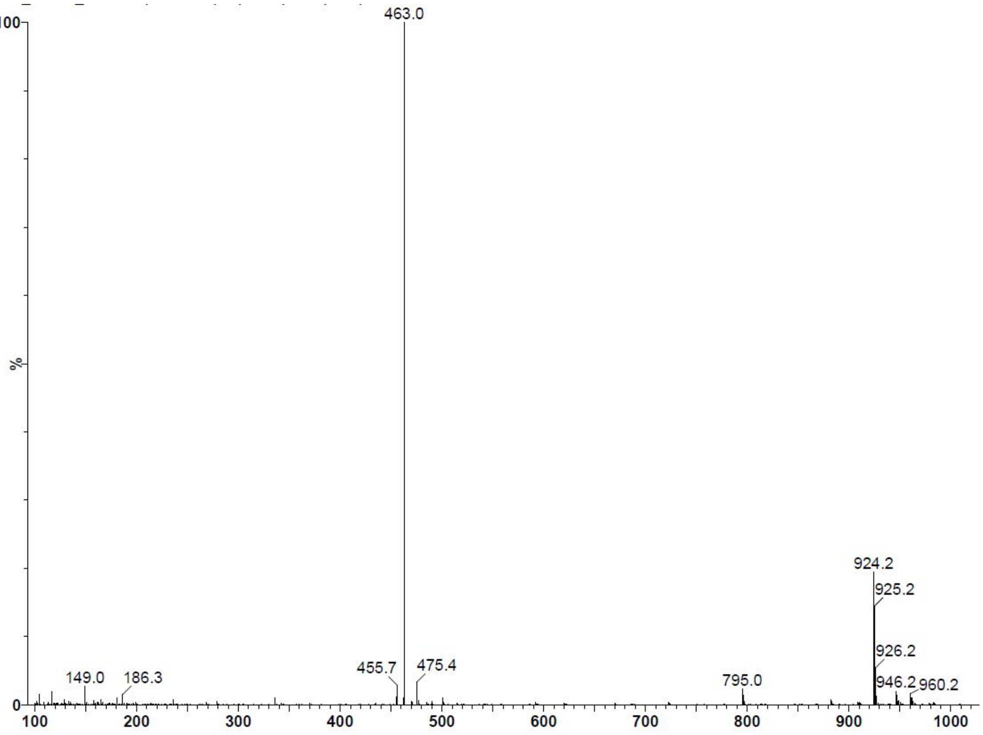

Supplementary Figure 19: ESI-MS spectrum of Lipid 33.

((2-(4-(dimethylamino)butanamido)ethyl)azanediyl)bis(octane-8,1-diyl) bis(2-hexyldecanoate) (Lipid 34)

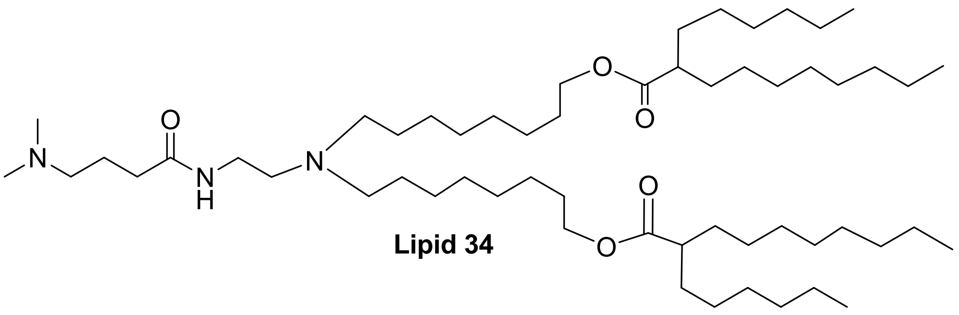

Lipid 34

The amine **4** (213 mg, 0.27 mmol, 1 equiv.), 4-(Dimethylamino)butyric acid hydrochloride (90 mg, 0.54 mmol, 2 equiv.), EDC.HCl (102 mg, 0.54 mmol, 2 equiv.) and DMAP (6 mg, 0.05 mmol, 20 mol%) were dissolved in dry CH_2Cl_2 (15 mL) under argon atmosphere and stirred for 6 hr. at room temperature. After that, the reaction was quenched with sat. $NaHCO_3$ followed by extract with Ethyl acetate (3 times). Then the organic portion was washed with water (2 times) and brine solution (1 time), and dried over anhydrous Na_2SO_4. The solvent was evaporated and the residue was purified by column chromatography using 0-15% Methanol in Dichloromethane to obtain **Lipid 34** (162 mg, 67%) as pale yellowish oil.

^{1}H NMR (400 MHz, $CDCl_3$): δ 4.06 (4 H, t, J = 6.8 Hz), 3.49-3.38 (2 H, m), 2.90-2.76 (2 H, br), 2.75-2.58 (6 H, br), 2.49 (6 H, s), 2.35 (2 H, t, J = 6.8 Hz), 2.33-2.25 (2 H, m), 1.98 (2 H, quint, J = 7.2 Hz), 1.69-1.49 (12 H, m), 1.49-1.37 (4 H, m), 1.37-1.13 (56 H, m), 0.87 (12 H, t, J = 6.8 Hz).

ESI-MS: m/z 907.2 $[M+1]^+$; 454.5 $[M/2+1]^+$

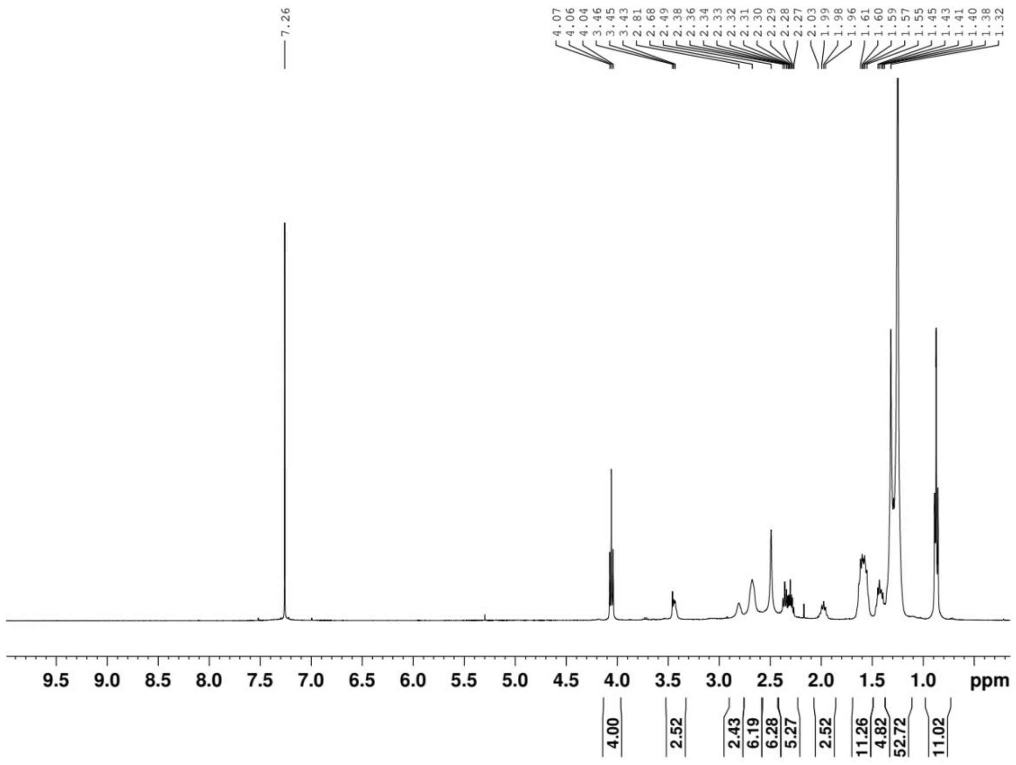

Supplementary Figure 20: ^{1}HNMR Spectrum of Lipid 34.

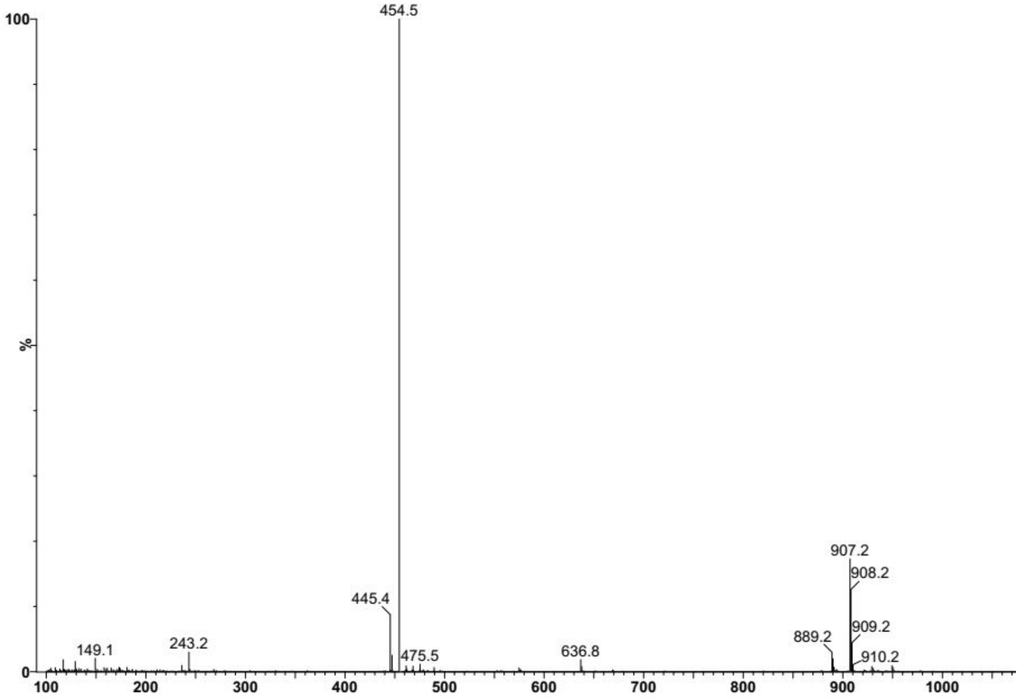

Supplementary Figure 21: ESI-MS spectrum of Lipid 34.

11-(8-((2-hexyldecanoyl)oxy)octyl)-2-methyl-7-oxo-2,6,8,11-tetraazanonadecan-19-yl 2-hexyldecanoate (Lipid 35)

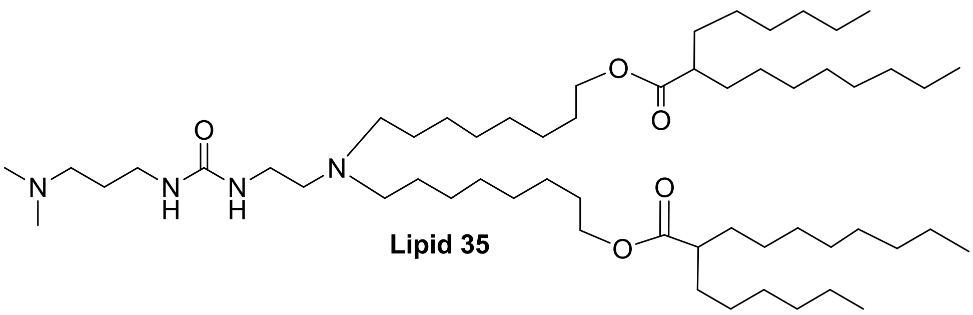

Lipid 35

To a solution of amine **4** (203 mg, 0.26 mmol, 1 equiv.) in anhydrous tetrahydrofuran (10 mL), CDI (166 mg, 1.02 mmol, 4 equiv.) and DMAP (6 mg, 0.05 mmol, 20 mol%) were added under argon atmosphere and stirred for the overnight at room temperature. After that, the tetrahydrofuran was evaporated on rotary evaporator and the reaction mixture dissolved in water and extracted with Ethyl acetate. The organic portion was washed with sat. $NaHCO_3$, water and brine solution respectively. Then, the solvent was dried over anhydrous Na_2SO_4 and evaporated rotary evaporator, and the residue was dried and dissolved in anhydrous tetrahydrofuran (10 mL). To this solution, 3-(Dimethylamino)-1-propylamine (64 µL, 0.51 mmol, 2 equiv.) and DMAP (6 mg, 0.05 mmol, 20 mol%) were added and left for the overnight stirring at ambient temperature under argon atmosphere. After that, THF was removed rotary evaporator and poured the reaction mixture in Ethyl acetate (100 ml) and washed with sat. $NaHCO_3$, water and brine solution respectively. The solvent was dried over anhydrous Na_2SO_4 and evaporated rotary evaporator, and the residue was purified by column chromatography using 0-15% Methanol in $CHCl_3$ to obtain **Lipid 35** (187 mg, 79%) as pale yellow color oil.

^{1}H NMR (400 MHz, $CDCl_3$): δ 6.01-5.58 (2 H, br), 4.06 (4 H, t, J = 6.8 Hz), 3.37-3.28 (2, m), 3.25 (2 H, t, J = 6.4 Hz), 2.81-2.69 (2 H, br), 2.69-2.53 (6 H, br), 2.48 (2 H, t, J = 6.8 Hz), 2.39-2.22 (2 H, m), 2.32 (6 H, s), 1.72 (2 H, quint, J = 6.8 Hz), 1.66-1.48 (12 H, m), 1.48-1.37 (4 H, m), 1.37-1.16 (56 H, m), 0.87 (12 H, t, J = 6.8 Hz).

ESI-MS: m/z 922.2 $[M+1]^+$; 462.1 $[M/2+1]^+$

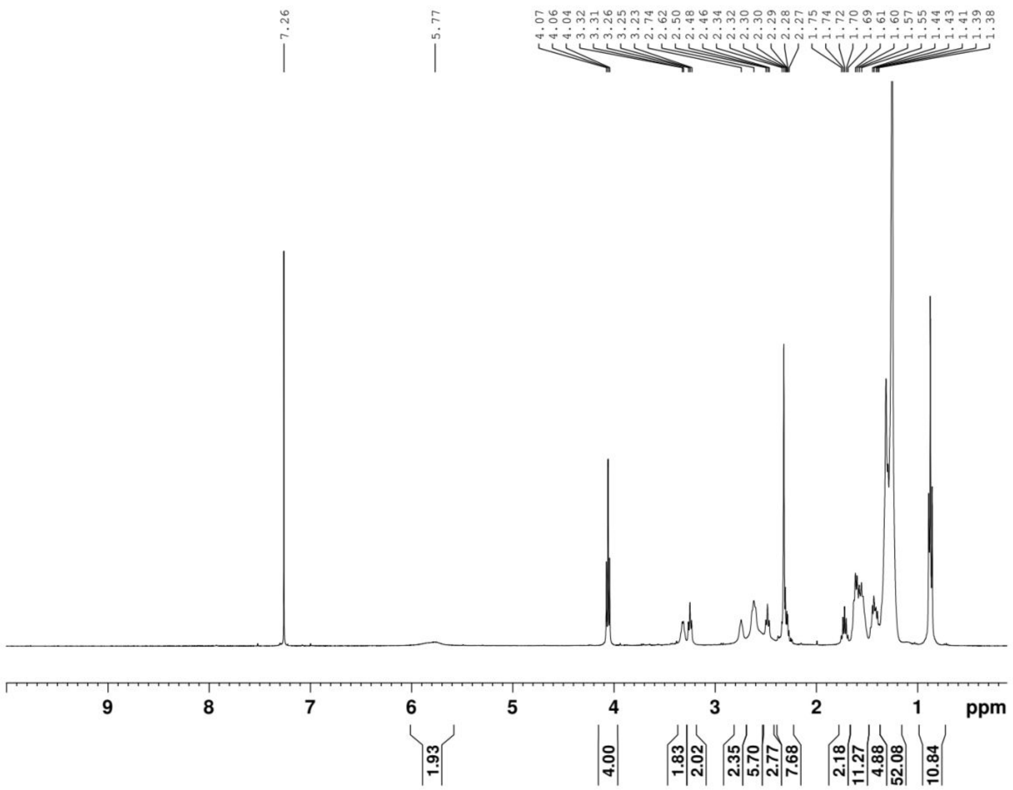

Supplementary Figure 22: ^{1}HNMR Spectrum of Lipid 35.

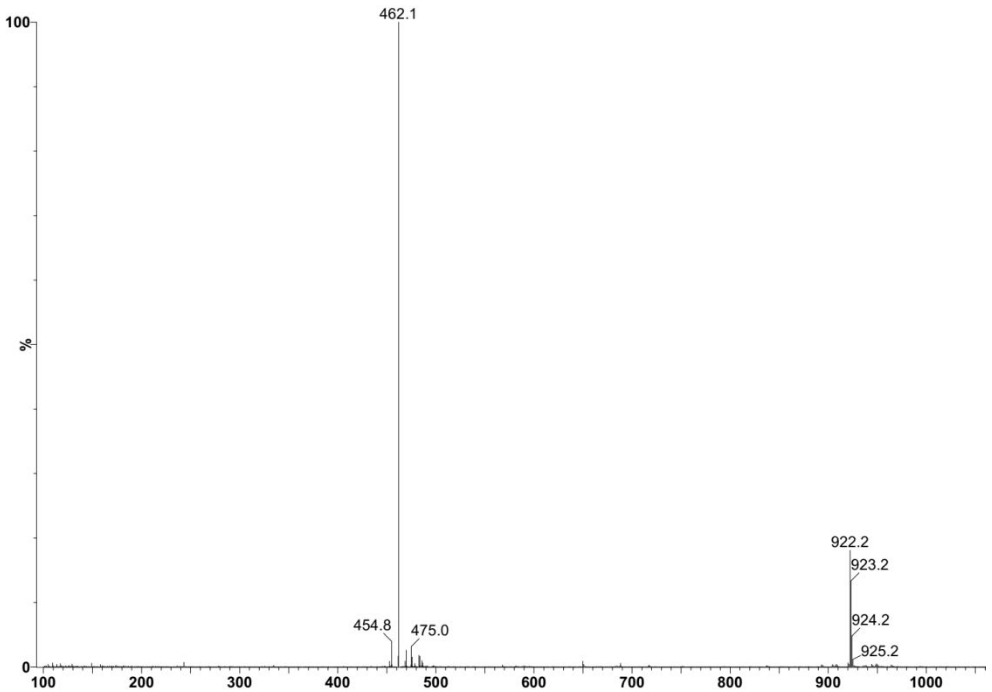

Supplementary Figure 23: ESI-MS spectrum of Lipid 35.

((3-((3-(Dimethylamino)propyl)amino)-3-oxopropyl)azanediyl)bis(octane-8,1-diyl) bis(2-hexyldecanoate)(Lipid 36)

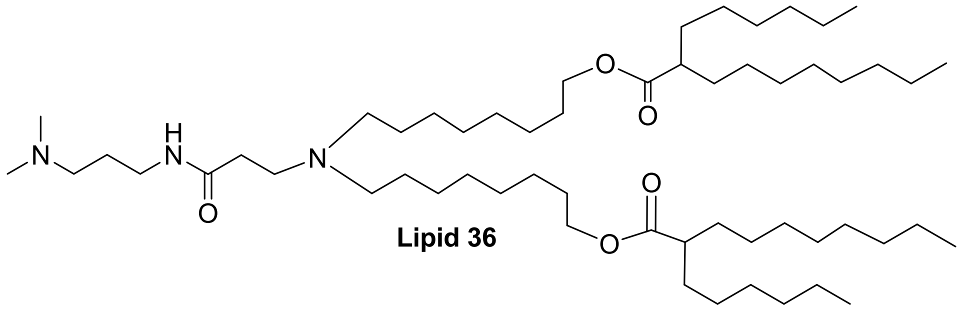

The acid **3** (220 mg, 0.27 mmol, 1 equiv.), 3-(Dimethylamino)-1-propylamine (67 µL, 0.53 mmol, 2 equiv.) and DMAP (6 mg, 0.05 mmol, 20 mol%) were dissolved in anhydrous Dichloromethane (10 mL) under argon atmosphere and stirred for 2 min. Then, EDC (102 mg, 0.53 mmol, 2 equiv.) was added it and stirred for 6 hr at room temperature under argon atmosphere. After that, the reaction was quenched with sat. $NaHCO_3$ solution and extracted with ethyl acetate. Then the organic portion was washed water, brine solution, and dried over anhydrous Na_2SO_4 respectively. The solvent was evaporated on rotary evaporator, and the residue was purified by column chromatography using 0-15% Methanol in $CHCl_3$ to obtain **Lipid 36** (180 mg, 74%) as pale yellowish liquid.

^{1}H NMR (400 MHz, $CDCl_3$): δ 8.34 (1 H, br), 4.04 (4 H, t, J = 6.8 Hz), 3.28 (2 H, q, J = 6.4 Hz), 2.89-2.74 (2 H, br), 2.66-2.49 (6 H, br), 2.48-2.42 (2 H, m), 2.37 (6 H, s), 2.33-2.25 (2 H, m), 1.76 (2 H, quint, J = 6.8 Hz), 1.66-1.47 (12 H, m), 1.47-1.36 (4 H, m), 1.36-1.16 (56 H, m), 0.87 (12 H, t, J = 6.8 Hz).

ESI-MS: m/z 907.3 $[M+1]^+$; 454.5 $[M/2+1]^+$

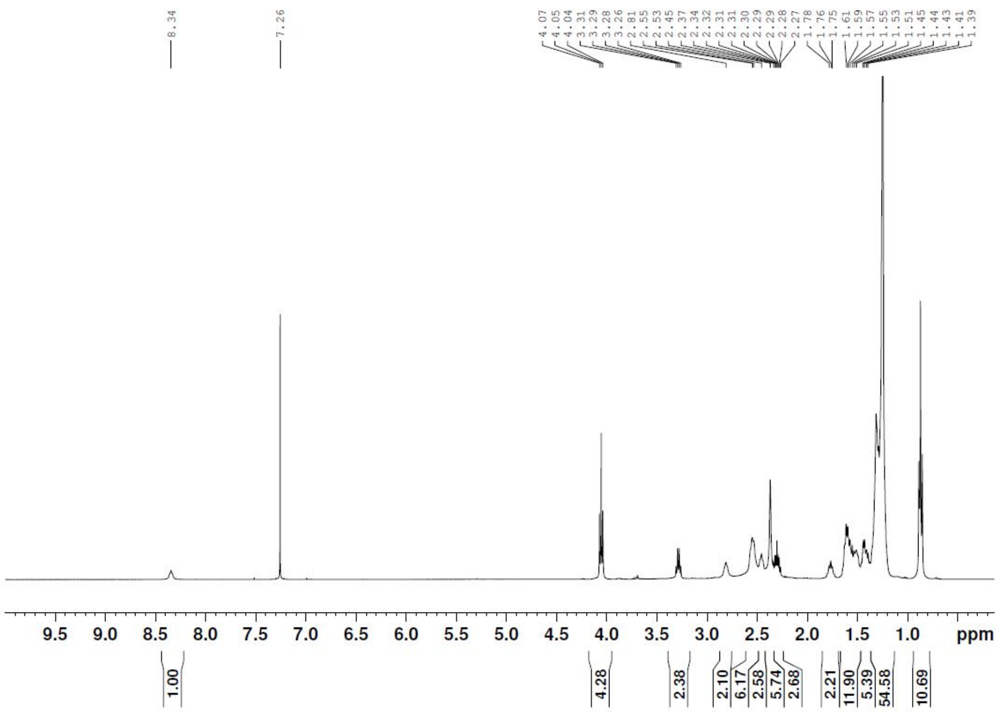

Supplementary Figure 24: ^{1}HNMR Spectrum of Lipid 36.

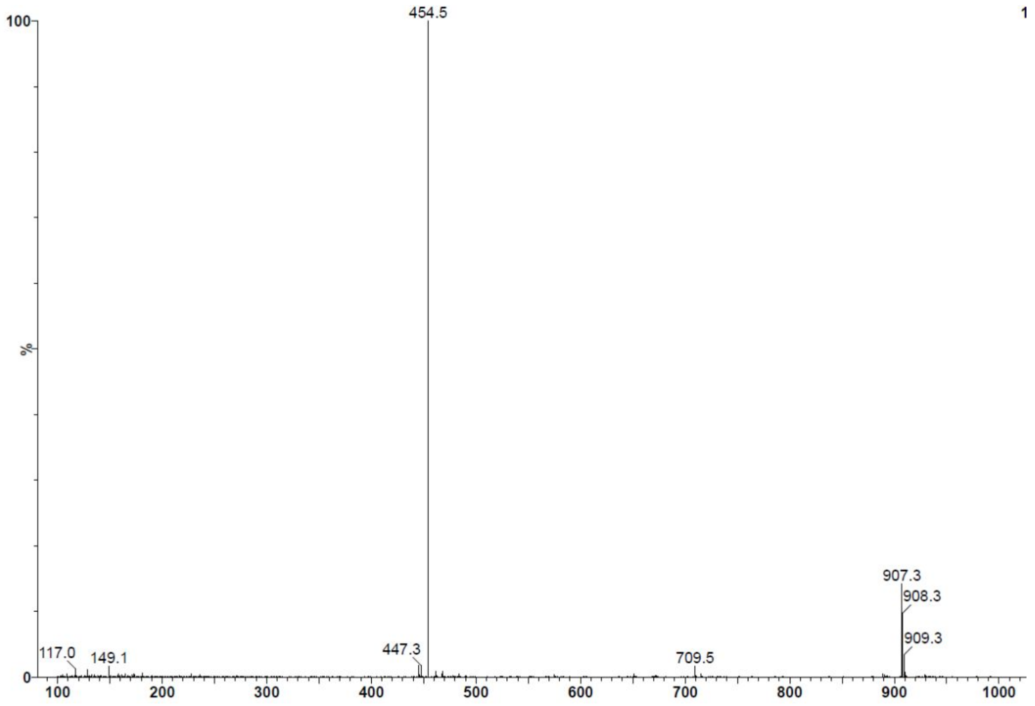

Supplementary Figure 25: ESI-MS spectrum of Lipid 36.

((2-((3-(4-Methylpiperazin-1-yl)propanoyl)oxy)ethyl)azanediyl)bis(octane-8,1-diyl) bis(2-hexyldecanoate)(Lipid 37)

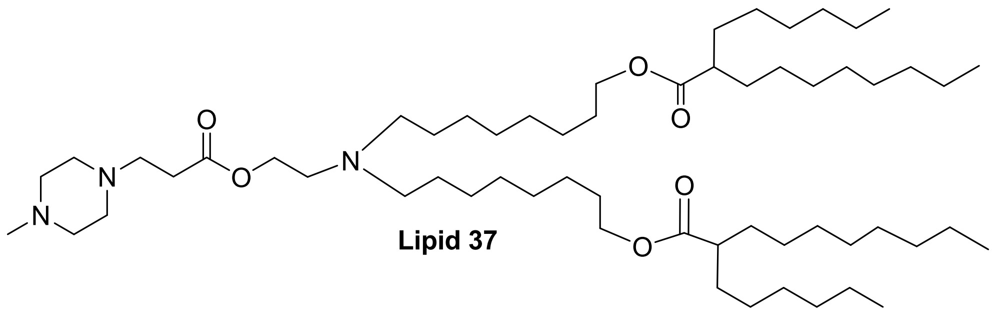

Lipid 37

The alcohol **2** (195 mg, 0.24 mmol, 1 equiv.), 3-(4-Methylpiperazin-1-yl)propanoic acid (84 mg, 0.49 mmol, 2 equiv.), EDC.HCl (93 mg, 0.49 mmol, 2 equiv.) and DMAP (6 mg, 0.05 mmol, 20 mol%) were dissolved in dry CH_2Cl_2 (15 mL) under argon atmosphere and stirred for 12 hr. at room temperature. After that, the reaction was quenched with sat. $NaHCO_3$ followed by extract with ethyl acetate (3 times). Then the organic portion was washed with water (2 times) and brine solution (1 time), and dried over anhydrous Na_2SO_4. The solvent was evaporated and the residue was purified by column chromatography using 0-15% isopropanol in $CHCl_3$ to bestow **Lipid 37** (193 mg, 83%) as pale yellow color oil.

^{1}H NMR (400 MHz, $CDCl_3$): δ 4.24-4.10 (2 H, br), 4.05 (4 H, t, J = 6.8 Hz), 2.73 (4 H, t, J = 7.2 Hz), 2.66-2.55 (4 H, m), 2.50 (8 H, t, J = 7.2 Hz), 2.38 (3 H, br), 2.34-2.22 (4 H, m), 1.69-1.50 (8 H, m), 1.49-1.37 (8 H, m), 1.36-1.13 (56 H, m), 0.87 (12 H, t, J = 6.8 Hz).

ESI-MS: m/z 949.2 $[M+1]^+$; 475.6 $[M/2+1]^+$

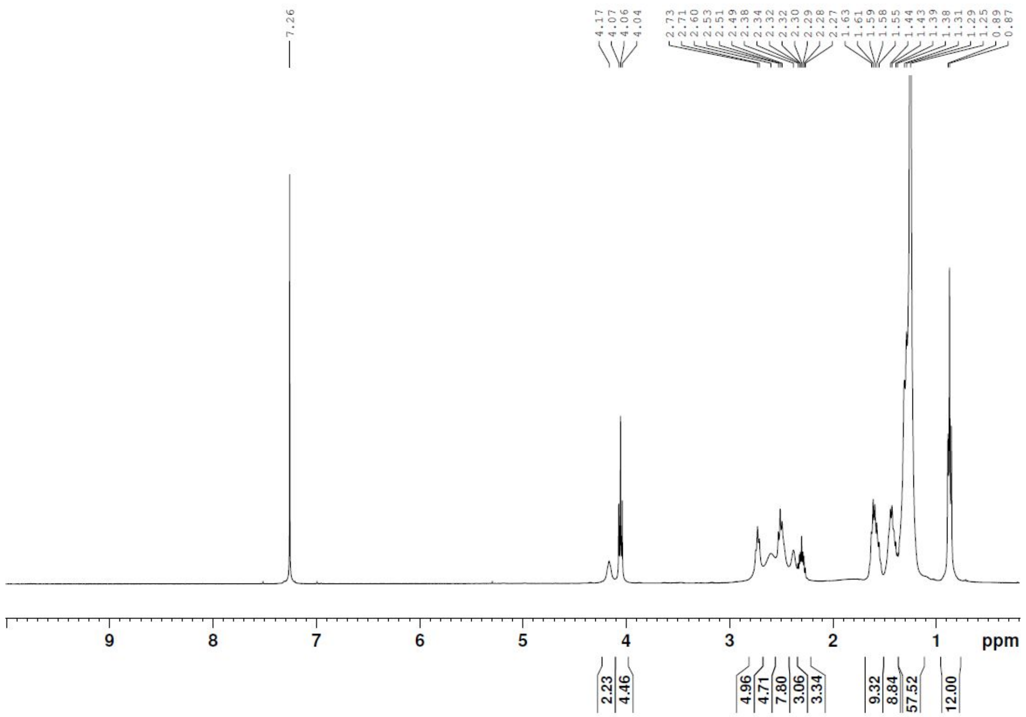

Supplementary Figure 26: ¹HNMR Spectrum of Lipid 37.

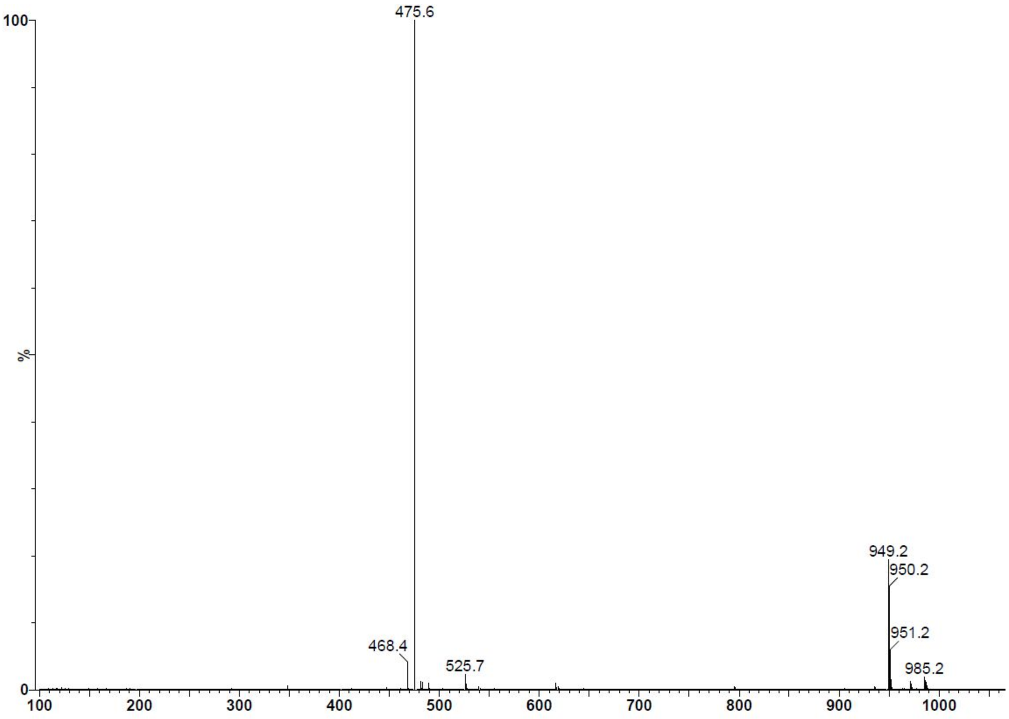

Supplementary Figure 27: ESI-MS spectrum of Lipid 38.

((3-(2-(4-Methylpiperazin-1-yl)ethoxy)-3-oxopropyl)azanediyl)bis(octane-8,1-diyl) bis(2-hexyldecanoate) (Lipid 38)

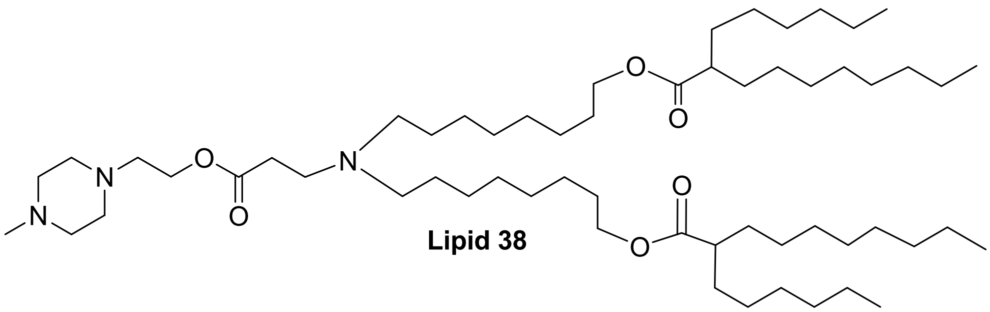

Lipid 38

The acid **3** (323 mg, 0.39 mmol, 1 equiv.), 4-Methylpiperazine-1-ethanol (113 μL, 0.78 mmol, 2 equiv.) and DMAP (10 mg, 0.08 mmol, 20 mol%) were dissolved in anhydrous Dichloromethane (20 mL) under argon atmosphere and stirred for 2 min. Then, EDC (150 mg, 0.78 mmol, 2 equiv.) was added it and stirred for 6 hr. at room temperature under argon atmosphere. After that, the reaction was quenched with sat. $NaHCO_3$ solution and extracted with ethyl acetate. Then the organic portion was washed water, brine solution, and dried over anhydrous Na_2SO_4 respectively. The solvent was evaporated on rotary evaporator, and the residue was purified by column chromatography using 0-15% isopropanol in $CHCl_3$ to obtain **Lipid 38** (205 mg, 55%) as pale yellowish liquid.

^{1}H NMR (400 MHz, $CDCl_3$): δ 4.20 (2 H, t, J = 6.0 Hz), 4.05 (4 H, t, J = 6.8 Hz), 2.91-2.72 (2 H, br), 2.70-2.36 (12 H, br), 2.64 (2 H, t, J = 6.0 Hz), 2.36-2.18 (7 H, m), 1.68-1.51 (8 H, m), 1.50-1.36 (6 H, m), 1.36-1.14 (58 H, m), 0.87 (12 H, t, J = 6.8 Hz).

ESI-MS: m/z 949.2 $[M+1]^+$; 475.5 $[M/2+1]^+$

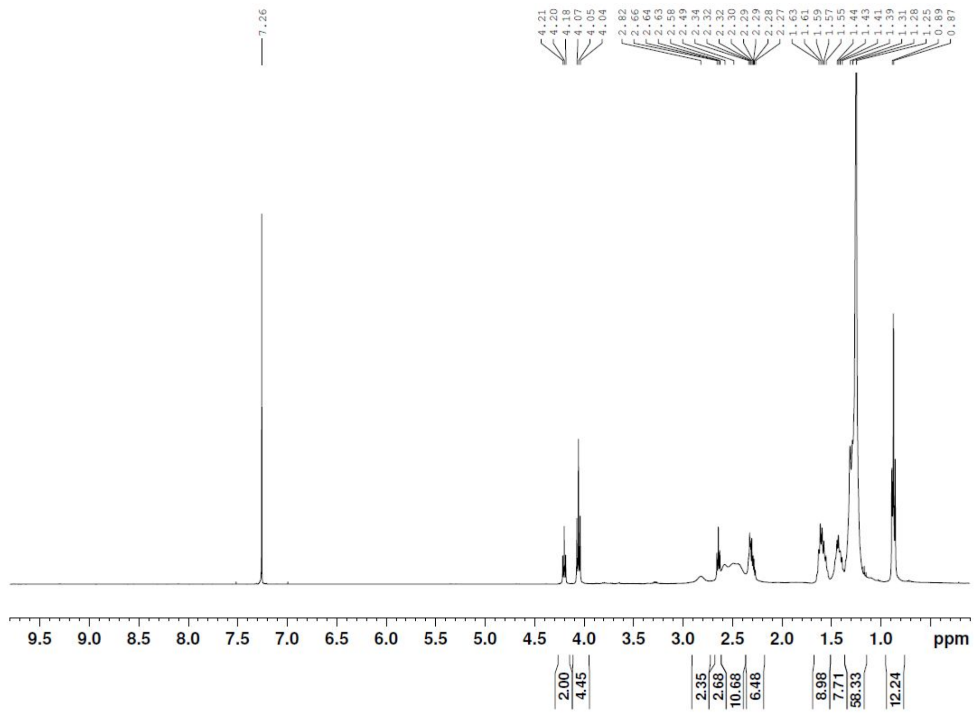

Supplementary Figure 28: ^{1}HNMR Spectrum of Lipid 38.

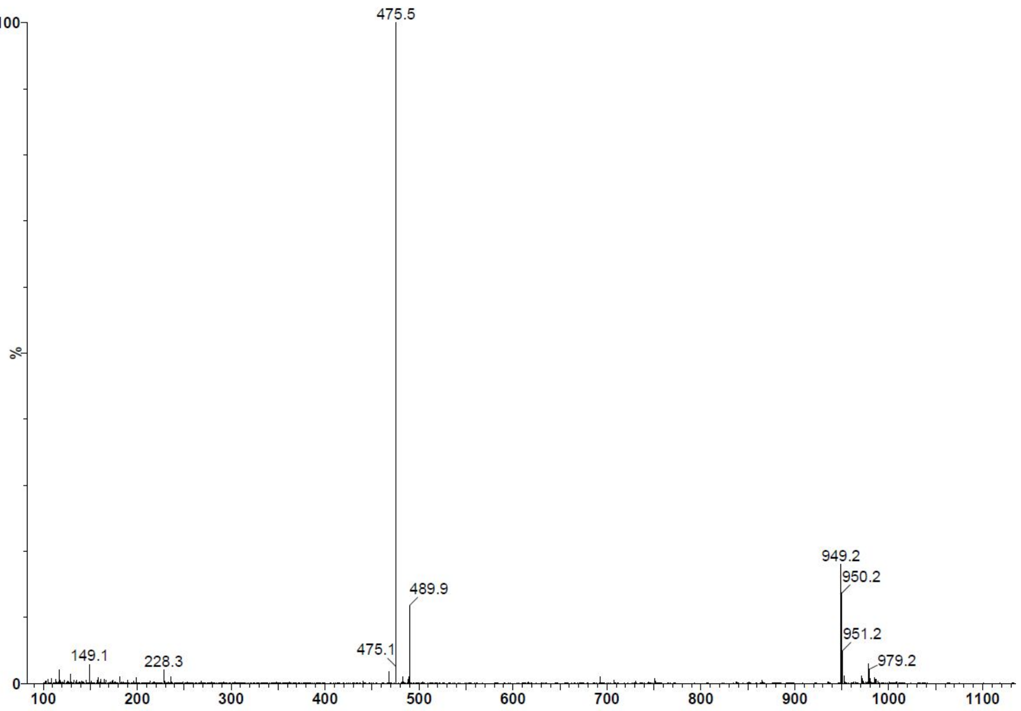

Supplementary Figure 29: ESI-MS spectrum of Lipid 38.

((2-(((2-(4-methylpiperazin-1-yl)ethoxy)carbonyl)oxy)ethyl)azanediyl)bis(octane-8,1-diyl) bis(2-hexyldecanoate)(Lipid 39)

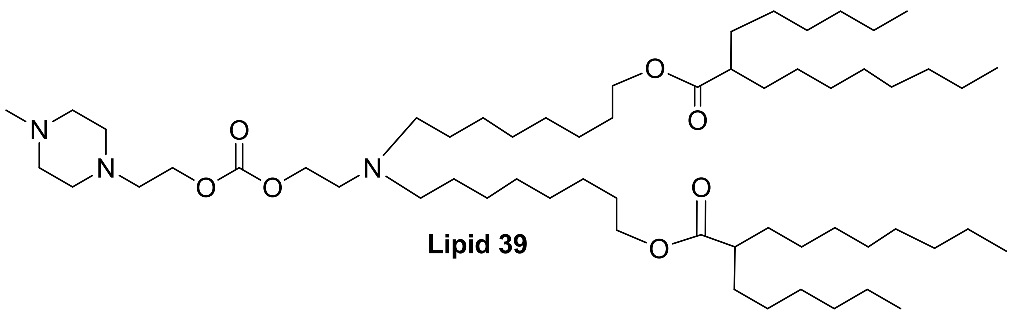

Lipid 39

To a solution of ethanolamine **2** (250 mg, 0.314 mmol, 1 equiv.) in anhydrous tetrahydofuran (10 mL), CDI (204 mg, 1.26 mmol, 4 equiv.) and DMAP (8 mg, 0.06 mmol, 20 mol%) were added under argon atmosphere and stirred for 6 hr. at room temperature. Then, 4-Methylpiperazine-1-ethanol (91 mg, 0.63 mmol, 2 equiv.) was added and left for the overnight stirring at 60 °C under argon atmosphere. After that, THF was removed rotary evaporator and poured the reaction mixture in ethyl acetate (100 ml) and washed with sat. $NaHCO_3$, water and brine solution respectively. The solvent was dried over anhydrous Na_2SO_4 and evaporated rotary evaporator, and the residue was purified by column chromatography using 0-15% Isopropanol in $CHCl_3$ to bestow **Lipid 39** (198 mg, 66%) as pale yellowish liquid.

^1H NMR (400 MHz, $CDCl_3$): δ 4.24 (2 H, t, J = 6.0 Hz), 4.18 (2 H, t, J = 6.4 Hz), 4.05 (4 H, t, J = 6.8 Hz), 2.78-2.70 (2 H, br), 2.67 (2 H, t, J = 6.0 Hz), 2.65-2.51 (6 H, br), 2.51-2.40 (6 H, br), 2.40-2.22 (5 H, m), 1.69-1.49 (8 H, m), 1.48-1.36 (8 H, m), 1.36-1.14 (56 H, m), 0.87 (12 H, t, J = 6.8 Hz).

ESI-MS: m/z 965.2 $[M+1]^+$; 483.5 $[M/2+1]^+$

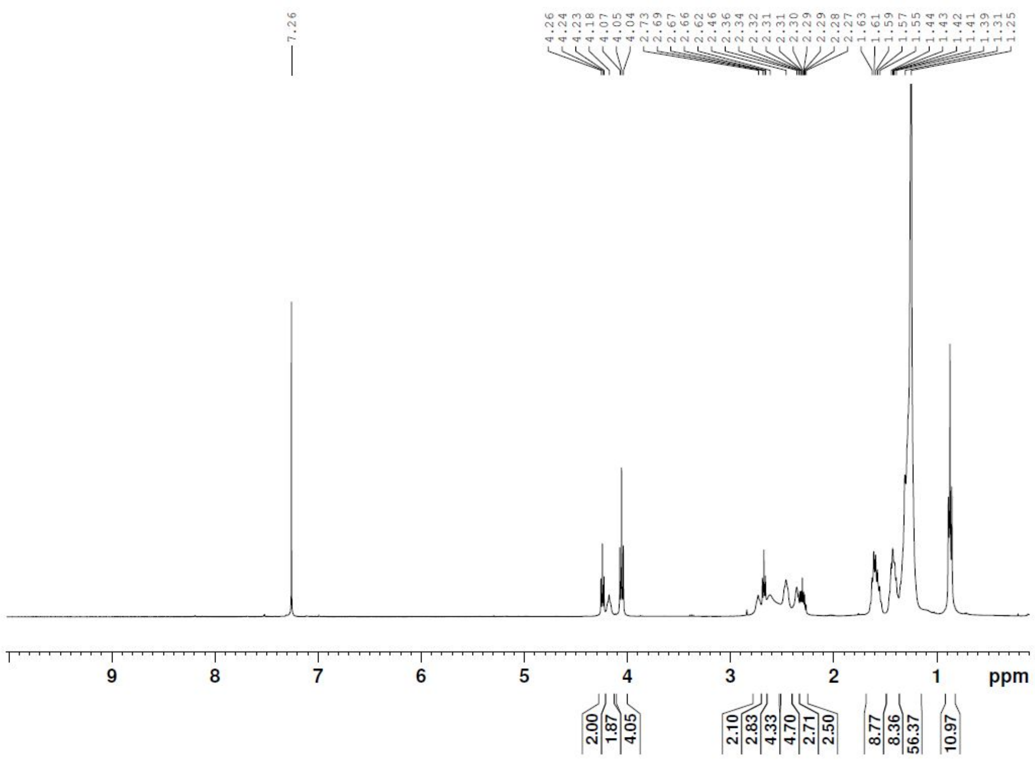

Supplementary Figure 30: ^{1}HNMR Spectrum of Lipid 39.

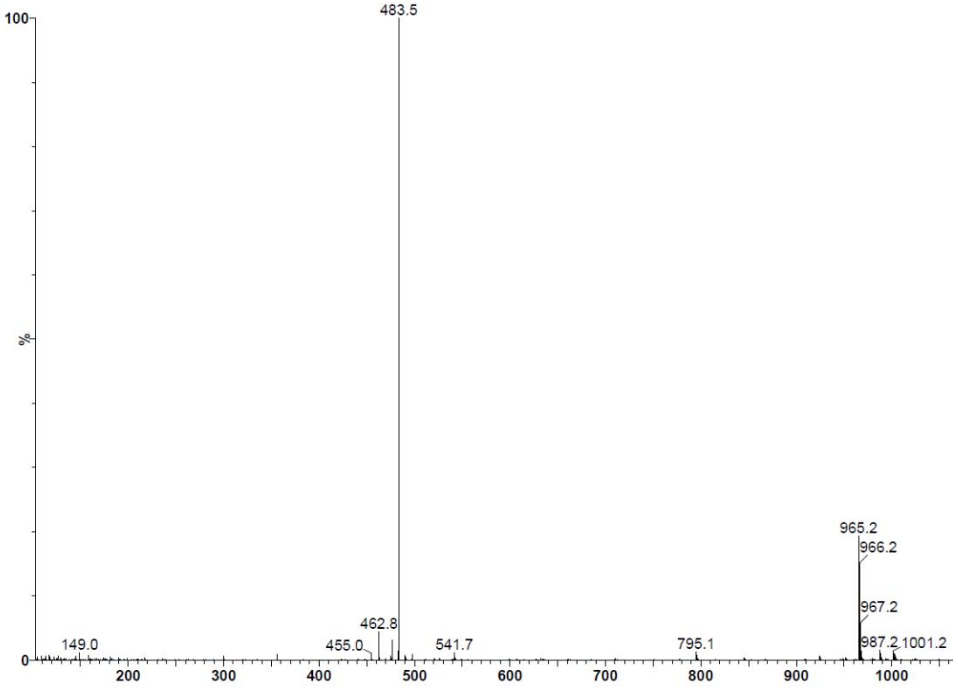

Supplementary Figure 31: ESI-MS spectrum of Lipid 39.

((2-(3-(4-methylpiperazin-1-yl)propanamido)ethyl)azanediyl)bis(octane-8,1-diyl) bis(2-hexyldecanoate)(Lipid 40)

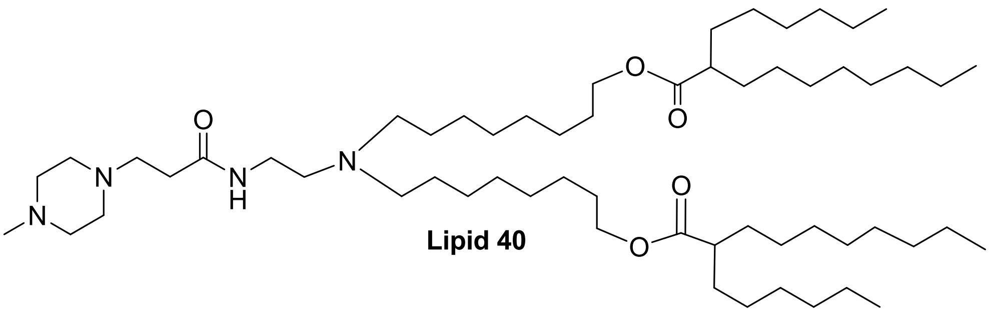

The amine **4** (235 mg, 0.30 mmol, 1 equiv.), 3-(4-Methylpiperazin-1-yl)propanoic acid (102 mg, 0.60 mmol, 2 equiv.), EDC.HCl (113 mg, 0.60 mmol, 2 equiv.) and DMAP (7 mg, 0.06 mmol, 20 mol%) were dissolved in dry CH_2Cl_2 (15 mL) under argon atmosphere and stirred for 6 hr. at room temperature. After that, the reaction was quenched with sat. $NaHCO_3$ followed by extract with Ethyl acetate (3 times). Then the organic portion was washed with water (2 times) and brine solution (1 time), and dried over anhydrous Na_2SO_4. The solvent was evaporated and the residue was purified by column chromatography using 0-15% methanol in dichloromethane to obtain **Lipid 40** (178 mg, 64%) as pale yellowish oil.

^{1}H NMR (400 MHz, $CDCl_3$): δ 4.06 (4 H, t, J = 6.8 Hz), 3.51-3.30 (2 H, br), 2.78-2.44 (10 H, br), 2.66 (2 H, t, J= 6.8 Hz), 2.40 (2 H, t, J= 6.4 Hz), 2.36-2.25 (2 H, m), 2.32 (3 H, s), 2.15-1.91 (4, br), 1.68-1.47 (10 H, m), 1.48-1.37 (6 H, m), 1.36-1.12 (56 H, m), 0.87 (12 H, t, J = 6.8 Hz).

ESI-MS: m/z 948.2 $[M+1]^+$; 475.1 $[M/2+1]^+$

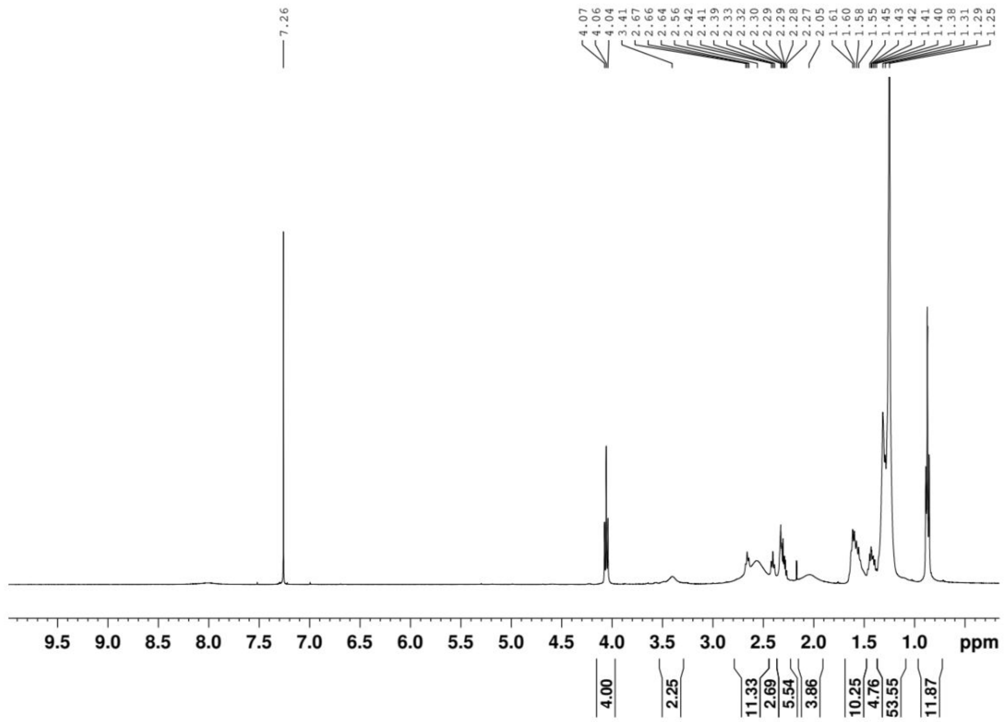

Supplementary Figure 32: ^{1}HNMR Spectrum of Lipid 40.

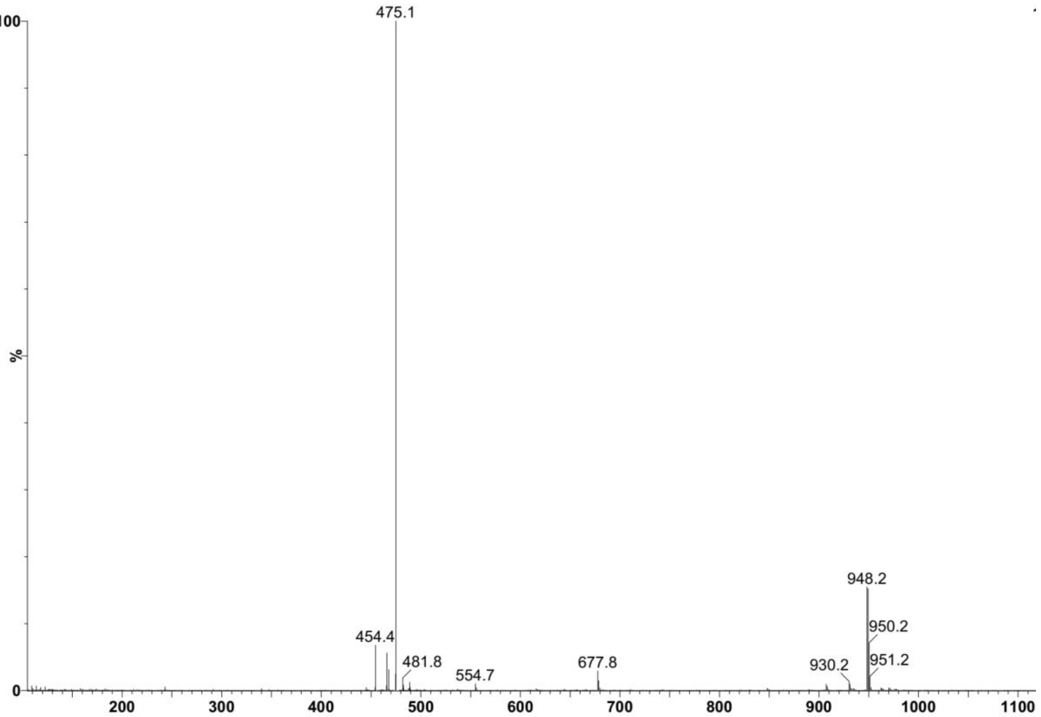

Supplementary Figure 33: : ESI-MS spectrum of Lipid 40.

((2-(3-(2-(4-methylpiperazin-1-yl)ethyl)ureido)ethyl)azanediyl)bis(octane-8,1-diyl) bis(2-hexyldecanoate)(Lipid 41)

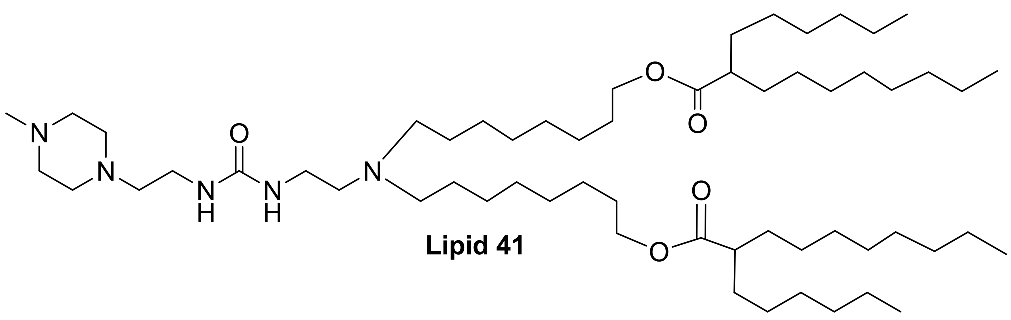

Lipid 41

To a solution of amine **4** (238 mg, 0.3 mmol, 1 equiv.) in anhydrous tetrahydrofuran (10 mL), CDI (195 mg, 1.2 mmol, 4 equiv.) and DMAP (7 mg, 0.06 mmol, 20 mol %) were added under argon atmosphere and stirred for the overnight at room temperature. After that, the tetrahydrofuran was evaporated on rotary evaporator and the reaction mixture dissolved in water and extracted with Ethyl acetate. The organic portion was washed with sat. $NaHCO_3$, water and brine solution respectively. Then, the solvent was dried over anhydrous Na_2SO_4 and evaporated rotary evaporator, and the residue was dried and dissolved in anhydrous tetrahydrofuran (10 mL). To this solution, 2-(4-Methyl-piperazin-1-yl)-ethylamine (67 µL, 0.45 mmol, 1.5 equiv.) and DMAP (7 mg, 0.06 mmol, 20 mol %) were added and left for the overnight stirring at ambient temperature under argon atmosphere. After that, THF was removed rotary evaporator and poured the reaction mixture in Ethyl acetate (100 ml) and washed with sat. $NaHCO_3$, water and brine solution respectively. The solvent was dried over anhydrous Na_2SO_4 and evaporated rotary evaporator, and the residue was purified by column chromatography using 0-20% Methanol in $CHCl_3$ to obtain **Lipid 41** (178 mg, 62 %) as pale yellowish liquid.

^{1}H NMR (400 MHz, $CDCl_3$): δ 5.45-5.26 (1 H, br), 4.06 (4 H, t, J = 6.8 Hz), 3.49-3.32 (2 H, br), 3.28 (2 H, q, J = 6.8 Hz), 2.94-2.77 (2 H, br), 2.77-2.62 (4 H, br), 2.62-2.32 (6 H, br), 2.50 (4 H, t, J = 6.0 Hz), 2.36-2.25 (2 H, m), 2.32 (3 H, s), 1.68-1.49 (12 H, m), 1.49-1.37 (6 H, m), 1.37-1.14 (54 H, m), 0.87 (12 H, t, J = 6.8 Hz).

ESI-MS: m/z 963.3 $[M+1]^+$; 482.6 $[M/2+1]^+$

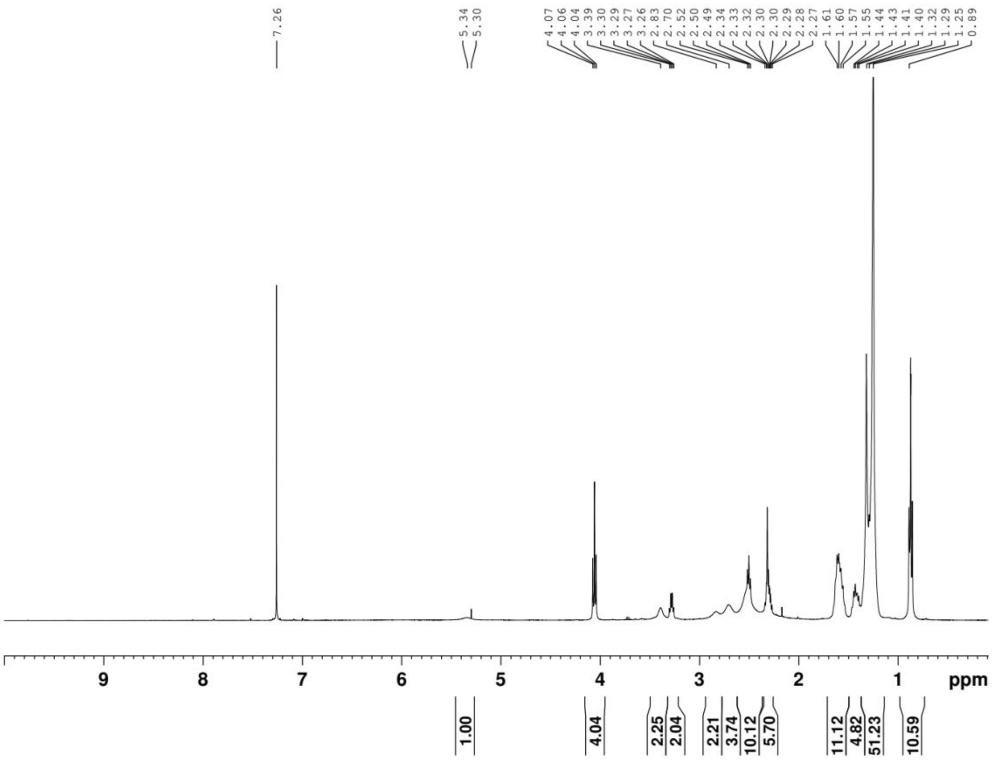

Supplementary Figure 34: ^{1}HNMR Spectrum of Lipid 41.

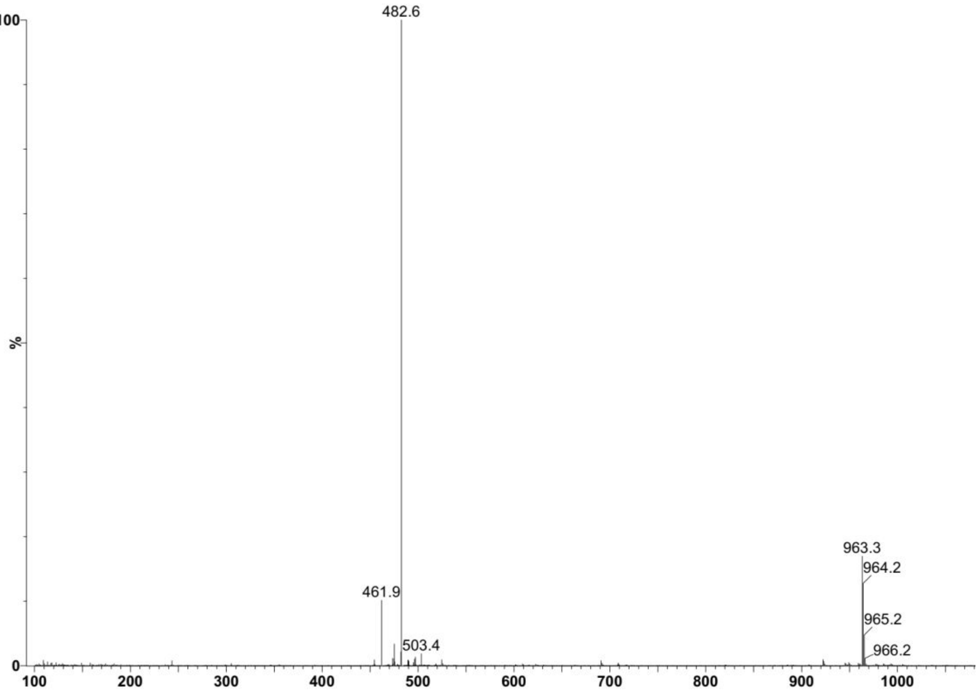

Supplementary Figure 35: ESI-MS spectrum of Lipid 41.

((3-((2-(4-Methylpiperazin-1-yl)ethyl)amino)-3-oxopropyl)azanediyl)bis(octane-8,1-diyl) bis(2-hexyldecanoate)(Lipid 42)

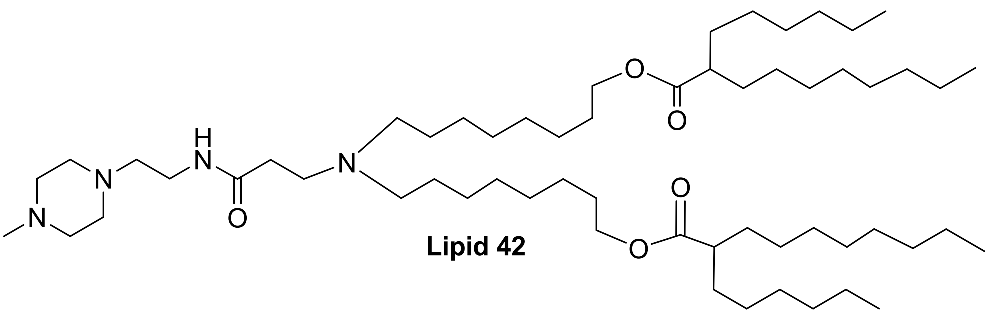

Lipid 42

The acid **3** (180 mg, 0.22 mmol, 1 equiv.), 2-(4-Methyl-piperazin-1-yl)-ethylamine (50 µL, 0.33 mmol, 1.5 equiv.) and DMAP (5 mg, 0.04 mmol, 20 mol%) were dissolved in anhydrous Dichloromethane (10 mL) under argon atmosphere and stirred for 2 min. Then, EDC (83 mg, 0.44 mmol, 2 equiv.) was added it and stirred for 6 hr at room temperature under argon atmosphere. After that, the reaction was quenched with sat. $NaHCO_3$ solution and extracted with ethyl acetate. Then the organic portion was washed water, brine solution, and dried over anhydrous Na_2SO_4 respectively. The solvent was evaporated on rotary evaporator, and the residue was purified by column chromatography using 0-15% Methanol in $CHCl_3$ to obtain **Lipid 42** (130 mg, 72%) as pale yellowish liquid.

^{1}H NMR (400 MHz, $CDCl_3$): δ 4.05 (4 H, t, J = 6.8 Hz), 3.35 (2 H, q, J = 6.0 Hz), 2.98-2.34 (14 H, br), 2.36-2.23 (2 H, m), 2.32 (3 H, m), 1.68-1.47 (10 H, m), 1.47-1.36 (6 H, m), 1.36-1.14 (56 H, m), 0.87 (12 H, t, J = 6.8 Hz).

ESI-MS: m/z 948.3 $[M+1]^+$; 475.0 $[M/2+1]^+$

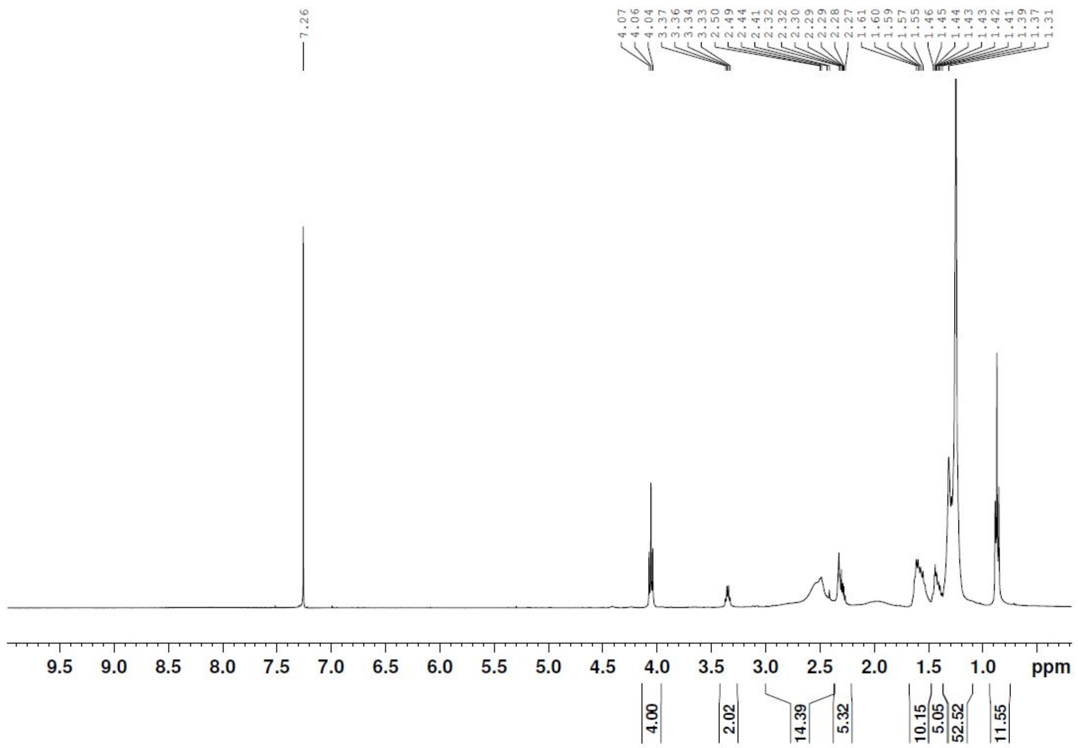

Supplementary Figure 36: ^{1}HNMR Spectrum of Lipid 42.

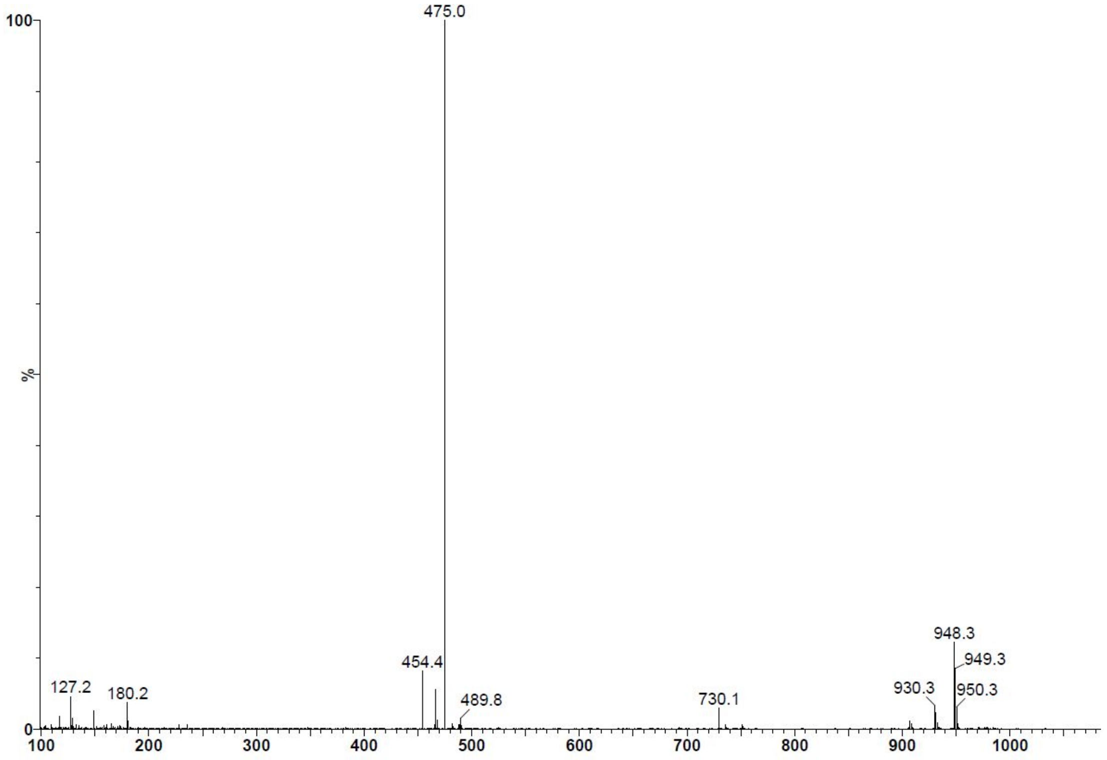

Supplementary Figure 37: ESI-MS spectrum of Lipid 42.
